# Supplementary figures and images for: Osteocalcin is necessary for the alignment of apatite crystallites, but not glucose metabolism, testosterone synthesis, or muscle mass
Source: PLoS Genet. 2020 May 28;16(5):e1008586. doi: 10.1371/journal.pgen.1008586 (PMC7255595; doi:10.1371/journal.pgen.1008586)

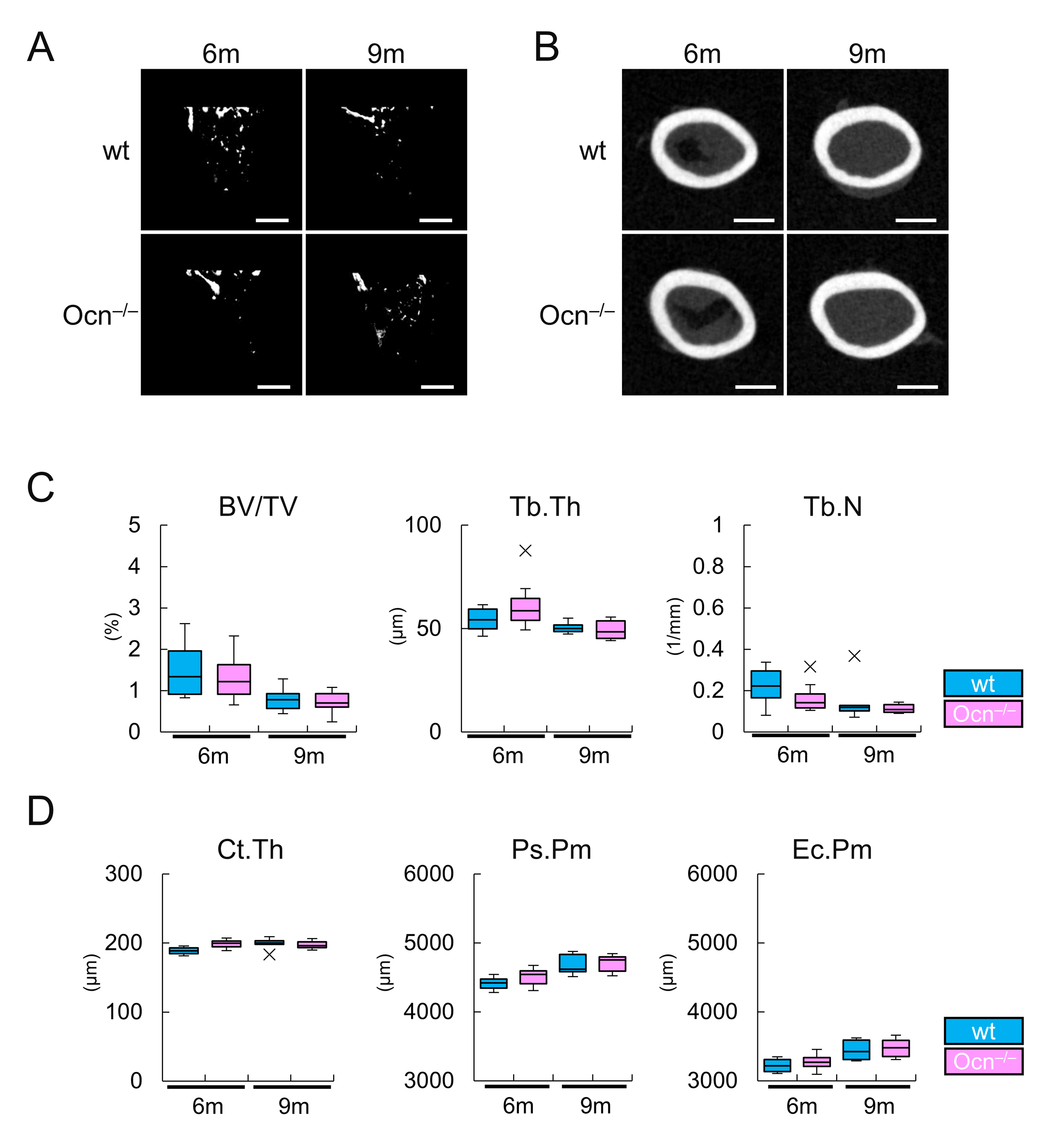

Supplement: S1 Fig — (A and B). μ-CT images of femoral distal metaphyses (A) and mid-diaphyses (B). Scale bars = 500 μm. (C) Trabecular bone parameters, including the trabecular bone volume (BV/TV), trabecular thickness (Tb.Th), and trabecular number (Tb.N). (D) Cortical bone parameters including cortical thickness (Ct. Th), the periosteal perimeter (Ps.Pm), and endocortical perimeter (Ec.Pm). wt (n = 6), Ocn–/–(n = 8) at 6m; wt (n = 5), Ocn–/–(n = 6) at 9m. X symbols in box plots show outliers. (TIF) (TIF) [file pgen.1008586.s001.tif]

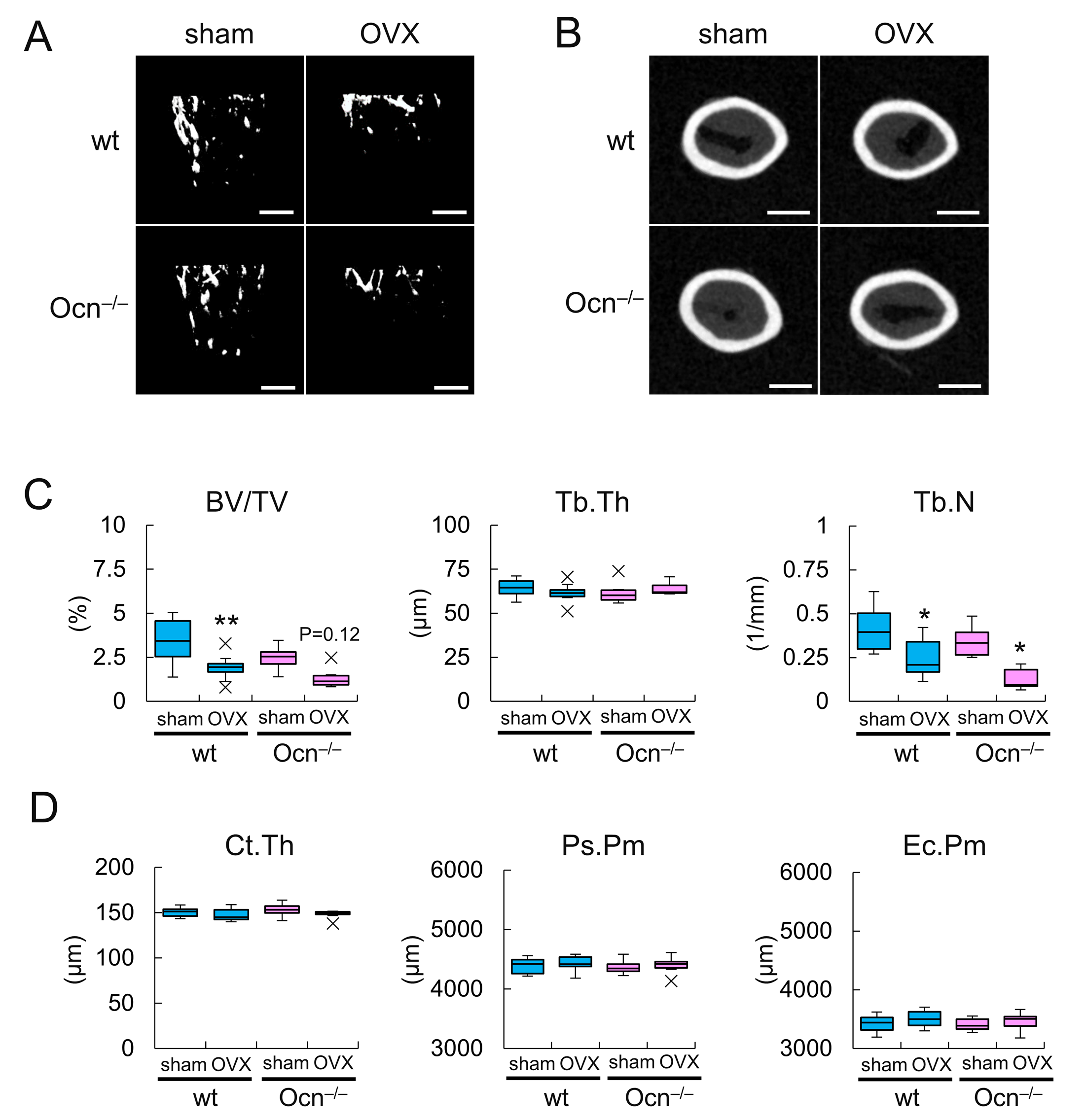

Supplement: S2 Fig — Sham operation or ovariectomy (OVX) was performed at 5 weeks of age and mice were analyzed at 11 weeks of age. (A-D) μ-CT analyses of femurs in wild-type and Ocn–/–mice with sham operation or OVX. μ-CT images of femoral distal metaphyses (A) and mid-diaphyses (B) are shown. Scale bars = 500 μm. C, Trabecular bone parameters, including the trabecular bone volume (BV/TV), trabecular thickness (Tb.Th), and trabecular number (Tb.N). D, Cortical bone parameters including cortical thickness (Ct. Th), the periosteal perimeter (Ps.Pm), and endocortical perimeter (Ec.Pm). wt sham: n = 9, wt OVX: n = 8, Ocn–/–sham: n = 6, Ocn–/–OVX: n = 7. X symbols in box plots show outliers. (TIF) (TIF) [file pgen.1008586.s002.tif]

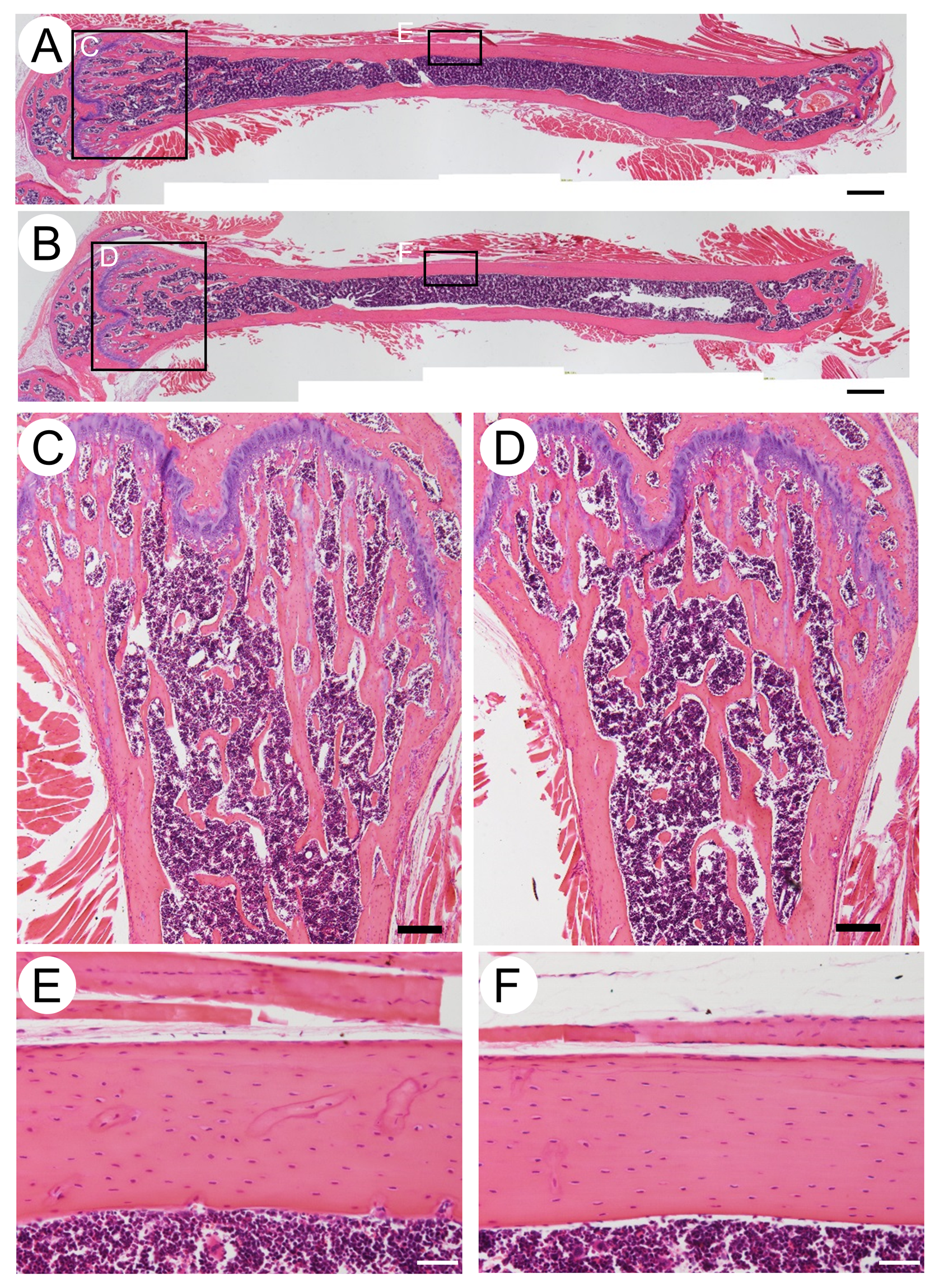

Supplement: S3 Fig — (A, C, E) Wild-type mice. (B, D, F) Ocn–/–mice. The boxed regions in A are magnified in C and E, and those in B are magnified in D and F. Scale bars: 0.5 mm (A, B), 200 μm (C, D), 50 μm (E, F). (TIF) (TIF) [file pgen.1008586.s003.tif]

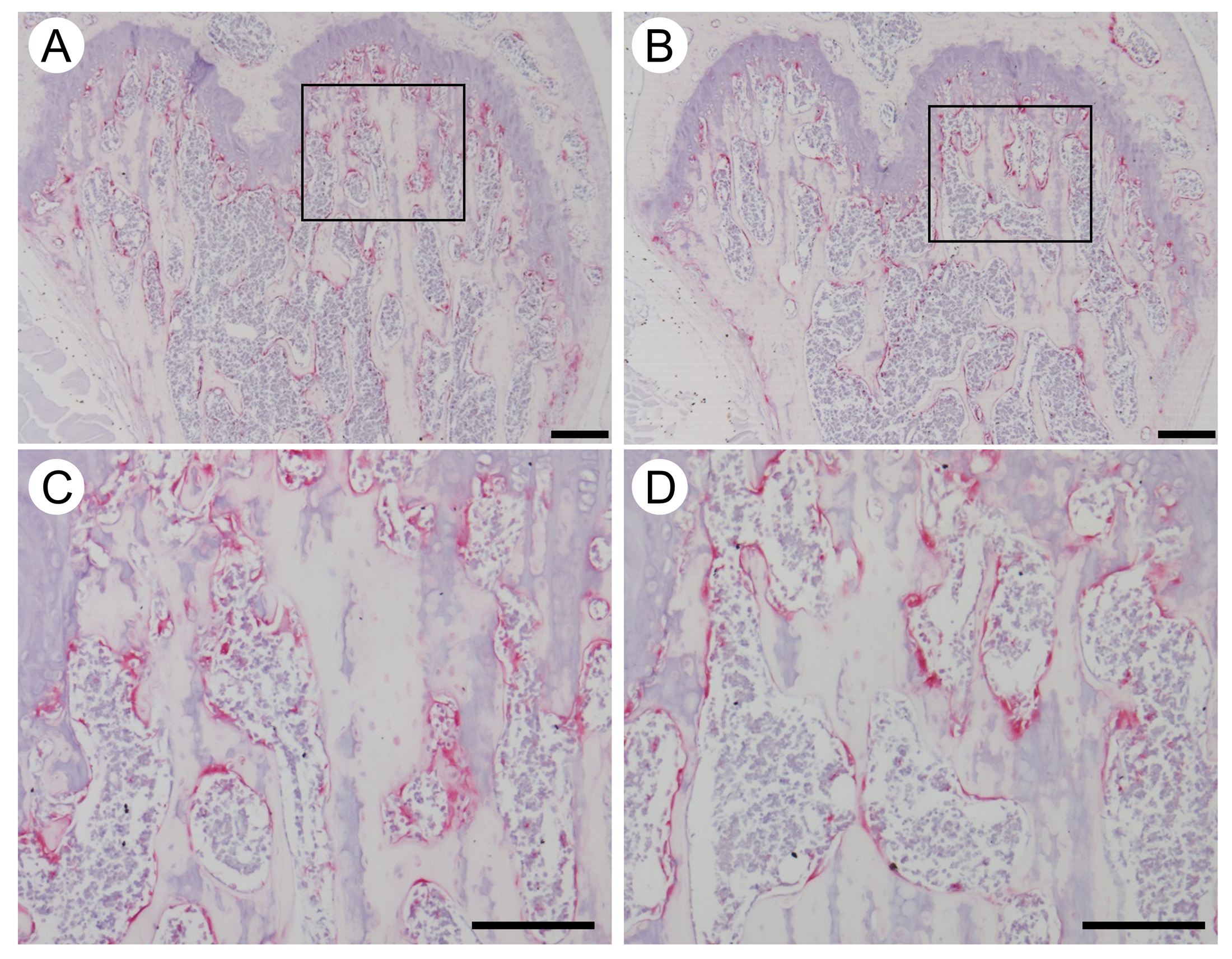

Supplement: S4 Fig — Sections of femurs in wild-type (A, C) and Ocn–/–(B, D) mice at 14 weeks of age were stained with TRAP. The boxed regions in A and B are magnified in C and D, respectively. Scale bars: 200 μm (A, B), 100 μm (C, D). (TIF) (TIF) [file pgen.1008586.s004.tif]

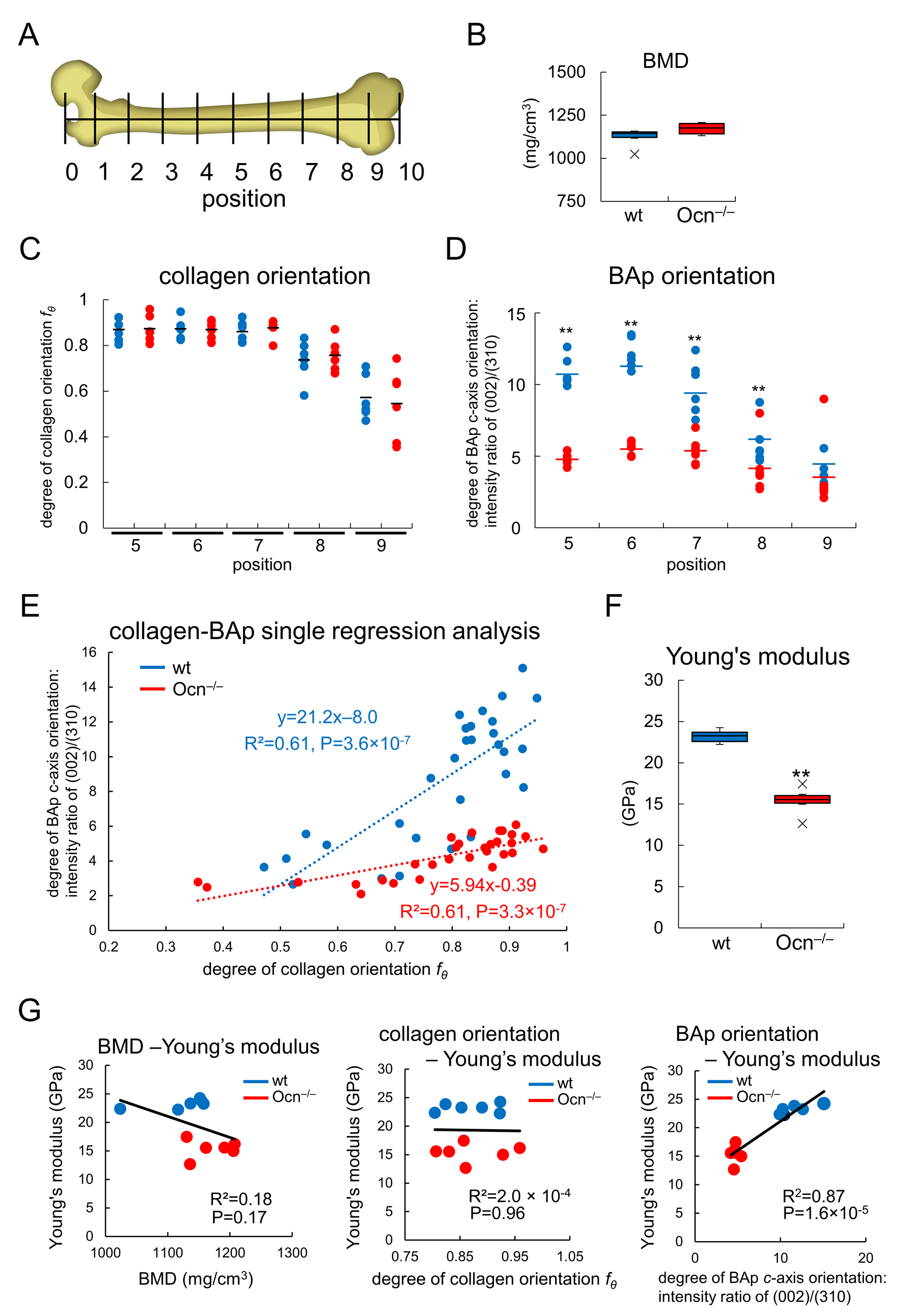

Supplement: S5 Fig — (A) Schematic presentation of analyzed positions. (B) BMD at position 5. (C) Collagen orientation degree. (D) BAp c-axis orientation degree. (E) Single regression analysis of the orientations of collagen fibers and the BAp c-axis. (F) Young’s modulus along the bone longitudinal axis at position 5. **: P<0.01. X symbols in box plots show outliers. (G) Single regression analysis of Young’s modulus to BMD and each degree of the preferential alignment of collagen fibers and the BAp c-axis in the bone longitudinal direction at position 5. wt: blue dots, Ocn–/–: red dots. n = 6. (TIF) (TIF) [file pgen.1008586.s005.tif]

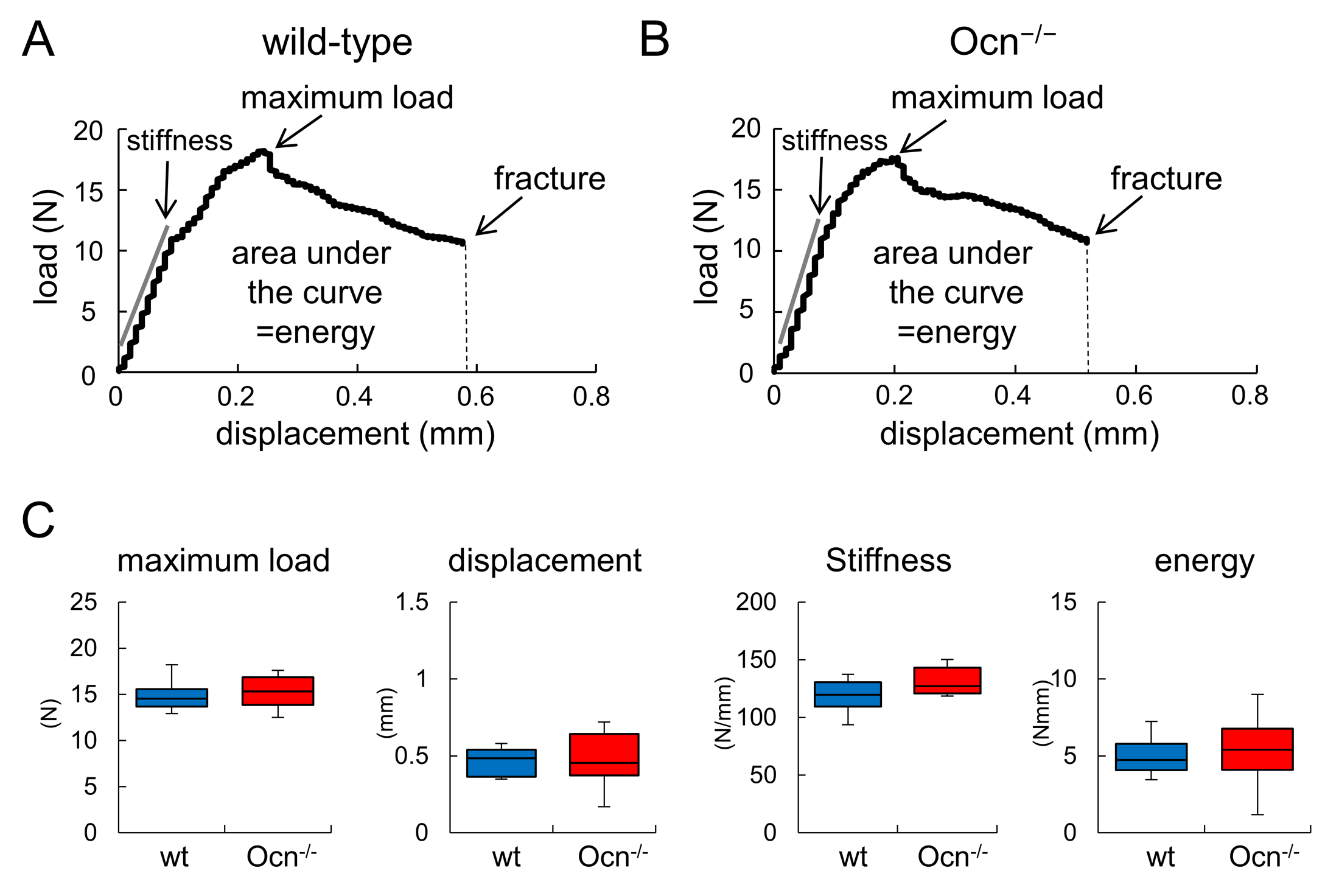

Supplement: S6 Fig — Representative load-displacement curves for male wild-type (A) and Ocn–/–(B) mice at 6 months of age, in which maximum load, displacement, stiffness (the slope of the linear part of the load), and energy to failure (area under the load-displacement curve) were obtained. C, Maximum load, displacement, stiffness, and energy in wild-type (n = 8) and Ocn–/–(n = 6) mice. The experiments were performed as previously described (J Bone Miner Res 2016; 31: 1366–1380.). (TIF) (TIF) [file pgen.1008586.s006.tif]

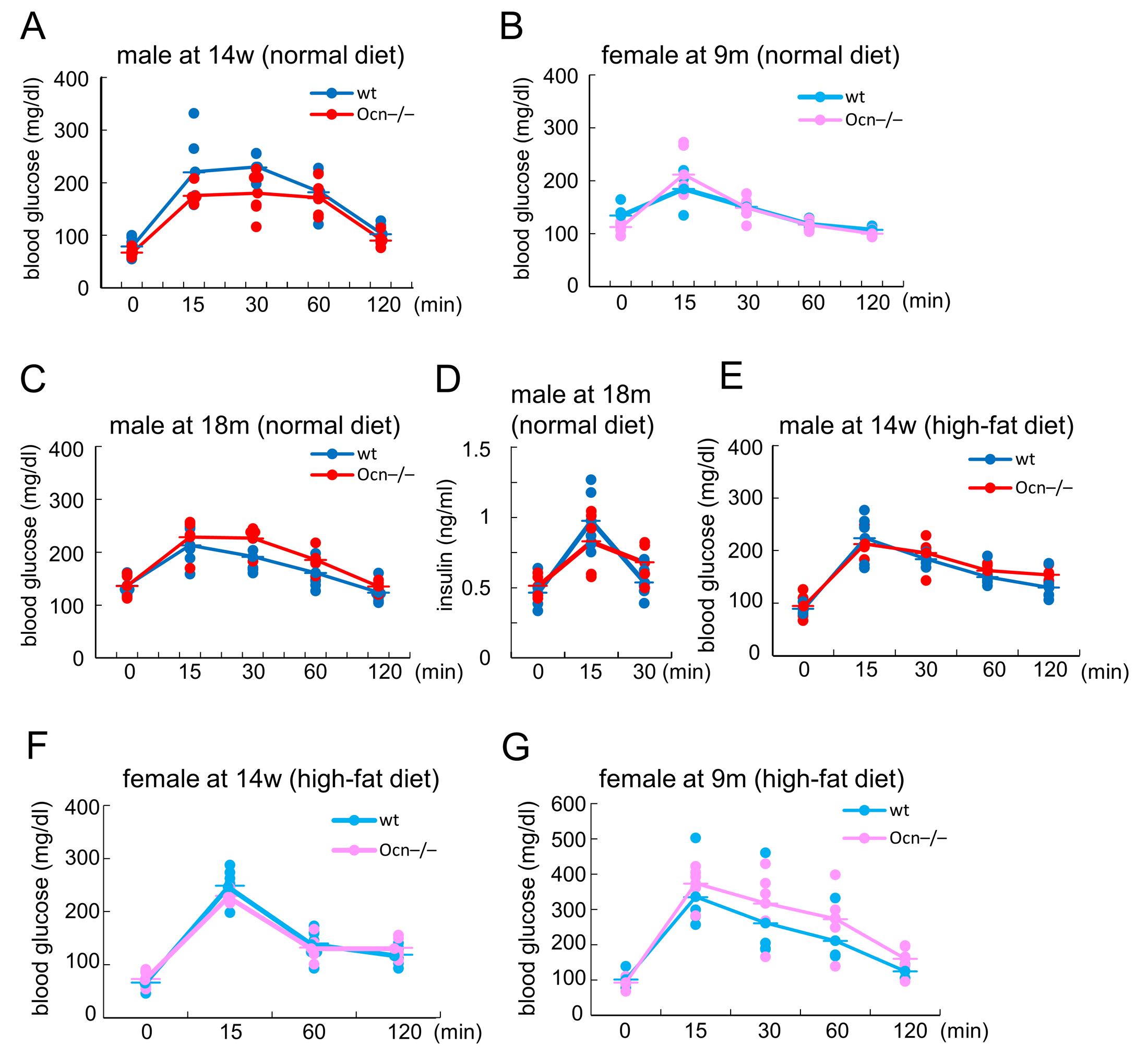

Supplement: S7 Fig — Glucose (1 g/kg body weight) was injected intraperitoneally in GTTs. (A) Glucose levels in male mice at 14 weeks of age fed a normal diet. wt: n = 5, Ocn–/–: n = 6. (B) Glucose levels in female mice at 9 months of age fed a normal diet. wt: n = 4, Ocn–/–: n = 6. (C and D) Glucose (C) and insulin (D) levels in male mice at 18 months of age fed a normal diet. wt: n = 7, Ocn–/–: n = 5. (E) Glucose levels in male mice at 14 weeks of age fed a high-fat diet for 5 weeks. wt: n = 5, Ocn–/–: n = 4. (F) Glucose levels in female mice at 14 weeks of age fed a high-fat diet for 5 weeks. wt: n = 7, Ocn–/–: n = 5. (G) Glucose levels in female mice at 9 months of age fed a high-fat diet for 11 weeks. wt: n = 4, Ocn–/–: n = 5. (TIF) (TIF) [file pgen.1008586.s007.tif]

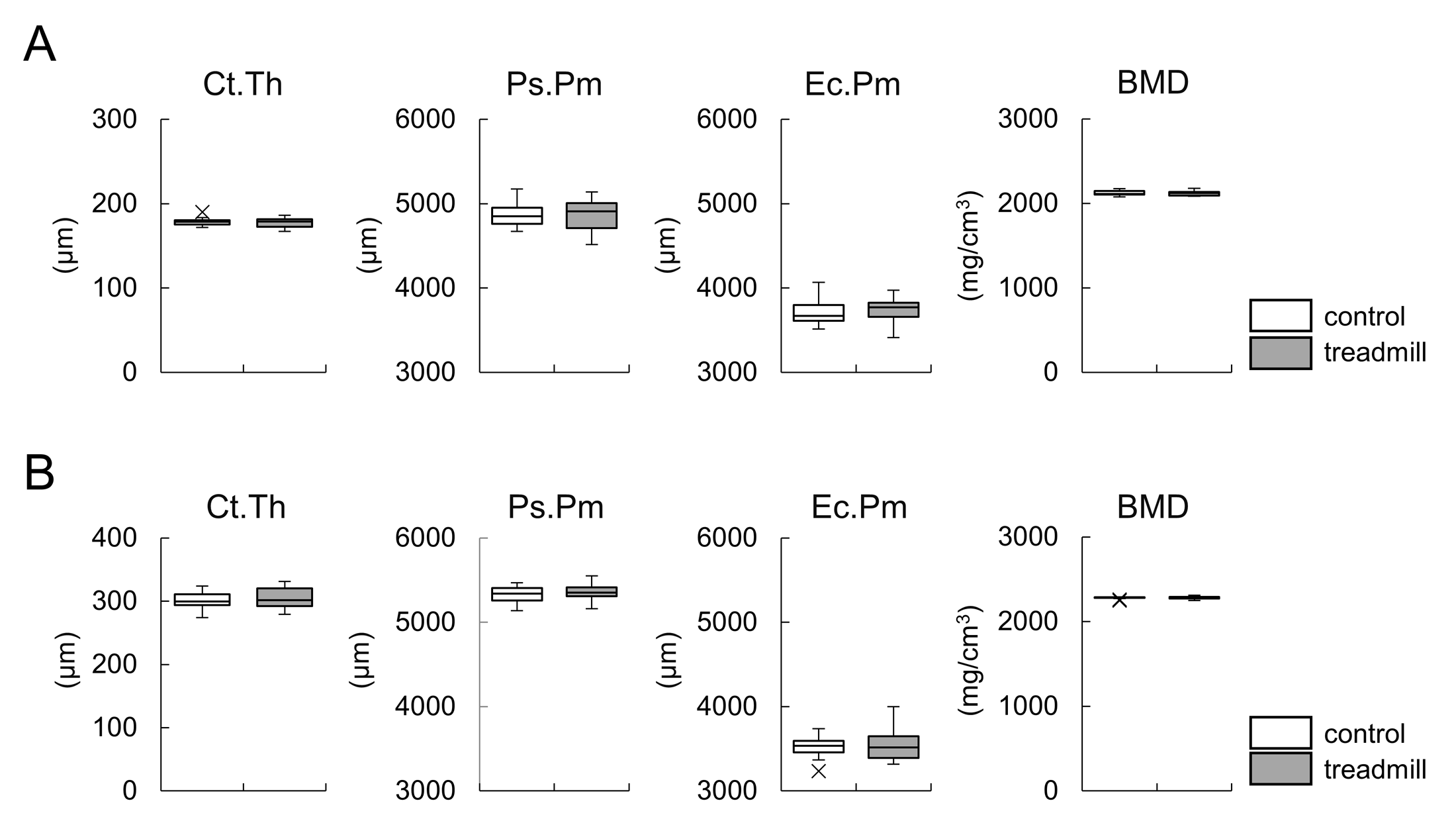

Supplement: S8 Fig — Male wild-type (A) and KK/TaJcl (B) mice with or without exercise on a treadmill for 7 weeks were analyzed by μ-CT at 4 months of age. Cortical thickness (Ct. Th), the periosteal perimeter (Ps.Pm), endocortical perimeter (Ec.Pm), and BMD are shown. Control: n = 13, treadmill: n = 13 in wild-type mice. Control: n = 15, treadmill: n = 14 in KK/TaJcl mice. X symbols in box plots show outliers. (TIF) (TIF) [file pgen.1008586.s008.tif]

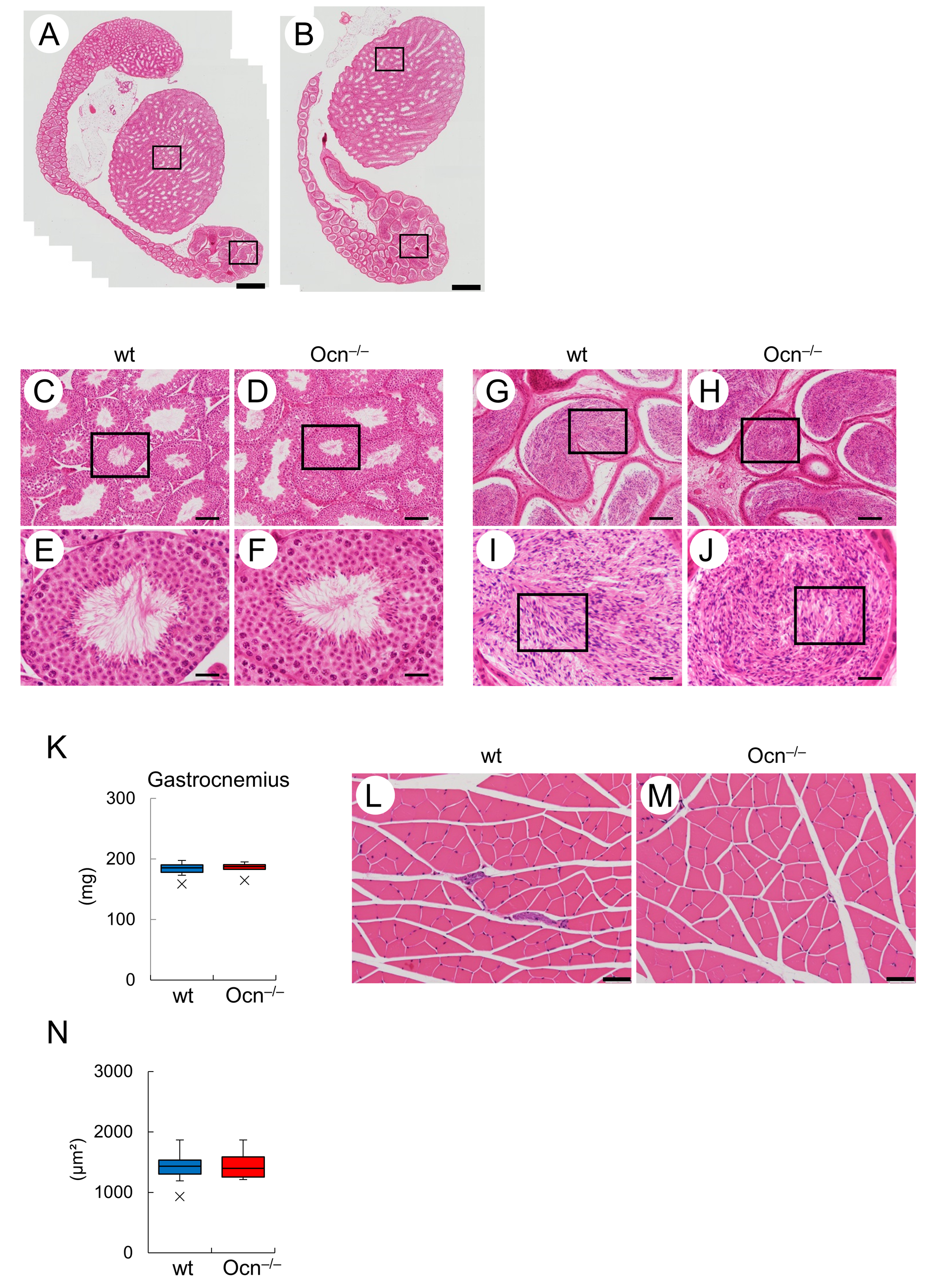

Supplement: S9 Fig — (A-J) Histological sections of testis and epididymis at 4 months of age stained with H-E. Testis and epididymis at low magnification (A, B), seminiferous tubules (C-F), and cauda epididymis (G-J) are shown. Boxed regions in C, D, G, and H are magnified in E, F, I, and J, respectively. Boxed regions in I and J are magnified in Fig 11D. Bars: 1mm (A, B); 100 μm (C, D, G, H); and 20 μm (E, F, I, J). (K-N) Muscle weights (K), H-E stained sections (L, M), and the average areas of myofibers (N) in gastrocnemius muscle in male wild-type (n = 8) and Ocn–/–(n = 7) mice at 9 months of age. Bars: 50 μm. X symbols in box plots show outliers. (TIF) (TIF) [file pgen.1008586.s009.tif]
