# Supplementary material for: Osteocalcin is necessary for the alignment of apatite crystallites, but not glucose metabolism, testosterone synthesis, or muscle mass
Source: PLoS Genet. 2020 May 28;16(5):e1008586. doi: 10.1371/journal.pgen.1008586 (PMC7255595; doi:10.1371/journal.pgen.1008586)
Supplement: Attachment — Submitted filename: PGENETICS-D-19-01071_R1.pdf [file pgen.1008586.s014.pdf]

PLOS Genetics

Osteocalcin is necessary for the alignment of apatite crystallites, but not glucose metabolism, testosterone synthesis, or muscle mass

--Manuscript Draft--

**Manuscript Number:**	PGENETICS-D-19-01071R1
**Full Title:**	Osteocalcin is necessary for the alignment of apatite crystallites, but not glucose metabolism, testosterone synthesis, or muscle mass
**Short Title:**	Osteocalcin is necessary for the alignment of apatite crystallites in bone but does not work as a hormone
**Article Type:**	Research Article
**Section/Category:**	General
**Keywords:**	osteocalcin; apatite crystallite; glucose metabolism; testosterone; muscle
**Abstract:**	The strength of bone is dependent on bone quantity and quality. Osteocalcin (Ocn), which is specifically produced by osteoblasts and is the most abundant non-collagenous protein in bone, has been shown to inhibit bone formation. It also functions as a hormone that regulates insulin secretion in the pancreas, testosterone synthesis in the testes, and muscle mass. We generated Ocn-deficient (Ocn –/–) mice by deleting Bglap and Bglap2 . However, an analysis of Ocn –/– mice revealed that Ocn was not involved in the regulation of bone quantity, glucose metabolism, testosterone synthesis, or muscle mass. Although the orientation degree of collagen fibrils and size of biological apatite (BAp) crystallites in the c -axis were normal in Ocn –/– bone, the crystallographic orientation of the BAp c -axis, which is normally parallel to collagen fibrils, was severely disrupted, resulting in reduced bone strength. These results demonstrate that Ocn is required for maintaining bone quality and strength by adjusting the alignment of BAp crystallites parallel to collagen fibrils.
**Additional Information:**	
**Question**	**Response**
**Financial Disclosure**<br><br>Enter a financial disclosure statement that describes the sources of funding for the work included in this submission. Review the submission guidelines for detailed requirements. View published research articles from *PLOS Genetics* for specific examples.<br><br>This statement is required for submission and **will appear in the published article** if the submission is accepted. Please make sure it is accurate.	This work was supported by grants from the Japanese Ministry of Education, Culture, Sports, Science and Technology to TK (Grant number: 18H05283) and to TM (18K09070). The funders had no role in study design, data collection and analysis, decision to publish, or preparation of the manuscript.

Powered by Editorial Manager® and ProduXion Manager® from Aries Systems Corporation

**Unfunded studies**<br><br>Enter: *The author(s) received no specific funding for this work.*<br><br>**Funded studies**<br><br>Enter a statement with the following details:<br>• Initials of the authors who received each award<br>• Grant numbers awarded to each author<br>• The full name of each funder<br>• URL of each funder website<br>• Did the sponsors or funders play any role in the study design, data collection and analysis, decision to publish, or preparation of the manuscript?<br>• **NO** - Include this sentence at the end of your statement: *The funders had no role in study design, data collection and analysis, decision to publish, or preparation of the manuscript.*<br>• **YES** - Specify the role(s) played.<br><br>* typeset	
**Competing Interests**<br><br>Use the instructions below to enter a competing interest statement for this submission. On behalf of all authors, disclose any competing interests that could be perceived to bias this work—acknowledging all financial support and any other relevant financial or non-financial competing interests.<br><br>This statement **will appear in the published article** if the submission is accepted. Please make sure it is accurate. View published research articles from *PLOS Genetics* for specific examples.	The authors have declared that no competing interests exist.

Powered by Editorial Manager® and ProduXion Manager® from Aries Systems Corporation

**NO authors have competing interests**<br><br>Enter: *The authors have declared that no competing interests exist.*<br><br>**Authors with competing interests**<br><br>Enter competing interest details beginning with this statement:<br><br>*I have read the journal's policy and the authors of this manuscript have the following competing interests: [insert competing interests here]*<br><br>* typeset	
**Data Availability**<br><br>Authors are required to make all data underlying the findings described fully available, without restriction, and from the time of publication. PLOS allows rare exceptions to address legal and ethical concerns. See the PLOS Data Policy and FAQ for detailed information.<br><br>A Data Availability Statement describing where the data can be found is required at submission. Your answers to this question constitute the Data Availability Statement and will be published in the article, if accepted.<br><br>**Important:** Stating 'data available on request from the author' is not sufficient. If your data are only available upon request, select 'No' for the first question and explain your exceptional situation in the text box.<br><br>Do the authors confirm that all data underlying the findings described in their manuscript are fully available without restriction?	No - some restrictions will apply

**Describe where the data may be found in full sentences. If you are copying our sample text, replace any instances of XXX with the appropriate details.**<br><br>• If the data are **held or will be held in a public repository**, include URLs, accession numbers or DOIs. If this information will only be available after acceptance, indicate this by ticking the box below. For example: *All XXX files are available from the XXX database (accession number(s) XXX, XXX.).*<br>• If the data are all contained **within the manuscript and/or Supporting Information files**, enter the following: *All relevant data are within the manuscript and its Supporting Information files.*<br>• If neither of these applies but you are able to provide **details of access elsewhere**, with or without limitations, please do so. For example:<br><br>*Data cannot be shared publicly because of [XXX]. Data are available from the XXX Institutional Data Access / Ethics Committee (contact via XXX) for researchers who meet the criteria for access to confidential data.*<br><br>*The data underlying the results presented in the study are available from (include the name of the third party and contact information or URL).*<br>• This text is appropriate if the data are owned by a third party and authors do not have permission to share the data.<br><br>* typeset	All relevant data are within the manuscript and its Supporting Information files.
Additional data availability information:	Tick here if the URLs/accession numbers/DOIs will be available only after acceptance of the manuscript for publication so that we can ensure their inclusion before publication.

Response to the reviewers

Reviewer #1: During the last twenty three years, the bone and mineral metabolism research community (and the NIH institutes and numerous other national and international funding agencies that support it) have devoted a great deal of intellectual energy and resources on some very provocative, albeit controversial, ideas about osteocalcin (OCN) – a 46 amino-acid protein that is made in bone and binds avidly to the hydroxyapatite mineral. OCN is produced and secreted almost exclusively by osteoblasts, terminally differentiated cells responsible for the synthesis and mineralization of the bone matrix during the development of the skeleton and its periodic regeneration throughout life. Osteoblasts are short-lived cells originating from mesenchymal progenitors that are replaced depending on the demand for bone formation in a particular location and time. OCN secreted by osteoblasts contains three γ-carboxyglutamic acid residues that are removed by the acidic pH created during the resorption of bone by osteoclasts.

According to ideas originated and propagated by Gerard Karsenty and co-workers, osteoblasts comprise an endocrine organ and decarboxylated OCN is a hormone. The circulating levels of this "new hormone" are, therefore, dependent on the rate of bone turnover, a.k.a. remodeling. Specifically, Karsenty and collaborators have claimed in a series of high profile publications over a period of several years that OCN acts on multiple organs and tissues including bone, pancreas, liver, adipose, muscles, testicles, and the brain to regulates functions ranging from bone mass accumulation, to body weight, adipocity, glucose metabolism, energy utilization, male fertility, mentation, and behavior. Notably, all these claims have been based on the results of studies of a single mouse model with genetic deletion of OCN, generated in the Karsenty laboratory over 23 years ago, but never made available to other investigators seeking to reproduce the results.

However, unlike hormones produced and released by dedicated cells in response to external stimuli, the number of osteoblasts and thereby the circulating levels of osteocalcin inexorably change throughout life as a result of physiologic or

pathologic changes of bone itself that can be acute or chronic, systemic or localized, and reversible or irreversible. Examples are skeletal development, growth, adaptation of the skeleton to mechanical forces, fracture healing, changing calcium needs, stress, menstrual cycle, pregnancy, lactation, menopause, aging, hyper- or hypo-parathyroidism, hyperthyroidism, hypercortisolemia, Paget's disease, bone tumors, etc. Furthermore, medications – approved after extensive trials with thousands of subjects and used by millions for the treatment of osteoporosis – decrease or increase serum osteocalcin levels without any effect on glucose homeostasis, testosterone production, muscles, or behavior. The glaring shortcomings and incongruence of the Karsenty ideas with physiology, pathophysiology, clinical medicine, and pharmacology notwithstanding, the concerns with the hypothesis that bone and osteocalcin regulate many other tissues have progressively intensified over the years by the fact that other groups have failed to reproduce the observations of Karsenty and colleagues in different mice or in a rat model with OCN deletion. Still major journal review articles and prestigious teaching textbooks have continued until now to publish the Karsenty claims as proven biologic facts.

In the article by Moriishi and co-workers, the authors have generated their own OCN deficient mouse, using gene targeting in embryonic stem cells and microinjection into C57BL/6 blastocysts. Using this model they investigated the role of OCN on bone formation and mineralization, as well as glucose metabolism, testosterone production and muscle mass. They report that, in contrast to the results reported by Karsenty and colleagues, OCN plays no role in bone formation (or resorption) and the accumulation on bone mass in the estrogen sufficient or the estrogen deficient state. Instead, OCN is indispensable for the alignment of biological apatite crystallites parallel to collagen fibers. Loss of OCN function had no effect on collagen orientation, which remained normal. Bone strength, however, was decreased in the OCN deficient mice indicating that alignment of crystallites with collagen fibers is one of the elusive determinants of bone quality that together with bone mass determines the ability of bone to resist fractures. Additionally, Moriishi and colleagues show that OCN plays no role in the exercise-induced bone formation, glucose metabolism, the improvement of glucose metabolism caused by

exercise, testosterone synthesis and spermatogenesis, or muscle mass.

This is an exceptionally thorough and careful investigation of the effects of OCN on bone as well as the earlier suggestions the OCN acts as hormone to regulate glucose metabolism, fertility, and muscle mass. The methods of analysis are flawless, the results (with the exception of the OVX experiment) are unambiguous, and the conclusions are fully justified and convincing. The writing of the manuscript, however, is suboptimal. There are unnecessary repetitions, stylistic issues and problems with the grammar that need to be addressed so as to improve the readability of this, otherwise, very impressive work.

Specific comments

1. Legend of Figure 1 H and I: In this and the legend of several other figures the X symbol above or below the box plots, presumably outliers, has not been defined.

We indicated that the X symbols in box plots show outliers in the figure legends, and we described the definition of outliers in the last part of Method section on pages 22-23.

2. Supplemental figure 2D: Cortical thickness and remodeling markers were not affected by OVX in the wild type control mice of this experiment (OVX at 5 weeks and sacrifice at 11 weeks). These results are in contrast to the well documented effect of OVX in adult mice that have already reached peak bone mass (4-5 months of age). The authors need to address this discrepancy and acknowledge the likelihood that during the experimental estrogen deficiency in their mice the femurs continued to grow, thereby confounding a meaningful interpretation.

C57BL/6 mouse line is relatively insensitive to OVX, and cortical thickness does not reduce at 6 weeks after OVX in mice ovariectomized at 12 weeks of age and sacrificed at 18 weeks of age (ref. 1). We mentioned it with the reference on page 7. We

previously showed that the urinary deoxypyridinoline apparently increase at 1 week after OVX in mice ovariectomized at 4 months of age (ref. 2), and we observed that the levels of TRAP5b were similar between sham and OVX mice at 6 weeks after OVX in C57BL/6 mice ovariectomized at 13 weeks of age in another project (unpublished data). Two Japanese papers also reported that urinary NTX transiently increases at 1-3 weeks after OVX in rats ovariectomized at 12 or 19 weeks of age and returns to a normal level at 18 or 25 weeks of age, respectively. Therefore, we think that serum resorption markers increase at 1-3 weeks after OVX and returns to the normal levels at 6 weeks after OVX in both adult and young mice. In the supplementary figure 2E, we wanted to show that TRAP5b returns to a normal level in both wild type and $Ocn^{-/-}$ mice at 6 weeks after OVX. Since we did not examine TRAP5b at 1-3 weeks after OVX in this experiment, however, we deleted the supplementary figure 2E.

Ref. 1: Klinck, J. & Boyd, S. K. The magnitude and rate of bone loss in ovariectomized mice differs among inbred strains as determined by longitudinal in vivo micro-computed tomography. *Calcified tissue international* **83**, 70-79, 2008.

Ref 2: Maruyama Z, Yoshida CA, Furuichi T, Amizuka N, Ito M, Fukuyama R, Miyazaki T, Kitaura H, Nakamura K, Fujita T, Kanatani N, Moriishi T, Yamana K, Liu W, Kawaguchi H, Nakamura K, Komori T: Runx2 determines bone maturity and turnover rate in postnatal bone development and is involved in bone loss in estrogen deficiency. *Dev Dyn*. 236(7):1876-1890, 2007.

Grammatical and stylistic suggestions:

1. The title should be shortened as follows: Osteocalcin is necessary for the alignment of apatite crystallites, but not glucose metabolism, testosterone synthesis, or muscle mass.

We agree to shorten the title according to your suggestion "Osteocalcin is necessary for the alignment of apatite crystallites, but not glucose metabolism, testosterone synthesis, or muscle mass".

2. Osteocalcin should be abbreviated throughout the text as OCN. The use of articles, like a, as in “a Real-time PCR analysis” or “ A bone histomorphometric analysis” is unnecessary.

We abbreviated osteocalcin as Ocn in the whole text. The articles were deleted.

3. Abstract: I took the liberty to suggest the following small edits for the sake of clarity and brevity: Osteocalcin (OCN) which is specifically produced by osteoblasts and is the most abundant non-collagenous protein in bone. It has been shown suggested previously that OCN shown to inhibits bone formation. It also and functions as a hormone that to regulates insulin secretion in the pancreas, testosterone synthesis in the testes, and muscle mass. We have generated a strain of OCN deficient mice using gene targeting in embryonic stem cells and microinjection into C57BL/6 blastocysts. However, an analysis of Ocn−/− mice revealed Loss of OCN function had no effect on the regulation of bone quantity, glucose metabolism, testosterone synthesis, or muscle mass in these mice. OCN deletion, however, disrupted the alignment of biological apatite crystallites parallel to collagen fibers and caused loss of strength, indicating that alignment of crystallites with collagen fibers is one of the determinants of bone quality. that together with bone mass determines the ability of bone to resist fractures. Although the orientation degree of collagen fibrils and size of biological apatite (BAp) crystallites in the c-axis were normal in Ocn−/− bone, the crystallographic orientation of the BAp c-axis, which is normally parallel to collagen fibrils, was severely disrupted, resulting in reduced bone strength. These results demonstrate that osteocalcin is required for maintaining bone quality and strength by adjusting the alignment of BAp crystallites parallel to collagen fibrils.

Thank you for the suggestion.

We changed “The fracture risk of bone” to “The strength of bone”, and added a sentence “We generated Ocn-deficient ($Ocn^{-/-}$) mice by deleting *Bglap* and *Bglap2*”.

4. Author Summary: Here again, I’ve taken the liberty to suggest the following small edits. The strength of bone is dependent on both bone quantity and quality. Osteocalcin (OCN) is the most abundant non-collagenous protein in bone. It has been suggested before that OCN inhibits bone formation, and serum osteocalcin has been shown to and works as a hormone that to regulates glucose metabolism, testosterone synthesis, and muscle mass. We have generated osteocalcin-deficient mice, and found that osteocalcin is not involved in the regulation of bone quantity. The major factors that influence bone quality are the alignment of collagen fibers and mineralization. Collagen fibers align parallelly to the longitudinal axis of bone and mineralization occurs by the growth of apatite crystallites parallel to the collagen. We showed that In our osteocalcin-deficient mice collagen fibers aligned normally but apatite crystallites aligned randomly against collagen, resulting in reduced bone strength. Further, we found that all of the data in found that glucose metabolism, testosterone synthesis, and muscle mass are normal in osteocalcin-deficient these mice. We demonstrated conclude from this work that OCN aligns apatite crystallites parallelly to collagen fibers and thereby it maintains bone quality and strength. but that sSerum osteocalcin, however, does not work as a hormone and does not affect regulates glucose metabolism, testosterone synthesis, and muscle mass.

Thank you for the suggestion.

5. Introduction, pages 3-7: This entire section is long, verbose, and unnecessarily detailed. It should be shortened and simplified, for example by using review articles to summarize the earlier findings/claims about OCN.

The introduction was shortened and simplified.

6. Introduction, 1st paragraph: Fracture risk depends on many factors besides the quantity and quality of bone, for example falls. Gla should be defined the first time it is mentioned on line 7, but in any case the use of the terms Gla and Glu is confusing and unhelpful. Carboxylated and de-carboxylated or un-corboxylated OCN are much simpler and easier terms for the reader to follow. Carboxylated OCN has (instead of exhibits) high affinity for (instead of to) Ca.

“Fracture risk” was changed to “The strength of bone” on page 3. We changed Gla osteocalcin to carboxylated Ocn, and changed Glu osteocalcin to uncarboxylated Ocn. We also changed “Gla osteocalcin exhibits high affinity to Ca” to “carboxylated Ocn has high affinity for Ca” (page 3).

7. Introduction, last paragraph: this is an unnecessary repetition of the abstract and summary.

We deleted most of the last paragraph to avoid a repetition.

8. Page 17, last two lines to end of this paragraph on page 18: the way this paragraph is written it implies that the earlier claims are factual. This is confusing. Moreover, several of the thoughts discussed here are repetition of earlier sections.

The following sentences were deleted:

Glu osteocalcin functions as a hormone, which is delivered from bone and regulates glucose metabolism, testosterone synthesis, and muscle mass [30,47,59,75].

However, these were normal in our Ocn$^{-/-}$ mice, irrespective of the deletion of both *Bglap* and *Bglap2* as the previous Ocn$^{-/-}$ mice. There is a similar and unexplainable discrepancy in the phenotypes of the deletion of *Gprc6a*, which has been shown to encode an osteocalcin receptor that is required for the functions of osteocalcin as a hormone [47,54,56]. *Gprc6a*$^{-/-}$ mice with the deletion of exon 2 showed impaired glucose metabolism and testosterone synthesis and reduced BMD, whereas *Gprc6a*$^{-/-}$ mice with the deletion of exon 6 or the full locus were normal in glucose metabolism, fertility, and BMD [49-53].

Reviewer #2: The authors constructed mice knocked out for the two mouse osteocalcin genes and the DNA between the osteocalcin genes in a manner that appears similar to the strategy initially used by Ducy et al. they show that bone mass, bone histomorphometry, serum markers of bone formation and resorption are similar between WT and KO mice. Using EM analysis of antibody treated sections they show that osteocalcin is located in both the intrafibrillar and interfibrillar regions of collagen in WT mice and not detected in the KO. Raman microspectroscopy also revealed no differences in the collagen and mineral chemical indices. However, the c-axis orientation degree of bone apatite was disrupted in the KO mice compared to WT; this correlated with a lower Yong's modulus in single and multiple regression analysis, after nanoindentation testing. Three point bending analysis was normal in the KO mice. Glucose and insulin tolerance tests were normal in the KO mice, as was their weight and blood glucose. In normal mice exercise improves glucose metabolism and bone formation. Male KO mice had normal sperm and testosterone levels and were fertile. Indices of muscle mass were normal in the KO. Thus, the authors fail to confirm several of the previous findings from the Karsenty group's studies of similar mice.

1. The wording of the conclusion at the end of page 18 is too sweeping. The authors show that removal of the osteocalcin gene shows that osteocalcin is not needed for normal bone formation, glucose metabolism, or male testosterone production. This is an important and novel series of observations. But, they did not test and cannot comment on the ability of osteocalcin to regulate bone formation, glucose

metabolism, testosterone synthesis or the maintenance of muscle mass. It is always possible that other redundant hormones and paracrine factors can compensate for the absence of osteocalcin in the KO mice. So, the authors need to be more constrained in their summary claim.

Thank you for the suggestion.

We changed the conclusion to describe our findings more precisely as follows:

In conclusion, Ocn is not necessary for the regulation of bone formation or resorption, but is essential for the alignment of the BAp *c*-axis parallel to collagen fibrils and is required to maintain bone strength. However, Ocn does not work as a hormone that regulates glucose metabolism, testosterone synthesis, and muscle mass.

2. Figure 9G. It is not clear what statistical analysis led to the conclusion that the blue dots, which appear to be higher than the red dots, are not statistically different.

Statistical analysis was performed by one-way ANOVA and the Tukey-Kramer post hoc test, and there were no statistical differences in Fig. 9G.

3. Figure 10. There's no mention I can find of what the asterixes in this figure signify.

We indicated the significance "*: $P<0.05$, **: $P<0.01$, ***: $P<0.001$" in the legend.

4. It might be useful for the authors to comment on why the results from their studies differ from those of the Karsenty group. For example, are the knockouts exactly the same? I can't tell from a simple comparison of the papers. If some sequence adjacent to the coding regions for osteocalcin have effects on another gene, for example, then perhaps that could explain differences. Or maybe different versions of C57B mice could make a difference.

We described the difference of the two $Ocn^{-/-}$ mouse lines on page 16 as follows:

The phenotypes of increased bone mass, impaired glucose metabolism, and reduced testosterone synthesis and muscle mass in the previous $Ocn^{-/-}$ mice were not observed in our $Ocn^{-/-}$ mice, although both *Bglap* and *Bglap2* were deleted and *Bglap3*, which is expressed in non-osteoid tissues with unknown function, was intact in both $Ocn^{-/-}$ mouse lines. The only difference between the two mouse lines is the genetic background. Our $Ocn^{-/-}$ mice were backcrossed to C57BL/6N more than 8 times before analysis, while the previous $Ocn^{-/-}$ mice were analyzed in the mixed background of C57BL/6J and 129/SV.

Reviewer #3: This is a Herculean manuscript that describes the KO phenotype of a Blglap/Bglap2 KO mouse. The Ocn-/- mouse phenotype is of great interest due to a series of high profile publications on a previous Ocn-/- mouse line, which report a number of key roles for Ocn in regulating fertility and metabolism.

The authors perform a number of studies in great detail, including both male and female mice of different ages in studies of bone structure, glucose metabolism, and fertility. The authors comprehensively demonstrate a lack of phenotype for their Ocn-/- mouse in the above endpoints – bar an effect on alignment of apatite crystals and sequelae.

Overall, I comment the authors and have few, if any, comments on the studies

performed. The results seem very clear. Find below some considerations and minor questions:

1. In general the statistical analyses are not well described; please clarify the nature of the ANOVA performed (in some cases it could be one-, two-, or three-way ANOVA), and give the overall P value for the genotype effect (which, looking at the graphs, would be absent for most of the figures). Also, I presume that the “x” symbols on the graphs are either outliers, or limits of the data range beyond which would be outliers? Please clarify – and if outliers, describe the method used to exclude.

Statistical analyses of more than three groups were performed by one-way ANOVA and the Tukey-Kramer post hoc test. The P values of less than 0.05 were considered as statistical significance. The P value of less than 0.05 is shown as *, that of less than 0.01 is shown as **, and that of less than 0.001 is shown as ***. No asterisk means that there is no statistical significance. We hesitated to show non-significant P values (more than 0.05) in all graphs, because our figures contain many graphs, each graph contains many comparisons, and the addition of non-significant P values makes the graphs too busy. We used the box plots to visually show the distribution of numerical data and skewness by displaying the data quartiles and averages. X symbols on the graphs mean outliers. The outliers were defined as data points located outside 1.5 times the interquartile range (IQR, shown as a box) above the upper quartile or those located outside 1.5 times the IQR below the lower quartile. We indicated that the X symbols in box plots show outliers in the figure legends, and we described the definition of outliers in the last part of Method section on pages 22-23.

2. Fig 4 – please clarify how these images were selected for the figure (as relatively few Ocn immunoreactive dots are visible in field).

Completely mineralized regions with some distance from osteoid were selected. Ocn immunoreactivity has been shown to be severely reduced without addition of 2mM Ca^{2+} to the Ocn antibody solution in immunogold electron microscopy (ref). We performed it without the addition of 2mM Ca^{2+}. It may be a reason why the number of Ocn immunoreactive dots was relatively low in our analysis.

ref: Chen, L., Jacquet, R., Lowder, E. & Landis, W. J. Refinement of collagen-mineral interaction: a possible role for osteocalcin in apatite crystal nucleation, growth and development. *Bone* **71**, 7-16, (2015).

3. Please comment on the potential role (or lack thereof) of Bglap3 – and any differences, or not, with respect to previous Ocn-/- mice lines?

We commented it on page 16 as follows:

The phenotypes of increased bone mass, impaired glucose metabolism, and reduced testosterone synthesis and muscle mass in the previous $Ocn^{-/-}$ mice were not observed in our $Ocn^{-/-}$ mice, although both *Bglap* and *Bglap2* were deleted and *Bglap3*, which is expressed in non-osteoid tissues with unknown function, was intact in both $Ocn^{-/-}$ mouse lines. The only difference between the two mouse lines is the genetic background. Our $Ocn^{-/-}$ mice were backcrossed to C57BL/6N more than 8 times before analysis, while the previous $Ocn^{-/-}$ mice were analyzed in the mixed background of C57BL/6J and 129/SV.

4. Fig 7 – correlation of YM and Bap c-axis orientation – whilst there is a significant correltion, the points are rather clustered at each end of the regression – so I would interpret with caution.

All wild-type mice showed high degree of BAp c-axis orientation (parallel to longitudinal direction) and high Young's modulus, and all Ocn-/- mice showed low degree of BAp c-axis orientation (random orientation) and low Young's modulus. Therefore, there was no mouse to show the intermediate phenotype.

5. Given the controversy in the field, it might be useful if the authors make a written statement that they will share their new mouse line with other researchers?

We described it on page 16 as follows:

Since the difference of the genetic background is unlikely to explain all controversial phenotypes, our $Ocn^{-/-}$ mouse line will be shared with other researchers to resolve the controversies.

Have all data underlying the figures and results presented in the manuscript been provided?

Large-scale datasets should be made available via a public repository as described in the *PLOS Genetics* data availability policy, and numerical data that underlies graphs or summary statistics should be provided in spreadsheet form as supporting information.

Reviewer #1: Yes

Reviewer #2: Yes

Reviewer #3: Yes

PLOS authors have the option to publish the peer review history of their article (what does this mean?). If published, this will include your full peer review and any attached files.

If you choose "no", your identity will remain anonymous but your review may still be made public.

Do you want your identity to be public for this peer review? For information about this choice, including consent withdrawal, please see our Privacy Policy.

Reviewer #1: Yes: Stavros C. Manolagas, MD, PhD

Reviewer #2: No

Reviewer #3: No

Manuscript Click here to access/download;Manuscript;osteocalcin 10_13_19.docx

Osteocalcin is necessary for the alignment of apatite crystallites, but not glucose metabolism, testosterone synthesis, or muscle mass

Takeshi Moriishi[1], Ryosuke Ozasa[2], Takuya Ishimoto[2], Takayoshi Nakano[2], Tomoka Hasegawa[3], Toshihiro Miyazaki[1], Wenguang Liu[4,6], Ryo Fukuyama[5], Yuying Wang[4,7], Hisato Komori[4], Xin Qin[4], Norio Amizuka[3], and Toshihisa Komori[4]*

[1]Department of Cell Biology and [4]Basic and Translational Research Center for Hard Tissue Disease, Nagasaki University Graduate School of Biomedical Sciences, Nagasaki 852-8588, Japan

[2]Division of Materials and Manufacturing Science, Graduate School of Engineering, Osaka University, 2-1, Yamada-Oka, Suita, Osaka 565-0871, Japan

[3]Department of Developmental Biology of Hard Tissue, Division of Oral Health Science, Graduate School of Dental Medicine, Hokkaido University, Sapporo 060-8586, Japan

[5]Laboratory of Pharmacology, Hiroshima International University, Kure 737-0112, Japan

[6]present address; Institute of Genetics and Cytology, Northeast Normal University, Changchun 130024, China.

[7]present address; College of Life and Environmental Science, Hangzhou Normal University, Hangzhou, 310036, China.

*Corresponding author:

Basic and Translational Research Center for Hard Tissue Disease, Nagasaki University Graduate School of Biomedical Sciences, 1-7-1 Sakamoto, Nagasaki 852-8588, Japan

TEL: +81-95-819-7637; FAX: +81-95-819-7638

E-mail: komorit@nagasaki-u.ac.jp

Key words: osteocalcin, apatite crystallite, glucose metabolism, testosterone, muscle

Abstract

The strength of bone is dependent on bone quantity and quality. Osteocalcin (Ocn), which is specifically produced by osteoblasts and is the most abundant non-collagenous protein in bone, has been shown to inhibit bone formation. It also functions as a hormone that regulates insulin secretion in the pancreas, testosterone synthesis in the testes, and muscle mass. We generated Ocn-deficient (Ocn$^{-/-}$) mice by deleting *Bglap* and *Bglap2*. However, an analysis of Ocn$^{-/-}$ mice revealed that Ocn was not involved in the regulation of bone quantity, glucose metabolism, testosterone synthesis, or muscle mass. Although the orientation degree of collagen fibrils and size of biological apatite (BAp) crystallites in the *c*-axis were normal in Ocn$^{-/-}$ bone, the crystallographic orientation of the BAp *c*-axis, which is normally parallel to collagen fibrils, was severely disrupted, resulting in reduced bone strength. These results demonstrate that Ocn is required for maintaining bone quality and strength by adjusting the alignment of BAp crystallites parallel to collagen fibrils.

Author Summary

The strength of bone is dependent on bone quantity and quality. Osteocalcin (Ocn) is the most abundant non-collagenous protein in bone. Ocn has been shown to

inhibit bone formation, and serum osteocalcin has been shown to work as a hormone that regulates glucose metabolism, testosterone synthesis, and muscle mass. We generated Ocn-deficient mice, and found that Ocn is not involved in the regulation of bone quantity. The major factors that influence bone quality are the alignment of collagen fibers and mineralization. Collagen fibers align parallelly to the longitudinal axis of bone, and mineralization occurs by the growth of apatite crystallites parallel to the collagen. We showed that Ocn-deficient mouse is the first case that collagen fibers align normally, but apatite crystallites align randomly against collagen, resulting in reduced bone strength. Further, we found that all of the data in glucose metabolism, testosterone synthesis, and muscle mass are normal in Ocn-deficient mice. We demonstrated that Ocn aligns apatite crystallites parallelly to collagen fibers and maintains bone quality and strength, but that serum Ocn does not work as a hormone that regulates glucose metabolism, testosterone synthesis, and muscle mass.

Introduction

The strength of bone is dependent on ~~bone~~ quantity and quality. Bone quantity is ~~simply evaluated~~ by bone volume and bone mineral density (BMD), ~~whereas various~~ parameters including architecture and geometry~~;~~ bone turnover~~;~~ cortical porosity~~;~~ damage~~;~~ mineralization~~;~~ and the properties of minerals, collagen, and non-collagenous proteins, have been proposed as ~~bone quality indices~~ [1]. Non-collagenous proteins play significant roles in the structural organization of bone and influence its mechanical properties [2]. Ocn~~, which~~ is synthesized by osteoblasts, is the most abundant non-collagenous protein in bone. The three glutamic acid residues are carboxylated~~, and~~ ~~c~~arboxylated Ocn has high affinity for Ca^{2+} and adopts an α-helical conformation by

binding to Ca^{2+}, whereas uncarboxylated Ocn has no affinity to Ca^{2+}. Carboxylated Ocn has been implicated in mineralization, inhibition of hydroxyapatite growth, and chemotactic activity of osteoclast precursors [3].

One Ocn gene (*BGLAP*) has been identified in humans, while mice have a gene cluster of Ocn that consists of *Bglap*, *Bglap2*, and *Bglap3* within a 23-kb span of genomic DNA [4-6]. *Bglap* and *Bglap2* are expressed in bone, while *Bglap3* is expressed in non-osteoid tissues, including the kidneys, lungs, and male gonadal tissues [6,7]. Ocn-deficient (Ocn$^{-/-}$) mice, in which *Bglap* and *Bglap2* are deleted, show increases in trabecular and cortical bone masses and bone strength by the enhanced bone formation [8]. On the other hand, a recent study reported no difference in cortical thickness between wild-type and the same Ocn$^{-/-}$ mouse line [9]. The Ocn$^{-/-}$ rat was generated, and the trabecular, but not cortical bone mass was greater than that in wild-type mice and bone was stronger [10]. A Fourier transform infrared microscopic (FTIR) analysis showed impaired mineral maturation in Ocn$^{-/-}$ femoral cortical bone [11], whereas a Raman microspectroscopic analysis revealed higher crystallinity in Ocn$^{-/-}$ femoral cortical bone than that in wild-type mice [12]. Furthermore, a small angle x-ray scattering (SAXS) analysis showed that the shape of mineral particles was shorter, thinner, and more disorganized in Ocn$^{-/-}$ mice than in wild-type mice [13]. Therefore, the functions of Ocn in bone formation, maintenance, and quality remain controversial and have yet to be clarified.

The analyses of Ocn$^{-/-}$ mice have also showed that uncarboxylated Ocn functions as a hormone that regulates glucose metabolism by enhancing β-cell proliferation, insulin secretion, and insulin sensitivity [14]. Although many clinical studies have been performed based on these findings, the relationship between uncarboxylated Ocn and

glucose metabolism remains ~~unclear~~ [15-24]. ~~Further, it has been~~ shown that male Ocn$^{-/-}$ mice have small testes and impaired fertility due to severely reduced testosterone synthesis, and that uncarboxylated Ocn regulates testosterone synthesis by binding to its receptor Gprc6a on Leydig cells in the testes [25]. ~~Although there are~~ three different global *Gprc6a*$^{-/-}$ mouse models, ~~the phenotypes of~~ glucose metabolism, fertility, and BMD are ~~controversial~~ [26-30]. ~~Further,~~ it ~~is also~~ controversial whether Gprc6a is involved in insulin secretion and is a receptor for Ocn in Leydig cells in testis and β cells in pancreas [25,29,31-35]. ~~Moreover, it has been~~ shown that Ocn$^{-/-}$ mice have ~~a~~ reduced muscle mass and uncarboxylated Ocn promotes the uptake and catabolism of glucose and fatty acids as well as protein synthesis in myofibers [36].

~~Since~~ only one line of Ocn$^{-/-}$ mice was analyzed in previous studies, we generated Ocn$^{-/-}$ mice in which *Bglap* and *Bglap2* ~~were~~ deleted to clarify the ~~controversial findings on~~ bone phenotypes and ~~reveal~~ the ~~essential~~ functions of Ocn.

Results

Trabecular and cortical bone masses are similar in wild-type and Ocn$^{-/-}$ mice

Ocn$^{-/-}$ mice were generated by replacing genomic DNA encompassing *Bglap* and *Bglap2* with the neo gene (Fig 1A). This replacement was confirmed by Southern blot and PCR analyses (Fig 1B, C). Real-time RT-PCR analysis showed that the expression of *Bglap* and *Bglap2* was absent in the osteoblast fraction of bone from Ocn$^{-/-}$ mice (Fig 1D). Serum osteocalcin was also absent in Ocn$^{-/-}$ mice (Fig 1E). μ-CT analyses were performed on male mice at 14 weeks, 6 months, and 9 months of age (Fig 1F-I) and female mice at 6 and 9 months of age (S1 Fig). Bone volume, trabecular thickness, and

trabecular number in the trabecular bone of femurs were similar between wild-type and Ocn$^{-/-}$ mice in both sexes (Fig 1F, H; S1 Fig A, C). In the cortical bone of femurs, cortical thickness was also similar between wild-type and Ocn$^{-/-}$ mice in both sexes (Fig 1G, I; S1 Fig B, D), while periosteal and endosteal perimeters were larger in male Ocn$^{-/-}$ mice than in male wild-type mice at 6 months of age, but not at 14 weeks or 9 months of age (Fig 1I). These differences were not observed in female mice (S1 Fig D).

Bone formation and resorption are similar in wild-type and Ocn$^{-/-}$ mice

Bone histomorphometric analysis was performed on male mice at 6 months of age. The parameters for osteoblasts, osteoclasts, osteocytes, and bone formation in the trabecular bone of femurs were similar between wild-type and Ocn$^{-/-}$ mice (Fig 2A). In cortical bone, the mineral apposition rate, mineralizing surface, and bone formation rate in the periosteum were similar between wild-type and Ocn$^{-/-}$ mice (Fig 2B), while the mineral apposition rate and bone formation rate in the endosteum were similar, whereas the mineralizing surface was smaller in Ocn$^{-/-}$ mice than in wild-type mice (Fig 2C).

Serum markers for bone formation (N-terminal propeptide of type I procollagen: P1NP) and bone resorption (tartrate-resistant acid phosphatase 5b: TRAP5b and C-terminal telopeptide crosslink of type I collagen: CTX1) were similar between wild-type and Ocn$^{-/-}$ mice at 11 weeks, 6 months, and 9 months of age (Fig 3A). The expression of osteoblast and osteoclast marker genes, including *Col1a1*, *Spp1*, *Alpl*, *Runx2*, *Sp7*, and *Ctsk*, was similar in wild-type and Ocn$^{-/-}$ mice at 6 months of age (Fig 3B). The expression of *Bglap* and *Bglap2* was not detected in Ocn$^{-/-}$ mice (Fig 3B).

To evaluate the function of Ocn in an estrogen-depleted state, ovariectomy was performed at 5 weeks of age and mice were analyzed at 11 weeks of age because bone

loss after ovariectomy is not apparent in adult B57BL/6 mice due to a low cancellous bone mass [37]. Bone volume in trabecular bone was significantly reduced in wild-type mice and marginally reduced in Ocn$^{-/-}$ mice, and the trabecular number was significantly lower in wild-type and Ocn$^{-/-}$ mice after ovariectomy than in the respective sham-operated mice (S2 Fig A, C). Ovariectomy did not affect cortical bone in wild-type or Ocn$^{-/-}$ mice (S2 Fig B, D), because B57BL/6 mouse line is relatively inseneitive to ovariectomy [38]. This is in contrast to previous findings, which showed more severe bone loss in Ocn$^{-/-}$ mice than in wild-type mice after ovariectomy [8]; therefore, ~~an~~ estrogen deficiency exerted similar effects in wild-type and Ocn$^{-/-}$ bone.

Ocn is localized in intrafibrillar and interfibrillar collagen regions and mineralization proceeds normally in Ocn$^{-/-}$ mice

Trabecular and cortical bone were similar between wild-type and Ocn$^{-/-}$ femurs in H-E-stained sections (S3 Fig). Furthermore, similar numbers of TRAP-positive cells were observed in wild-type and Ocn$^{-/-}$ femurs (S4 Fig). Transmission electron microscopy (TEM) analysis showed that collagen fibers generally ran parallel to the longitudinal direction of bone in wild-type and Ocn$^{-/-}$ mice (Fig 4A-F). The binding of the Ocn antibody was detected in the intrafibrillar and interfibrillar collagen regions of wild-type bone, but not Ocn$^{-/-}$ bone (Fig 4A-F). TEM analysis using non-decalcified sections revealed the presence of mineralized nodules, which are globular assemblies of numerous needle-shaped mineral crystals, in the osteoids of wild-type and Ocn$^{-/-}$ mice, indicating that mineralization proceeded similarly in wild-type and Ocn$^{-/-}$ mice (Fig 4G-J).

A Raman microspectroscopic analysis showed no significant differences in mineralization, crystallinity, carbonate to phosphate ratios, collagen maturity, or the remodeling index between wild-type and Ocn$^{-/-}$ mice

The chemical aspects of bone collagen and BAp were analyzed in the posterior part of the femurs at mid-diaphysis by Raman microspectroscopy (Fig 5A, B). The mineral to matrix ratio, which indicates the amount of mineralization; crystallinity, which indicates the degree of mineral crystallinity; carbonate to phosphate ratio, which varies with bone architecture, age, and mineral crystallinity; collagen maturity, which assesses non-reducible to reducible collagen crosslink ratios; and the remodeling index, which indicates bone remodeling [39], were similar between wild-type and Ocn$^{-/-}$ mice (Table 1, Fig 5C).

The alignment of collagen fibers was parallel to the longitudinal axis of bone and the size of BAp crystallites was normal in Ocn$^{-/-}$ mice, whereas the crystallographic *c*-axis orientation of BAp was severely disrupted, leading to a reduced Young's modulus

BMD was similar between female wild-type and Ocn$^{-/-}$ mice throughout the length of the femur, except at position 9 at 9 months of age (Fig 6A, C). To characterize the bone microstructure, the orientation of collagen was examined using birefringence measurements. The orientation of collagen was almost parallel to the longitudinal axis of bone in the diaphysis and gradually became disturbed from the metaphysis to epiphysis at similar levels in wild-type and Ocn$^{-/-}$ femurs (Fig 6B, D). The *c*-axis orientation degree of BAp that grows parallel to collagen in normal mineralization was analyzed using a microbeam X-ray diffraction (μXRD) system, which provides information on the atomic

arrangement of crystalline apatite [40]. The intensity ratio of (002)/(310), an index representing the preferential orientation degree of the BAp *c*-axis, parallel to the bone longitudinal axis, was markedly lower in Ocn$^{-/-}$ femurs than in wild-type femurs (Fig 6E). A regression analysis showed that the BAp *c*-axis orientation degree largely depended on the orientation degree of collagen in wild-type femurs, while this dependency (slope) was weaker in Ocn$^{-/-}$ femurs, demonstrating that the epitaxial relationship between collagen and BAp was significantly disrupted in Ocn$^{-/-}$ mice (Fig 6F). However, the size of BAp crystallites in the *c*-axis evaluated along the longitudinal and tangential axes of bone at mid-diaphysis was similar between wild-type and Ocn$^{-/-}$ femurs (Fig 7A). Similar results were obtained in male mice at 14 weeks of age for BMD and the orientations of collagen and BAp as well as the correlation between the orientations of collagen and the BAp *c*-axis (S5 Fig A-E).

To evaluate bone mechanical functions in the major loading direction, nanoindentation testing was performed along the bone longitudinal axis at the mid-diaphysis (position 5) in 9-month-old mouse femurs. Young's modulus was significantly lower in Ocn$^{-/-}$ femurs than in wild-type femurs (Fig 7B). Single and multiple regression analyses were performed using Young's modulus as a dependent variable and BMD, the collagen orientation degree, and apatite *c*-axis orientation degree as explanatory variables, all of which were taken at position 5. Young's modulus positively correlated with the BAp *c*-axis orientation degree, but not with BMD or the orientation of collagen in the single regression analysis (Fig 7C). A multiple regression analysis showed that Young's modulus was strongly and solely influenced by the orientation of BAp, but not by that of collagen or BMD (Table 2). The reduction in Young's modulus in Ocn$^{-/-}$ mice and the importance of the orientation of BAp to Young's modulus was also revealed in male mice

at 14 weeks of age (S5 Fig F and G, S1 Table). However, three-point bending test did not show any difference between wild-type and Ocn$^{-/-}$ mice in maximum load, displacement, stiffness, and energy to failure at 6 months of age, indicating that the three-point bending test is less sensitive than the nanoindentation testing for the evaluation of bone mechanical functions in the major loading direction (S6 Fig).

Glucose metabolism is normal in Ocn$^{-/-}$ mice

Body weights were similar between wild-type and Ocn$^{-/-}$ mice in both sexes from 11 weeks to 18 months of age, except for female Ocn$^{-/-}$ mice at 9 months of age (Fig 8A). Blood glucose and HbA1c levels were also similar between wild-type and Ocn$^{-/-}$ mice in both sexes at all ages (Fig 8B, C). Furthermore, the amounts of subcutaneous and visceral adipose tissues in Ocn$^{-/-}$ mice were similar to those in wild-type mice in 14 week-old male and 9-month-old female mice (Fig 8D-H). Glucose tolerance tests (GTTs) were performed by injecting glucose intraperitoneally into mice fed a normal or high-fat diet in both sexes from 14 weeks to 18 months of age. In all ages, both sexes, and both diets, serum glucose and insulin levels were similar between wild-type and Ocn$^{-/-}$ mice (Fig 9A-E, S7 Fig). Further, insulin tolerance tests (ITTs) were performed in male mice fed a normal or high-fat diet at 4 and 8 months of age. Serum glucose levels were also similar between wild-type and Ocn$^{-/-}$ mice (Fig 9F, G). These results indicate that Ocn is not physiologically involved in glucose metabolism.

Serum levels of carboxylated Ocn but not uncarboxylated Ocn are associated with an increase of bone formation and improvement of glucose metabolism by exercise

Since Ocn is one of the markers for bone formation and exercise affects glucose

metabolism and bone formation, we examined the relationships among exercise, glucose metabolism, and bone formation with a treadmill. In wild-type mice of the C57BL/6 strain with a normal diet, body weight, but not HbA1c, was lower in the exercise group than in the control group (Fig 10A, B). Serum levels of carboxylated Ocn, uncarboxylated Ocn, and P1NP were higher in the exercise group than in the control group. CTX-1 was not affected by exercise (Fig 10C). μ-CT analysis of trabecular bone showed that bone volume, trabecular number, and BMD increased after exercise (Fig 10D). In a mouse model (KK/TaJcl) of diabetes mellitus with a high-fat diet, exercise decreased body weight and HbA1c, increased carboxylated Ocn and P1NP, and had no effects on uncarboxylated Ocn or CTX-1 (Fig 10E-G). μ-CT analysis of trabecular bone showed that the trabecular number and BMD increased after exercise (Fig 10H). Cortical bone was unaffected by exercise in wild-type and KK/TaJcl mice (S8 Fig). These results indicate that exercise improves glucose metabolism and increases bone formation; similar changes were observed in the serum levels of carboxylated Ocn and P1NP after exercise, and uncarboxylated Ocn did not play a role in the improvements in glucose metabolism caused by exercise.

Testosterone synthesis, spermatogenesis, and muscle mass are normal in Ocn$^{-/-}$ mice

Male Ocn$^{-/-}$ mice were fertile and had testes of a similar size and weight to those of wild-type mice (Fig 11A, B). Furthermore, serum testosterone levels were similar between wild-type and Ocn$^{-/-}$ mice at 11 weeks, 6 months, and 18 months of age (Fig 11C). Histological analysis showed that spermatogenesis proceeded normally in the testes of Ocn$^{-/-}$ mice and the number of spermatozoa was similar between wild-type and Ocn$^{-/-}$ mice (Fig 11D, E, S9 Fig A-J). Moreover, the frequencies of the spermatozoa with

acrosomal defects were similar between them (Fig 11F, G). We examined germ cell apoptosis, which is inhibited by testosterone [41,42], in the seminiferous tubules by TUNEL staining. The number of TUNEL-positive germ cells was similar between wild-type and Ocn$^{-/-}$ mice (Fig 11H, I). We also examined the expression of the genes, including *Star*, *Cyp11a1*, *Cyp17a1*, and *Hsd3b2*, which encode the enzymes that are necessary for testosterone biosynthesis. The expression levels of these genes in Ocn$^{-/-}$ testes were similar to those in wild-type testes (Fig 11J).

The weights of muscles, including the quadriceps, gastrocnemius, soleus, and extensor digitorum longus, and the average area of muscle fibers in histological sections were similar between wild-type and Ocn$^{-/-}$ mice (Fig 12, S9 Fig K-N). These results indicate that Ocn is not physiologically involved in testosterone synthesis, spermatogenesis, and the maintenance of muscle mass.

Discussion

In the present study, an Ocn deficiency did not affect bone mass, BMD, bone formation, or bone resorption. Furthermore, bone loss after ovariectomy was similar in wild-type and Ocn$^{-/-}$ mice. These results indicate that Ocn is not involved in the regulation of bone quantity; however, its effects on bone ~~quantity~~ remain controversial in the literature [8-10]. The *c*-axis orientation of BAp along the femur longitudinal axis was severely disrupted in Ocn$^{-/-}$ mice, despite the orientation of collagen along the same axis r~~emaining~~ unchanged, ~~which led~~ to the deterioration of mechanical properties. These results demonstrate that Ocn plays a role in the epitaxial growth of BAp on the collagen template and is essential for ensuring that the directionality of the BAp *c*-axis is parallel to collagen fibrils, which run longitudinally to bone. Thus, Ocn plays an important role

in maintaining bone quality and strength by adjusting the crystallographic orientation of the BAp *c*-axis parallel to collagen fibrils However, random blood glucose and HbA1c levels, the amounts of subcutaneous and visceral adipose tissues, glucose and insulin levels in GTTs, and glucose levels in ITTs in Ocn$^{-/-}$ mice were similar to those in wild-type mice in both sexes fed a normal or high-fat diet and at all ages examined. Further, testis weights, serum testosterone levels, the numbers of spermatozoa and TUNEL-positive germ cells, and the expression of the genes encoding the enzymes necessary for testosterone biosynthesis were normal in Ocn$^{-/-}$mice. Moreover, muscle mass was normal in Ocn$^{-/-}$mice. These results indicate that osteocalcin is not physiologically involved in glucose metabolism, testosterone synthesis, or the maintenance of muscle mass.

In normal mineralization, the *c*-axis of apatite, which is the predominant direction of crystal growth, aligns almost parallel to the direction of collagen fibrils, which preferentially run along the longitudinal axis of long bones, due to the epitaxial crystallization of apatite on the collagen template [43]. Immunolocalization clarified that Ocn exists in intrafibrillar and interfibrillar collagen regions (Fig 4A, B, D, E), whereas another non-collagenous protein, bone sialoprotein, is only present in interfibrillar collagen regions in mineralized tissue [44]. These findings suggest the involvement of Ocn in the bone mineralization process *in vivo*. Poundarik et al. recently reported that Ocn$^{-/-}$ mice have smaller and less organized BAp particles along the longitudinal axis of femurs by a SAXS analysis, which provides information on the shape of bone apatite particles, but not the crystallographic orientation (atomic arrangement) [13]. Further the orientation of collagen has not yet been examined [13]. Hence, the directional relationship between collagen and BAp remains unclear [13]. The present results revealed that collagen fibers preferentially oriented along the longitudinal axis in Ocn$^{-/-}$ femurs with

an identical orientation degree to wild-type femurs (Fig 6B, D). Furthermore, the results of the μXRD analysis showed the severely disrupted orientation of the BAp *c*-axis in Ocn$^{-/-}$ femurs (Fig 6E). Therefore, ~~the present~~ results reveal~~ed~~ for the first time the disruption of the crystallographic orientation of BAp in Ocn$^{-/-}$ bone, ~~which had~~ the normal orientation of collagen fibers.

The orientations of collagen and the BAp *c*-axis have been investigated in several ~~bones~~, including *oim*/*oim* osteogenesis imperfecta, c-src$^{-/-}$ osteopetro~~tic~~, melanoma metastas~~ized~~, unloade~~d,~~ and regenerating long bones [40,45-48]. Different from the Ocn$^{-/-}$ bone, the orientation of collagen was disrupted and, as a result, the orientation of the BAp *c*-axis along the bone longitudinal axis was severely disrupted. In these bones, an epitaxial relationship still exists between collagen and BAp. Thus, ~~this~~ is the first study to ~~indicate~~ that the orientation of the BAp *c*-axis was disrupted even though collagen fibrils ran in the correct direction (longitudinal axis of the long bone)~~, and the~~ results ~~obtained~~ demonstrate that Ocn is essential for the alignment of the BAp *c*-axis to the direction of collagen fibrils.

The present study also show~~ed~~ that the *c*-axis of apatite crystallites is not always aligned synchronously along the orientation of collagen and, therefore, collagen is not the only factor influencing the orientation of apatite, which is consistent with the findings of Glimcher [49]. According to biomechanics, the orientations of collagen and apatite contribute differently to bone mechanical properties; highly oriented collagen mainly contributes to the prevention of crack propagation along the vertical direction against the collagen running axis [50], while highly oriented apatite *c*-axes predominantly contribute to stiffening the bone matrix [48]. Individual analyses on the orientations of collagen and apatite are needed to understand the functions of bone, clarify the pathology of bone

diseases, and select optimal anti-bone disease agents. Furthermore, Young's modulus strongly correlated with the orientation of the BAp *c*-axis, but not that of collagen or BMD. Thus, these results indicate that the reduction observed in Young's modulus was due to the disrupted alignment of the BAp *c*-axis to the longitudinal axis of bone, demonstrating that the orientation of the BAp *c*-axis to the longitudinal axis of bone as a result of the influence of osteocalcin is important for maintaining bone strength.

Figure 7D shows a schematic view of the directional relationship between collagen fibrils and the BAp *c*-axis in wild-type and $Ocn^{-/-}$ mice. Collagen fibrils and the BAp *c*-axis co-align parallel to the longitudinal axis in normal bone, whereas the orientation of the BAp *c*-axis is severely disrupted in spite of normally oriented collagen fibrils parallel to the bone longitudinal axis in $Ocn^{-/-}$ bone, leading to a reduction in Young's modulus in the longitudinal direction. To clarify how Ocn aligns the BAp *c*-axis to the direction of collagen fibrils, interactions between Ocn and collagen and between Ocn and BAp need to be investigated. The binding potential of Ocn to Ca^{2+} in the (001) crystallographic plane of apatite lattices [51] is considered to be a key in BAp growth toward a specific direction along the orientation of collagen. Regarding the directional (epitaxial) relationship, a simulation study of ionic clustering behavior—the very early stage of BAp nucleation—under an interaction with affector molecules will be a possible solution [52].

Many clinical studies showed a positive relationship between total Ocn and glucose metabolism [53-58]; however, that between uncarboxyleted Ocn and glucose metabolism remains controversial [15-24]. Exercise reduced body weight and HbA1c and increased serum Ocn, P1NP, and BMD in KK/TaJcl mice (Fig 10E-H), indicating that exercise improves glucose metabolism and serum Ocn increases due to enhanced bone formation. In previous studies showing a relationship between serum total Ocn and glucose

metabolism, the serum levels of bone-specific alkaline phosphatase, which is a marker for bone formation, were elevated [59,60], suggesting that the increase in serum total Ocn was due to enhanced bone formation. Thus, the relationship between Ocn and glucose metabolism ~~reported by many clinical studies~~ may be ~~explained~~ by ~~that between~~ bone formation ~~and glucose metabolism~~.

The phenotypes of increased bone mass, impaired glucose metabolism, and reduced testosterone synthesis and muscle mass in the ~~previous~~ Ocn$^{-/-}$ mice were not observed in our Ocn$^{-/-}$ mice, ~~although~~ both *Bglap* and *Bglap2* were deleted and *Bglap3*, ~~which is expressed in non-osteoid tissues with unknown function,~~ was intact in both Ocn$^{-/-}$ mouse lines. The only difference between the two mouse lines is the genetic background. Our Ocn$^{-/-}$ mice were backcrossed to C57BL/6N more than 8 times before analysis, while the previous Ocn$^{-/-}$ mice were analyzed in the mixed background of C57BL/6J and 129/SV. Since the difference of the genetic background is unlikely to explain all controversial phenotypes, our Ocn$^{-/-}$ mouse line will be shared with other researchers to resolve the controversies.

In conclusion, Ocn is not necessary for the regulation of bone formation or resorption, but is essential for the alignment of the BAp *c*-axis parallel to collagen fibrils and is required ~~to maintain~~ bone strength. ~~However,~~ Ocn does not work as a hormone that regulates glucose metabolism, testosterone synthesis, and muscle mass.

Methods

Generation of Ocn$^{-/-}$ mice

The targeting vector for the mouse *Bglap* and *Bglap2* genes was generated by the BAC modification technique as previously described [61]. The BAC modification kit was

purchased from Gene Bridges (Heidelberg, Germany), and the BAC clone (RF24-298F19) was purchased from the BACPAC Resource Center (Oakland, CA). The cassette containing PGK-gb2-neo was PCR amplified and introduced into bacteria containing the BAC clone to replace genomic DNA encompassing *Bglap* and *Bglap2*. The cassette containing PGK-gb2-neo flanked by 5' DNA of *Bglap* and 3' DNA of *Bglap2* was cloned into the plasmid containing the PGK-tk cassette. The final targeting construct was electroporated into the E14 line of ES cells, and targeted ES cells were injected into C57BL/6 blastocysts. The mutant allele was confirmed by a Southern blot analysis as described previously [62]. $Ocn^{+/-}$ mice were backcrossed with C57BL/6N 8-10 times. Prior to the study, all experiments were reviewed and approved by the Animal Care and Use Committee of Nagasaki University Graduate School of Biomedical Sciences (No. 1903131520-2). Animals were housed 3 per cage in a pathogen-free environment on a 12-h light cycle at 22±2°C with standard chow (CLEA Japan, Tokyo) and had free access to tap water.

Real-time RT-PCR analysis

Total RNA was extracted using ISOGEN (Wako, Osaka, Japan). Osteoblast-enriched cells were collected as previously described [63]. Real-time RT-PCR was performed on RNA isolated from the femurs or testes of individual mouse using the primers shown in Supplementary Table 2 as previously described [64]. We normalized the values obtained to those of *β-actin*.

μ-CT, bone histomorphometry, and TEM

Dissected femurs were analyzed by μ-CT systems (R_mCT; Rigaku Corporation, Tokyo,

Japan) as previously described [63]. The volume of adipose tissue was analyzed by the μ-CT system using 3D fat analysis software (Rigaku Corporation). Bone histomorphometric analyses were performed as previously described [65]. In the assessment of dynamic histomorphometric indices, mice were injected with calcein 6 and 2 d before sacrifice at a dose of 0.16 mg/10 g body weight. In the immunoelectron microscopic analysis, mice were perfusion-fixed with 0.01% glutaraldehyde-4% paraformaldehyde in 0.05 M cacodylate buffer (pH7.4). The dissected long bones were further immersed in the same fixatives at 4°C for 24 h, decalcified in 5% EDTA (pH7.4) at 4°C for 2-4 weeks, dehydrated with ascending concentrations of ethanol, and embedded in LR-white resin (London Resin Company Ltd., Berkshire, UK). Ultrathin sections (90 nm) were immunolabeled with a rabbit anti-Ocn antibody (Takara Bio Inc., Shiga, Japan), followed by a 20-nm colloidal gold-conjugated secondary antibody to Rabbit IgG (BBI Solutions, Cardiff, UK), and post-stained with 2% uranyl acetate. In the TEM analysis of non-decalcified specimens, mice were perfusion-fixed with 2.5% glutaraldehyde-2% paraformaldehyde in 0.1 M cacodylate buffer (pH7.4). The dissected long bones were further immersed in the same fixatives at 4°C for 24 h, post-fixed with 1% OsO_4 in 0.1 M cacodylate buffer (pH7.4) at 4°C for 4 h, dehydrated with ascending concentrations of acetone, and embedded in epoxy resin (Taab, Berkshire, UK).

Assays for Ocn, markers for bone formation and resorption, and testosterone

The serum level of total Ocn was examined using the Ocn EIA kit (BTI Biomedical Technologies, Inc., Stoughton. MA), that of carboxyleted Ocn by the Mouse Gla-Osteocalcin High Sensitive EIA Kit (Takara Bio Inc., Shiga Japan), that of uncarboxyleted Ocn by the Mouse Glu-Osteocalcin High Sensitive EIA Kit (Takara Bio

Inc.), that of P1NP by Rat/Mouse P1NP ELISA (Immunodiagnostic Systems, Boldon, UK), that of TRAP5b by the MouseTRAP™ Assay (Immunodiagnostic Systems), and that of CTX1 by RatLaps™ (CTX-I) EIA (Immunodiagnostic Systems). Serum testosterone levels were assessed using Testosterone Rat/Mouse ELISA (Demeditec Diagnostics GmbH, Kiel, Germany).

Confocal Raman spectroscopy

The area (80 × 60 μm) adjacent to the endosteal surface of the posterior side in slices of the mid-diaphysis of femurs were analyzed by a confocal Raman microscope (Nanofinder HE, Tokyo Instruments Inc., Japan). A 532-nm laser was focused on 200 fields in the area with 2-μm spot sizes. Each field was exposed for 0.1 sec twice, and Raman spectra was obtained by totalizing 400 values in each sample.

Analysis of BMD and collagen orientation

Volumetric BMD was measured at 9 points (regular intervals of 1/10 of the bone length along the longitudinal axis) on femurs at 36 weeks of age using an XCT Research SA+ system (STRATEC Medizintechnik Gmbh, Birkenfeld, Germany) with a resolution of 70 × 70 × 260 μm as described previously [40]. The femurs at 9 months of age were decalcified and embedded in paraffin, and sagittal sections (10 μm) were deparaffinized and observed using a two-dimensional birefringence analyzer (WPA-micro, Photonic Lattice, Miyagi, Japan). Collagen orientation was assessed at 9 points in the anterior and posterior sides of cortical bone in femurs as previously described [40]. To evaluate the collagen orientation degree along the longitudinal direction of the femur, the orientation order parameter f_θ was calculated based on the angle distribution of collagen against the

bone longitudinal direction as previously described [66]. f_θ takes a value ranging from -1 (collagen perfectly aligned perpendicular to the bone longitudinal direction) to 1 (collagen perfectly aligned parallel to the bone longitudinal direction).

Analysis of the BAp *c*-axis orientation

The crystallographic orientation of apatite crystallites was analyzed by the μXRD system (R-Axis BQ, Rigaku, Tokyo, Japan), which is equipped with a transmission-type optical system and an imaging plate (storage phosphors) (Fuji Film, Tokyo, Japan), at 9 points (regular intervals of 1/10 of the bone length along the longitudinal axis) of cortical bone in femurs as previously described [40] with a minor modification in that the incident beam was focused on a beam spot of 300 μm in diameter and diffraction data were collected for 180 sec. The preferential orientation degree of the BAp *c*-axis was assessed as the relative intensity ratio of the (002) diffraction peak to the (310) peak in the X-ray profile.

Analysis of the size of BAp crystallites

The average size of BAp crystallites in the *c*-axis was calculated based on the Scherrer formula using the half width of the diffraction peak profile of (002) along the longitudinal and tangential axes of bone analyzed by the microbeam X-ray diffractometer system (D8 DISCOVER with GADDS, Bruker AXS, WI, USA). Cu-Kα radiation with a wavelength of 0.15418 nm was generated at a tube voltage of 45 kV and a tube current of 110 mA. An incident beam was focused on a beam spot of 100 μm and diffraction data were counted with a two-dimensional position sensitive proportional counter (PSPC) for 600 sec. The half width of the observed diffraction profile was corrected by that of hydroxyapatite (NIST #2910: calcium hydroxyapatite) to acquire the half width of the

pure profile.

Nanoindentation testing

Young's modulus was measured along the longitudinal direction of the femur using midshaft specimens (position 5) by a nanoindentation system (ENT-1100a; Elionix, Tokyo, Japan) with a Berkovich diamond indenter as previously described [40]. Young's modulus was assessed according to the method of Oliver and Pharr [67].

Metabolic assessments

Regarding GTTs, glucose (1 or 2 g/kg body weight) was injected intraperitoneally after fasting for 15 hours. Regarding ITTs, insulin (1 U/kg body weight) was injected intraperitoneally after fasting for 6 hours. Blood glucose was measured using Glutest Neo Super (Sanwa Kagaku Kenkyusho Co., Ltd., Nagoya Japan). Serum insulin was measured using the Mouse Insulin ELISA Kit (Morinaga Institute of Biological Science, Inc., Yokohama, Japan). HbA1c was measured using the Mouse HbA1c ELISA Kit (Aviva Systems Biology, San Diego, CA). Mice were fed a normal diet (CE-2, CLEA Japan Inc., Tokyo, Japan) or high-fat diet (HFD-32, CLEA Japan Inc.).

Exercise on a treadmill

Wild-type (C57BL/6) mice were fed a normal diet, and the mouse model for type 2 diabetes mellitus, KK/TaJcl (CLEA Japan Inc.), was fed a normal diet until one week before the exercise study and then fed a high-fat diet. Mice were divided into two groups with or without exercise. Mice were forced to exercise on a treadmill (Natsume Seisakusho Co., Ltd., Tokyo, Japan) with an electric shock at 20V/50mA to trigger

running. After running training for 3 days, mice ran for 30 min per day at a speed of 12 m/min on the treadmill with a tilt of 5 degrees. Running continued 5 days per week for 7 weeks. Blood sampling was performed the day after the last exercise session.

Histological analysis

Mice were perfusion-fixed with 4% paraformaldehyde/0.01 M phosphate-buffered saline. Testes with epididymides and muscle were embedded in paraffin. Sections (thickness of 3 μm) were stained with hematoxylin and eosin (H-E) or stained for TUNEL using the ApopTag® Peroxidase In Situ Apoptosis Detection Kit (Sigma Aldrich, St. Louis, MO).

Analysis of sperm

Seminal fluid was collected from the cauda epididymides of male mice at 8 months of age and the weight was measured. The sperm were incubated with the medium (FERTIUP® Kyudo Co. Ltd. Saga, Japan) containing Hoechst 33348 (1.5μg/ml, Sigma Aldrich) for 1 hour. After incubation, the medium containing sperm was diluted with PBS and the number of sperm was counted. For analysis of sperm morphology, the sperm was observed by fluorescence microscope (All-in-one Fluorescence Microscope BX-X710, KEYENCE, Osaka, Japan). Further, the diluted medium containing sperm was smeared to slide glasses and the smears were stained with Wright-Giemsa and analyzed with a Zeiss Axioskop 2 pus microscope (Carl Zeiss, Tokyo, Japan) with a Olympus DP74 camera (Olympus Co., Tokyo, Japan).

Box plots and satistical analysis

Data were shown using the box plots to visually show the distribution of numerical data and skewness by displaying the data quartiles and averages. The outliers were defined as data points located outside 1.5 times the interquartile range (IQR, shown as a box) above the upper quartile or those located outside 1.5 times the IQR below the lower quartile. Statistical analyses of two groups were performed using the Student's *t*-test, and those of more than three groups were performed by one-way ANOVA and the Tukey-Kramer post hoc test. A P-value of less than 0.05 was considered to be significant. These statistical analyses and single and multiple regression analyses were performed using BellCurve for Excel (Social Survey Research Information Co., Ltd., Tokyo, Japan).

Supporting information

S1 Fig. μ-CT analysis of femurs in female wild-type and Ocn$^{-/-}$ mice at 6 and 9 months of age. (A and B). μ-CT images of femoral distal metaphyses (A) and mid-diaphyses (B). Scale bars = 500 μm. (C) Trabecular bone parameters, including the trabecular bone volume (BV/TV), trabecular thickness (Tb.Th), and trabecular number (Tb.N). (D) Cortical bone parameters including cortical thickness (Ct. Th), the periosteal perimeter (Ps.Pm), and endocortical perimeter (Ec.Pm). wt (n=6), Ocn$^{-/-}$ (n=8) at 6m; wt (n=5), Ocn$^{-/-}$ (n=6) at 9m. X symbols in box plots show outliers. (TIF)

S2 Fig. μ-CT analysis and serum markers after ovariectomy. Sham operation or ovariectomy (OVX) was performed at 5 weeks of age and mice were analyzed at 11 weeks of age. (A-D) μ-CT analyses of femurs in wild-type and Ocn$^{-/-}$ mice with sham operation or OVX. μ-CT images of femoral distal metaphyses (A) and mid-diaphyses (B) are shown. Scale bars = 500 μm. C, Trabecular bone parameters, including the trabecular bone volume (BV/TV), trabecular thickness (Tb.Th), and trabecular number (Tb.N). D, Cortical bone parameters including cortical thickness (Ct. Th), the periosteal perimeter (Ps.Pm), and endocortical perimeter (Ec.Pm). wt sham: n=9, wt OVX: n=8, Ocn$^{-/-}$ sham: n=6, Ocn$^{-/-}$ OVX: n=7. X symbols in box plots show outliers. (TIF)

S3 Fig. H-E-stained sections of wild-type and Ocn$^{-/-}$ femurs at 14 weeks of age.

(A, C, E) Wild-type mice. (B, D, F) Ocn$^{-/-}$ mice. The boxed regions in A are magnified in C and E, and those in B are magnified in D and F. Scale bars: 0.5 mm (A, B), 200 μm (C, D), 50 μm (E, F). (TIF)

S4 Fig. Histological analysis of osteoclasts.

Sections of femurs in wild-type (A, C) and Ocn$^{-/-}$ (B, D) mice at 14 weeks of age were stained with TRAP. The boxed regions in A and B are magnified in C and D, respectively. Scale bars: 200 μm (A, B), 100 μm (C, D). (TIF)

S5 Fig. BMD, orientations of collagen fibers and the BAp *c*-axis, and Young' modulus in the femoral cortical bone of male wild-type and Ocn$^{-/-}$ mice at 14 weeks of age.

(A) Schematic presentation of analyzed positions. (B) BMD at position 5. (C) Collagen orientation degree. (D) BAp *c*-axis orientation degree. (E) Single regression analysis of the orientations of collagen fibers and the BAp *c*-axis. (F) Young's modulus along the bone longitudinal axis at position 5. **: P<0.01. X symbols in box plots show outliers. (G) Single regression analysis of Young's modulus to BMD and each degree of the preferential alignment of collagen fibers and the BAp *c*-axis in the bone longitudinal direction at position 5. wt: blue dots, Ocn$^{-/-}$: red dots. n=6. (TIF)

S6 Fig. The three-point bending test.

Representative load-displacement curves for male wild-type (A) and Ocn$^{-/-}$ (B) mice at 6 months of age, in which maximum load, displacement, stiffness (the slope of the linear part of the load), and energy to failure (area under the load-displacement curve) were obtained. C, Maximum load, displacement, stiffness, and energy in wild-type (n=8) and Ocn$^{-/-}$ (n=6) mice. The experiments were performed as previously described (J Bone Miner Res 2016; 31: 1366-1380.). (TIF)

S7 Fig. GTTs.

Glucose (1 g/kg body weight) was injected intraperitoneally in GTTs. (A) Glucose levels in male mice at 14 weeks of age fed a normal diet. wt: n=5, $Ocn^{-/-}$: n=6. (B) Glucose levels in female mice at 9 months of age fed a normal diet. wt: n=4, $Ocn^{-/-}$: n=6. (C and D) Glucose (C) and insulin (D) levels in male mice at 18 months of age fed a normal diet. wt: n=7, $Ocn^{-/-}$: n=5. (E) Glucose levels in male mice at 14 weeks of age fed a high-fat diet for 5 weeks. wt: n=5, $Ocn^{-/-}$: n=4. (F) Glucose levels in female mice at 14 weeks of age fed a high-fat diet for 5 weeks. wt: n=7, $Ocn^{-/-}$: n=5. (G) Glucose levels in female mice at 9 months of age fed a high-fat diet for 11 weeks. wt: n=4, $Ocn^{-/-}$: n=5. (TIF)

S8 Fig. μ-CT analysis of cortical bone.

Male wild-type (A) and KK/TaJcl (B) mice with or without exercise on a treadmill for 7 weeks were analyzed by μ-CT at 4 months of age. Cortical thickness (Ct. Th), the periosteal perimeter (Ps.Pm), endocortical perimeter (Ec.Pm), and BMD are shown. Control: n=13, treadmill: n=13 in wild-type mice. Control: n=15, treadmill: n=14 in KK/TaJcl mice. X symbols in box plots show outliers. (TIF)

S9 Fig. Analyses of spermatogenesis and muscle.

(A-J) Histological sections of testis and epididymis at 4 months of age stained with H-E. Testis and epididymis at low magnification (A, B), seminiferous tubules (C-F), and cauda epididymis (G-H) are shown. Bars: 1mm (A, B); 100 μm (C, D, G, H); and 20 μm (E, F, I, J). (K-N) Muscle weights (K), H-E stained sections (L, M), and the average areas of myofibers (N) in gastrocnemius muscle in male wild-type (n=8) and $Ocn^{-/-}$ (n=7) mice at 9 months of age. Bars: 50 μm. X symbols in box plots show outliers. (TIF)

S1 Table. Multiple regression analysis using the BAp *c*-axis orientation, collagen orientation, and BMD as explanatory variables and Young's modulus as a response variable at 14 weeks of age. β: standard partial regression coefficient. (XLSX)

S2 Table. Primer sequences. (XLSX)

Acknowledgements

We thank H. Sano for technical assistant in confocal Raman spectroscopy and S. Hamachi for secretarial assistance.

References

1 Boskey, A. L. & Imbert, L. Bone quality changes associated with aging and disease: a review. *Ann. N.Y. Acad. Sci* **1410**, 93-106, doi:10.1111/nyas.13572 (2017).

2 Sroga, G. E. & Vashishth, D. Effects of bone matrix proteins on fracture and fragility in osteoporosis. *Current osteoporosis reports* **10**, 141-150, doi:10.1007/s11914-012-0103-6 (2012).

3 Hauschka, P. V., Lian, J. B., Cole, D. E. & Gundberg, C. M. Osteocalcin and matrix Gla protein: vitamin K-dependent proteins in bone. *Physiological reviews* **69**, 990-1047, doi:10.1152/physrev.1989.69.3.990 (1989).

4 Celeste, A. J. *et al.* Isolation of the human gene for bone gla protein utilizing mouse and rat cDNA clones. *EMBO j* **5**, 1885-1890 (1986).

5 Rahman, S. *et al.* Multiple copies of the bone-specific osteocalcin gene in mouse and rat. *Endocrinology* **133**, 3050-3053, doi:10.1210/endo.133.6.8243336 (1993).

6 Desbois, C., Hogue, D. A. & Karsenty, G. The mouse osteocalcin gene cluster contains three genes with two separate spatial and temporal patterns of expression. *J biol chem* **269**, 1183-1190 (1994).

7 Sato, M. & Tada, N. Preferential expression of osteocalcin-related protein mRNA in gonadal tissues of male mice. *Biochem Biophys Res Commun.* **215**, 412-421, doi:10.1006/bbrc.1995.2480 (1995).

8 Ducy, P. *et al.* Increased bone formation in osteocalcin-deficient mice. *Nature* **382**, 448-452, doi:10.1038/382448a0 (1996).

9 Bailey, S., Karsenty, G., Gundberg, C. & Vashishth, D. Osteocalcin and osteopontin influence bone morphology and mechanical properties. *Ann N Y Acad Sci* **1409**, 79-84, doi:10.1111/nyas.13470 (2017).

10 Lambert, L. J. *et al.* Increased trabecular bone and improved biomechanics

in an osteocalcin-null rat model created by CRISPR/Cas9 technology. *Dis Model Mech* **9**, 1169-1179, doi:10.1242/dmm.025247 (2016).

11 Boskey, A. L. *et al.* Fourier transform infrared microspectroscopic analysis of bones of osteocalcin-deficient mice provides insight into the function of osteocalcin. *Bone* **23**, 187-196 (1998).

12 Kavukcuoglu, N. B., Patterson-Buckendahl, P. & Mann, A. B. Effect of osteocalcin deficiency on the nanomechanics and chemistry of mouse bones. *J Mech Behav Biomed Mater* **2**, 348-354, doi:10.1016/j.jmbbm.2008.10.010 (2009).

13 Poundarik, A. A., Boskey, A., Gundberg, C. & Vashishth, D. Biomolecular regulation, composition and nanoarchitecture of bone mineral. *Scientific reports* **8**, 1191, doi:10.1038/s41598-018-19253-w (2018).

14 Lee, N. K. *et al.* Endocrine regulation of energy metabolism by the skeleton. *Cell* **130**, 456-469, doi:10.1016/j.cell.2007.05.047 (2007).

15 Hwang, Y. C., Jeong, I. K., Ahn, K. J. & Chung, H. Y. The uncarboxylated form of osteocalcin is associated with improved glucose tolerance and enhanced β‐cell function in middle‐aged male subjects. *Diabetes-Metab Res* **25**, 768-772 (2009).

16 Kanazawa, I. *et al.* Serum undercarboxylated osteocalcin was inversely associated with plasma glucose level and fat mass in type 2 diabetes mellitus. *Osteoporosis Int* **22**, 187-194 (2011).

17 Iki, M. *et al.* Serum undercarboxylated osteocalcin levels are inversely associated with glycemic status and insulin resistance in an elderly Japanese male population: Fujiwara-kyo Osteoporosis Risk in Men (FORMEN) Study. *Osteoporosis Int* **23**, 761-770 (2012).

18 Díaz-López, A. *et al.* Reduced serum concentrations of carboxylated and undercarboxylated osteocalcin are associated with risk of developing type 2 diabetes mellitus in a high cardiovascular risk population: a nested case-control study. *J Clin Endocrinol Metab* **98**, 4524-4531 (2013).

19 Okuno, S. *et al.* Significant inverse relationship between serum undercarboxylated osteocalcin and glycemic control in maintenance hemodialysis patients. *Osteoporosis international : a journal established as*

result of cooperation between the European Foundation for Osteoporosis and the National Osteoporosis Foundation of the USA **24**, 605-612, doi:10.1007/s00198-012-2003-0 (2013).

20 Takashi, Y. *et al.* Undercarboxylated osteocalcin can predict insulin secretion ability in type 2 diabetes. *J Diabetes Investig* **8**, 471-474, doi:10.1111/jdi.12601 (2017).

21 Shea, M. K. *et al.* Gamma-carboxylation of osteocalcin and insulin resistance in older men and women. *Am j clin nutr* **90**, 1230-1235, doi:10.3945/ajcn.2009.28151 (2009).

22 Mori, K. *et al.* Undercarboxylated osteocalcin does not correlate with insulin resistance as assessed by euglycemic hyperinsulinemic clamp technique in patients with type 2 diabetes mellitus. *Diabetology & metabolic syndrome* **4**, 53 (2012).

23 Giudici, K. V. P., Fisberg, R. M., Marchioni, D. M. L., Peters, B. S. E. & Martini, L. A. Crosstalk Between Bone and Fat Tissue: Associations Between Vitamin D, Osteocalcin, Adipokines, and Markers of Glucose Metabolism Among Adolescents. *J Am Coll Nutr* **36**, 273-280, doi:10.1080/07315724.2016.1274923 (2017).

24 Starup-Linde, J. *et al.* Glucose Tolerance Tests and Osteocalcin Responses in Healthy People. *Front Endocrinol* **9**, 356, doi:10.3389/fendo.2018.00356 (2018).

25 Oury, F. *et al.* Endocrine regulation of male fertility by the skeleton. *Cell* **144**, 796-809, doi:10.1016/j.cell.2011.02.004 (2011).

26 Pi, M. *et al.* GPRC6A null mice exhibit osteopenia, feminization and metabolic syndrome. *PloS one* **3**, e3858, doi:10.1371/journal.pone.0003858 (2008).

27 Wellendorph, P. *et al.* No evidence for a bone phenotype in GPRC6A knockout mice under normal physiological conditions. *J Mol Endocrinol* **42**, 215-223, doi:10.1677/jme-08-0149 (2009).

28 Jorgensen, C. V. *et al.* Metabolic and skeletal homeostasis are maintained in full locus GPRC6A knockout mice. *Scientific reports* **9**, 5995, doi:10.1038/s41598-019-41921-8 (2019).

29 Smajilovic, S. *et al.* The L-alpha-amino acid receptor GPRC6A is expressed in the islets of Langerhans but is not involved in L-arginine-induced insulin release. *Amino acids* **44**, 383-390, doi:10.1007/s00726-012-1341-8 (2013).

30 Pi, M. *et al.* Impaired osteoblast function in GPRC6A null mice. *J Bone Miner Res* **25**, 1092-1102, doi:10.1359/jbmr.091037 (2010).

31 Pi, M., Wu, Y. & Quarles, L. D. GPRC6A mediates responses to osteocalcin in beta-cells in vitro and pancreas in vivo. *J Bone Miner Res* **26**, 1680-1683, doi:10.1002/jbmr.390 (2011).

32 Pi, M., Wu, Y., Lenchik, N. I., Gerling, I. & Quarles, L. D. GPRC6A mediates the effects of L-arginine on insulin secretion in mouse pancreatic islets. *Endocrinology* **153**, 4608-4615, doi:10.1210/en.2012-1301 (2012).

33 Pi, M. *et al.* Evidence for Osteocalcin Binding and Activation of GPRC6A in beta-Cells. *Endocrinology* **157**, 1866-1880, doi:10.1210/en.2015-2010 (2016).

34 Rueda, P. *et al.* Murine GPRC6A Mediates Cellular Responses to L-Amino Acids, but Not Osteocalcin Variants. *PloS one* **11**, e0146846, doi:10.1371/journal.pone.0146846 (2016).

35 Wei, J., Hanna, T., Suda, N., Karsenty, G. & Ducy, P. Osteocalcin promotes beta-cell proliferation during development and adulthood through Gprc6a. *Diabetes* **63**, 1021-1031, doi:10.2337/db13-0887 (2014).

36 Karsenty, G. & Olson, E. N. Bone and Muscle Endocrine Functions: Unexpected Paradigms of Inter-organ Communication. *Cell* **164**, 1248-1256, doi:10.1016/j.cell.2016.02.043 (2016).

37 Iwaniec, U. T., Yuan, D., Power, R. A. & Wronski, T. J. Strain-dependent variations in the response of cancellous bone to ovariectomy in mice. *J Bone Miner Res* **21**, 1068-1074, doi:10.1359/jbmr.060402 (2006).

38 Klinck, J. & Boyd, S. K. The magnitude and rate of bone loss in ovariectomized mice differs among inbred strains as determined by longitudinal in vivo micro-computed tomography. *Calcified Tissue Int* **83**, 70-79, doi:10.1007/s00223-008-9150-5 (2008).

39 Morris, M. D. & Mandair, G. S. Raman assessment of bone quality. *Clin Orthop Relat R* **469**, 2160-2169, doi:10.1007/s11999-010-1692-y (2011).

40 Ishimoto, T., Sato, B., Lee, J. W. & Nakano, T. Co-deteriorations of anisotropic extracellular matrix arrangement and intrinsic mechanical property in c-src deficient osteopetrotic mouse femur. *Bone* **103**, 216-223, doi:10.1016/j.bone.2017.06.023 (2017).

41 Brinkworth, M. H., Weinbauer, G. F., Schlatt, S. & Nieschlag, E. Identification of male germ cells undergoing apoptosis in adult rats. *J Reprod Fertil* **105**, 25-33 (1995).

42 Henriksen, K., Hakovirta, H. & Parvinen, M. Testosterone inhibits and induces apoptosis in rat seminiferous tubules in a stage-specific manner: in situ quantification in squash preparations after administration of ethane dimethane sulfonate. *Endocrinology* **136**, 3285-3291, doi:10.1210/endo.136.8.7628362 (1995).

43 Landis, W. J. The strength of a calcified tissue depends in part on the molecular structure and organization of its constituent mineral crystals in their organic matrix. *Bone* **16**, 533-544 (1995).

44 Chen, L., Jacquet, R., Lowder, E. & Landis, W. J. Refinement of collagen-mineral interaction: a possible role for osteocalcin in apatite crystal nucleation, growth and development. *Bone* **71**, 7-16, doi:10.1016/j.bone.2014.09.021 (2015).

45 Sekita, A., Matsugaki, A., Ishimoto, T. & Nakano, T. Synchronous disruption of anisotropic arrangement of the osteocyte network and collagen/apatite in melanoma bone metastasis. *J Struct Biol* **197**, 260-270, doi:10.1016/j.jsb.2016.12.003 (2017).

46 Wang, J., Ishimoto, T. & Nakano, T. Unloading-Induced Degradation of the Anisotropic Arrangement of Collagen/Apatite in Rat Femurs. *Calcified Tissue Int* **100**, 87-94, doi:10.1007/s00223-016-0200-0 (2017).

47 Raghavan, M. *et al.* Quantitative polarized Raman spectroscopy in highly turbid bone tissue. *J Biomed Opt* **15**, 037001, doi:10.1117/1.3426310 (2010).

48 Ishimoto, T., Nakano, T., Umakoshi, Y., Yamamoto, M. & Tabata, Y. Degree of biological apatite c-axis orientation rather than bone mineral density controls mechanical function in bone regenerated using

recombinant bone morphogenetic protein-2. *J Bone Miner Res* **28**, 1170-1179, doi:10.1002/jbmr.1825 (2013).

49 Glimcher, M. J. Molecular biology of mineralized tissues with particular reference to bone. *Rev Mod Phys* **31**, 359–393 (1959).

50 Nalla, R. K., Kinney, J. H. & Ritchie, R. O. Mechanistic fracture criteria for the failure of human cortical bone. *Nat Mater* **2**, 164-168, doi:10.1038/nmat832 (2003).

51 Hauschka, P. V. & Carr, S. A. Calcium-dependent alpha-helical structure in osteocalcin. *Biochemistry* **21**, 2538-2547 (1982).

52 Yang, Y., Cui, Q. & Sahai, N. How does bone sialoprotein promote the nucleation of hydroxyapatite? A molecular dynamics study using model peptides of different conformations. *Langmuir : the ACS journal of surfaces and colloids* **26**, 9848-9859, doi:10.1021/la100192z (2010).

53 Im, J.-A., Yu, B.-P., Jeon, J. Y. & Kim, S.-H. Relationship between osteocalcin and glucose metabolism in postmenopausal women. *Clinica Chimica Acta* **396**, 66-69 (2008).

54 Fernández-Real, J. M. *et al.* The relationship of serum osteocalcin concentration to insulin secretion, sensitivity, and disposal with hypocaloric diet and resistance training. *J Clin Endocr Metab* **94**, 237-245 (2009).

55 Kanazawa, I. *et al.* Serum osteocalcin level is associated with glucose metabolism and atherosclerosis parameters in type 2 diabetes mellitus. *J Clin Endocr Metab* **94**, 45-49 (2009).

56 Kindblom, J. M. *et al.* Plasma osteocalcin is inversely related to fat mass and plasma glucose in elderly Swedish men. *J Bone Miner Res* **24**, 785-791 (2009).

57 Pittas, A. G., Harris, S. S., Eliades, M., Stark, P. & Dawson-Hughes, B. Association between serum osteocalcin and markers of metabolic phenotype. *J Clin Endocr Metab* **94**, 827-832 (2009).

58 Zhou, M. *et al.* Serum osteocalcin concentrations in relation to glucose and lipid metabolism in Chinese individuals. *Eur J Endocrinol* **161**, 723-729 (2009).

59 Lumachi, F., Camozzi, V., Tombolan, V. & Luisetto, G. Bone Mineral Density, Osteocalcin, and Bone‐specific Alkaline Phosphatase in Patients with Insulin‐dependent Diabetes Mellitus. *Ann NY Acad Sci* **1173**, E64-E67 (2009).

60 Movahed, A. *et al.* Reduced serum osteocalcin concentrations are associated with type 2 diabetes mellitus and the metabolic syndrome components in postmenopausal women: the crosstalk between bone and energy metabolism. *J Bone Miner Metab* **30**, 683-691 (2012).

61 Wang, Y. *et al.* Pyruvate dehydrogenase kinase 4 induces bone loss at unloading by promoting osteoclastogenesis. *Bone* **50**, 409-419, doi:10.1016/j.bone.2011.07.012 (2012).

62 Komori, T. *et al.* Targeted disruption of Cbfa1 results in a complete lack of bone formation owing to maturational arrest of osteoblasts. *Cell* **89**, 755-764, doi:https://doi.org/10.1016/S0092-8674(00)80258-5 (1997).

63 Moriishi, T. *et al.* Osteocyte network; a negative regulatory system for bone mass augmented by the induction of rankl in osteoblasts and sost in osteocytes at unloading. *PLoS One* **7**, e40143, doi:10.1371/journal.pone.0040143 (2012).

64 Yoshida, C. A. *et al.* Runx2 and Runx3 are essential for chondrocyte maturation, and Runx2 regulates limb growth through induction of Indian hedgehog. *Genes Dev* **18**, 952-963, doi:10.1101/gad.117470418/8/952 [pii] (2004).

65 Liu, W. *et al.* Overexpression of Cbfa1 in osteoblasts inhibits osteoblast maturation and causes osteopenia with multiple fractures. *J Cell Biol* **155**, 157-166, doi:10.1083/jcb.200105052155/1/157 [pii] (2001).

66 Umeno, A., Kotani, H., Iwasaki, M. & Ueno, S. Quantification of adherent cell orientation and morphology under strong magnetic fields. *IEEE Trans Magn* **37**, 2909-2911, doi:10.1109/20.951344 (2001).

67 Oliver, W. C. & Pharr, G. M. An improved technique for determining hardness and elastic modulus using load and displacement sensing indentation experiments. *J Mater Res* **7**, 1564-1583 (1992).

Figure legends

Fig 1. Generation of Ocn$^{-/-}$ mice and a μ-CT analysis of femurs.

(A) Schematic presentation of *Bglap* and *Bglap2* gene loci, targeting vectors, and mutated alleles. (B) Southern blot analysis. DNA from tails was digested with SacI and hybridized with the probe shown in A. Bands are indicated corresponding to wild-type (12.8 kb) and mutated (11.7 kb) alleles. (C) Genotyping by PCR. PCR was performed using primers 1, 2, and 3 shown in A. Primers 1 and 3 amplify 706 bp DNA of the wild-type allele, and primers 2 and 3 amplify 980 bp DNA of the mutated allele. (D) Real-time RT-PCR analysis of *Bglap* and *Bglap2* expression using RNA from the osteoblast fractions of femurs in wild-type (wt) and Ocn$^{-/-}$ mice at 9 months of age. Primers were designed to detect the expression of both *Bglap* and *Bglap2*. wt: n=3, Ocn$^{-/-}$: n=7. E, The levels of serum Ocn in female mice at 11 weeks of age. wt: n=5, Ocn$^{-/-}$: n=7. ***P<0.001. (F-I) μ-CT analyses of femurs in male wild-type and Ocn$^{-/-}$ mice at 14 weeks, 6 months, and 9 months of age. μ-CT images of femoral distal metaphyses (F) and mid-diaphyses (G) are shown. Bars = 500 μm. H, Trabecular bone parameters, including the trabecular bone volume (BV/TV), trabecular thickness (Tb.Th), and trabecular number (Tb.N). I, Cortical bone parameters including cortical thickness (Ct. Th), the periosteal perimeter (Ps.Pm), and endocortical perimeter (Ec.Pm). wt (n=12), Ocn$^{-/-}$ (n=6) at 14w; wt (n=14), Ocn$^{-/-}$ (n=24) at 6m; wt (n=7), Ocn$^{-/-}$ (n=9) at 9m. *vs. wild-type mice. ***P<0.001. X symbols in box plots show outliers.

Fig 2. Bone histomorphometric analysis.

(A) Bone histomorphometric analysis of trabecular bone in femurs at 6 months of age. The trabecular bone volume (bone volume/tissue volume, BV/TV), osteoid surface (OS/BS), osteoid thickness (O.Th), osteoblast surface (Ob.S/BS), number of osteoblasts (N.Ob/B.Pm), osteoclast surface (Oc.S/BS), number of osteoclasts (N.Oc/B.Pm), eroded surface (ES/BS), number of osteocytes (N.Oct/BV), mineral apposition rate (MAR), mineralizing surface (MS/BS), and bone formation rate (BFR/BS) were compared between male wild-type (open column, n=7) and Ocn$^{-/-}$ (closed column, n=7) mice. B.Pm, bone perimeter; BS, bone surface. (B and C) Dynamic histomorphometric analysis of the periosteum (B) and endosteum (C) in the mid-diaphyses of femoral cortical bones in male wild-type (n=10) and Ocn$^{-/-}$ (n=12) mice at 6 months of age. * vs. wild-type mice. **P<0.

01. X symbols in box plots show outliers.

Fig 3. Serum markers and real-time RT-PCR.

(A) The levels of serum P1NP, TRAP5b, and CTX1 in wild-type (wt) and $Ocn^{-/-}$ mice at 11 weeks, 6 months, and 9 months of age. Male 11 weeks, wt (n=5) and $Ocn^{-/-}$ (n=6); male 6 months, wt (n=10) and $Ocn^{-/-}$ (n=10); female 6 months, wt (n=6) and $Ocn^{-/-}$ (n=8); male 9 months, wt (n=5) and $Ocn^{-/-}$ (n=6); female 9 months, wt (n=4) and $Ocn^{-/-}$ (n=7). Open column: wt, closed column: $Ocn^{-/-}$. (B) Real-time RT-PCR analysis. RNA was extracted from osteoblast fractions from femurs in male wild-type and $Ocn^{-/-}$ mice at 6 months of age. The values in wild-type mice were defined as 1, and relative levels are shown. wt: n=7, $Ocn^{-/-}$: n=6. **: P<0.01. X symbols in box plots show outliers.

Fig 4. TEM analyses of cortical bone.

(A-F) Immunoelectron microscopic analysis. Ultrathin sections from a wild-type femur at 10 days of age (A, D) and humerus at 4 months of age (B, E), and $Ocn^{-/-}$ humerus at 4 months of age (C, F) were immunolabeled with an anti-osteocalcin antibody and analyzed by TEM. Ocn molecules immunolabeled with 20-nm gold particles were detected in wild-type (A, B, D, E), but not $Ocn^{-/-}$ (C, F) bone. Boxed regions in A, B, and C are magnified in D, E, and F, respectively. (G-J) TEM analysis of non-decalcified sections. The osteoid regions of the metaphyses of tibiae in wild-type (G, H) and $Ocn^{-/-}$ (I, J) mice at 4 weeks of age. The boxed regions in G and I are magnified in H and J, respectively. ob: osteoblast, mbm: mineralized bone matrix. H and J show mineralized nodules in osteoids. Bars: 500 nm (A-C, H, J), 100 nm (D-F), and 5 μm (G, I).

Fig 5. Raman microspectroscopic analysis of femurs in male wild-type and $Ocn^{-/-}$ mice at 14 weeks of age.

(A) A bright field image of the section at mid-diaphysis. The analyzed region is boxed (arrow). (B) Raman spectrum showing the assignments for PO_4^{3-} (959cm^{-1}), CO_3^{2-} (1070 cm^{-1}), Amide III (1243-1320 cm^{-1}), and Amide I (1616-1720 cm^{-1}). (C) Parameters including mineralization, crystallinity, CO_3^{2-}/PO_4^{3-}, collagen maturity, and remodeling were calculated as shown in Table 1. n=6.

Fig 6. BMD and orientations of collagen fibers and the BAp *c*-axis in the femoral cortical bone of female wild-type and Ocn$^{-/-}$ mice at 9 months of age.

(A) Schematic presentation of analyzed positions and the correlation of angles and colors used for collagen orientation in B. (B) The orientation of collagen fibers shown in color. (C) BMD. (D) Collagen orientation degree. If the orientation of collagen fibers is completely parallel to the longitudinal direction of bone, the degree is one. (E) BAp *c*-axis orientation degree. The preferential alignment of the BAp *c*-axis along the bone longitudinal direction was the intensity ratio of the (002) diffraction peak to the (310) peak. Higher values indicate a more preferential alignment to the longitudinal direction. *: $P<0.05$, **: $P<0.01$. (F) Single regression analysis of the orientation of collagen fibers and the BAp *c*-axis. wt: blue dots, Ocn$^{-/-}$: red dots. wt: n=5, Ocn$^{-/-}$: n=6 in C-F.

Fig 7. Crystallite size in the BAp *c*-axis and Young's modulus in femurs of female wild-type and Ocn$^{-/-}$ mice at 9 months of age.

(A) The crystallite size in the BAp *c*-axis in the longitudinal and vertical directions at position 5. (B) Young's modulus along the bone longitudinal axis at position 5 in Fig. 6A as measured by nanoindentation. **: $P<0.01$. X symbols in box plots show outliers. (C) Single regression analysis of Young's modulus to BMD and each degree of the preferential alignment of collagen fibers and the BAp *c*-axis in the bone longitudinal direction at position 5. wt: blue dots, Ocn$^{-/-}$: red dots. wt: n=5, Ocn$^{-/-}$: n=6. (D) Schematic presentation of orientations of collagen fibrils and BAp *c*-axis. In wild-type mice, the orientations of collagen fibrils and BAp *c*-axis are parallel to the bone longitudinal axis. In Ocn$^{-/-}$ mice, the orientation of collagen fibrils is parallel to the bone longitudinal axis, whereas that of BAp *c*-axis is severely disrupted from the longitudinal axis. The disruption of the BAp *c*-axis orientation reduces Young's modulus in the longitudinal axis.

Fig 8. Body weight, blood glucose, HbA1c, and adipose tissues in Ocn$^{-/-}$ mice.

(A)Body weights of male (m) and female (f) wild-type and Ocn$^{-/-}$ mice. Male (wt: n=5, Ocn$^{-/-}$: n=6), female (n=9, 6) at 11w; male (n=25, 18) at 14w; male (n=14, 23), female (n=6, 8) at 6m; male (n=7, 9), female (n=8, 12) at 9m; female (n=8, 6) at 12m; male (n=7, 4) at 18m. (B) Blood glucose levels. Male (wt: n=5, Ocn$^{-/-}$: n=6) at 14w; male (n=16, 16), female (n=8, 13) at 9m; male (n=7, 5) at 18m. (C) HbA1c levels. Male (wt: n=5, Ocn$^{-/-}$:

n=6), female (n=9, 10) at 11w; male (n=14, 18), female (n=6, 7) at 6m; male (n=9, 7), female (n=9, 13) at 9m; male (n=7, 5) at 18m. (D-H) Measurement of adipose tissues. D-G, Sagittal sections at the midline (D, E) and cross sections at the level of L5 (F, G) of μ-CT in male mice at 14 weeks of age. Subcutaneous adipose tissues are shown orange and visceral adipose tissues are shown yellow in the range of the first to fifth vertebra. H, Volumes of subcutaneous and visceral adipose tissues in wild-type (n=10) and $Ocn^{-/-}$ (n=6) male mice at 14 weeks of age and wild-type (n=8) and $Ocn^{-/-}$ (n=5) female mice at 9 months of age. The percentages of adipose tissue volumes against abdominal volumes in the range of the first to fifth vertebra are shown. X symbols in box plots show outliers.

Fig 9. GTTs and ITTs in $Ocn^{-/-}$ mice.

(A) GTT by glucose (2 g/kg body weight) injection. Glucose levels in male mice (wt: n=9, $Ocn^{-/-}$: n=12) at 5 months of age fed a normal diet. (B and C) GTT by glucose (1 g/kg body weight) injection. Glucose (B) and insulin (C) levels in male mice at 9 months of age fed a normal diet. wt: n=9, $Ocn^{-/-}$: n=7. (D and E) GTT by glucose (2 g/kg body weight) injection. Glucose (D) and insulin (E) levels in male mice (wt: n=8, $Ocn^{-/-}$: n=12) at 6 months of age fed a high-fat diet for 1 month. (F) ITT. Glucose levels in male mice (wt: n=20, $Ocn^{-/-}$: n=12) at 4 months of age fed a normal diet. (G) ITT. Glucose levels in male mice (wt: n=7, $Ocn^{-/-}$: n=12) at 8 months of age fed a high-fat diet for 3 months.

Fig 10. Relationships between exercise, glucose metabolism, and bone formation.

(A-D) Body weights (A), HbA1c (B), serum levels of carboxyleted Ocn, uncarboxyleted Ocn, P1NP, and CTX-1 (C), and μ-CT analysis of trabecular bone (D) in male wild-type mice fed a normal diet with or without exercise on a treadmill for 7 weeks. Mice were analyzed at 4 months of age. n=13. (E-H) Body weights (E), HbA1c (F), serum levels of carboxyleted Ocn, uncarboxyleted Ocn, P1NP, and CTX-1 (G), and μ-CT analysis of trabecular bone (H) in male KK/TaJcl mice with or without exercise on a treadmill for 7 weeks. Mice were fed a high-fat diet and analyzed at 4 months of age. Control: n=15, treadmill: n=14. *: $P<0.05$, **: $P<0.01$, ***: $P<0.001$. X symbols in

box plots show outliers.

Fig 11. Analyses of testosterone synthesis and spermatogenesis.

(A and B) Appearance of testes. Bars: 2 mm. (A) and their weights (B) in wild-type (n=5 at 11w, n=7 at 6m, n=7 at 8m) and Ocn$^{-/-}$ (n=6 at 11w, n=7 at 6m, n=11 at 8m) mice. (C) Serum testosterone levels in wild-type (n=4 at 11w, n=12 at 6m, n=6 at 18m) and Ocn$^{-/-}$ (n=3 at 11w, n=15 at 6m, n=4 at 18m) mice. (D) H-E stained sections of the cauda epididymides at 4 months of age. Bars: 10 μm. (E) The number of spermatozoa. The number of spermatozoa in 1 mg of seminal fluid of each mouse at 8 months of age are shown. wt: n=7, Ocn$^{-/-}$: n=11. (F) Morphology of sperm. Sperm were stained with Wright-Giemsa or analyzed with fluorescence microscope after the treatment with Hoechst 33342. Bars: 2 μm. (G) The frequencies of the sperm with acrosomal defects. More than 300 sperm in each mouse at 8 months of age were evaluated using the smears stained with Wright-Giemsa. wt: n=7, Ocn$^{-/-}$: n=11. (H) TUNEL staining. The sections were counterstained with hematoxylin. Bars: 50 μm. (I) The number of TUNEL-positive cells in seminiferous tubules. All tubules (more than 200 tubules) in one section were evaluated in each mouse at 11 weeks of age (wt: n=5, Ocn$^{-/-}$: n=6) and 6 months of age (wt: n=5, Ocn$^{-/-}$: n=4), and the means of the number of TUNEL-positive cells in one tubule are shown. (J) Real-time RT-PCR analysis using RNA from testes at 8 months of age. The values in wild-type mice were defined as 1, and relative levels are shown. wt: n=5, Ocn$^{-/-}$: n=8. X symbols in box plots show outliers.

Fig 12. Analyses of muscles.

(A and B) Body weights (A) and muscle weights (B) in male mice at 4 months (wt: n=5, Ocn$^{-/-}$: n=6) and 8 months (wt: n=7, Ocn$^{-/-}$: n=11) of age. (C and D) H-E stained sections of the quadriceps (C), and the average area of myofibers in the quadriceps (D) of male wild-type and Ocn$^{-/-}$ mice at 4 months of age. The areas of sixty myofibers in the cross sections at mid-diaphyses of femurs were measured. wt: n=7, Ocn$^{-/-}$: n=7. EDL: extensor digitorum longus. Bars: 50 μm. X symbols in box plots show outliers.

Fig 1

Click here to access/download;Figure;Fig 1.tif

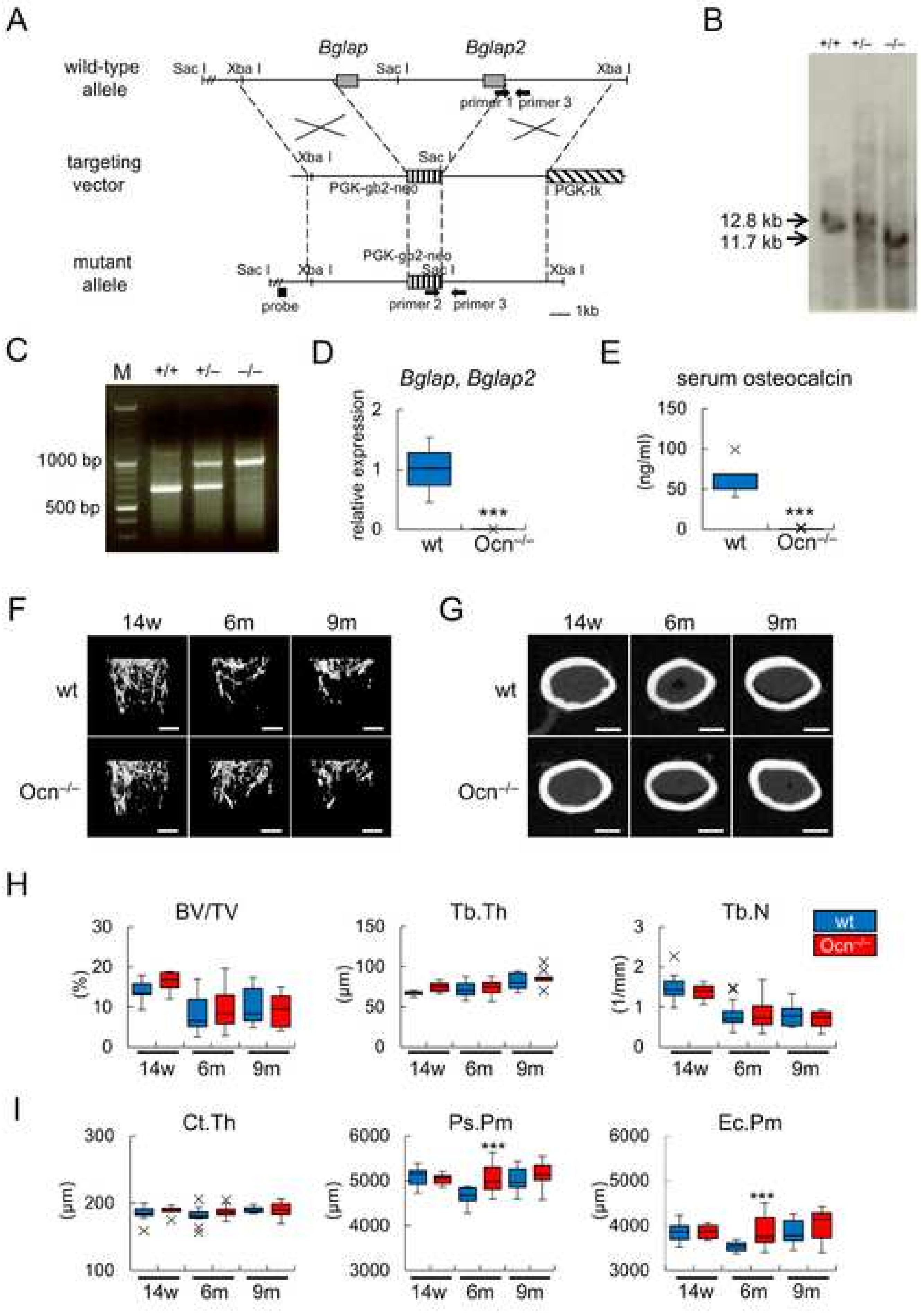

Fig 2 Click here to access/download;Figure;Fig 2.tif

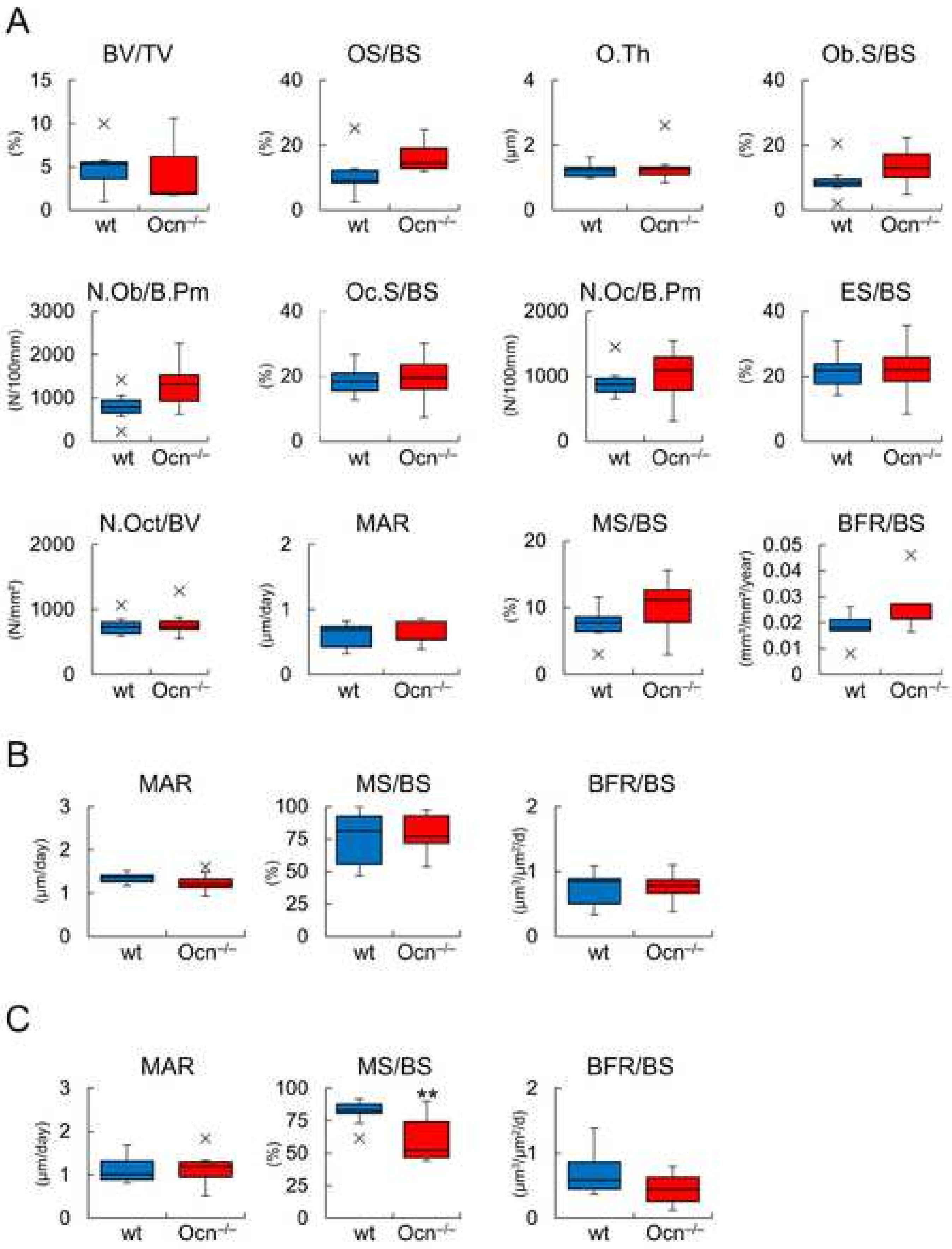

Fig 3

Click here to access/download;Figure;Fig 3.tif

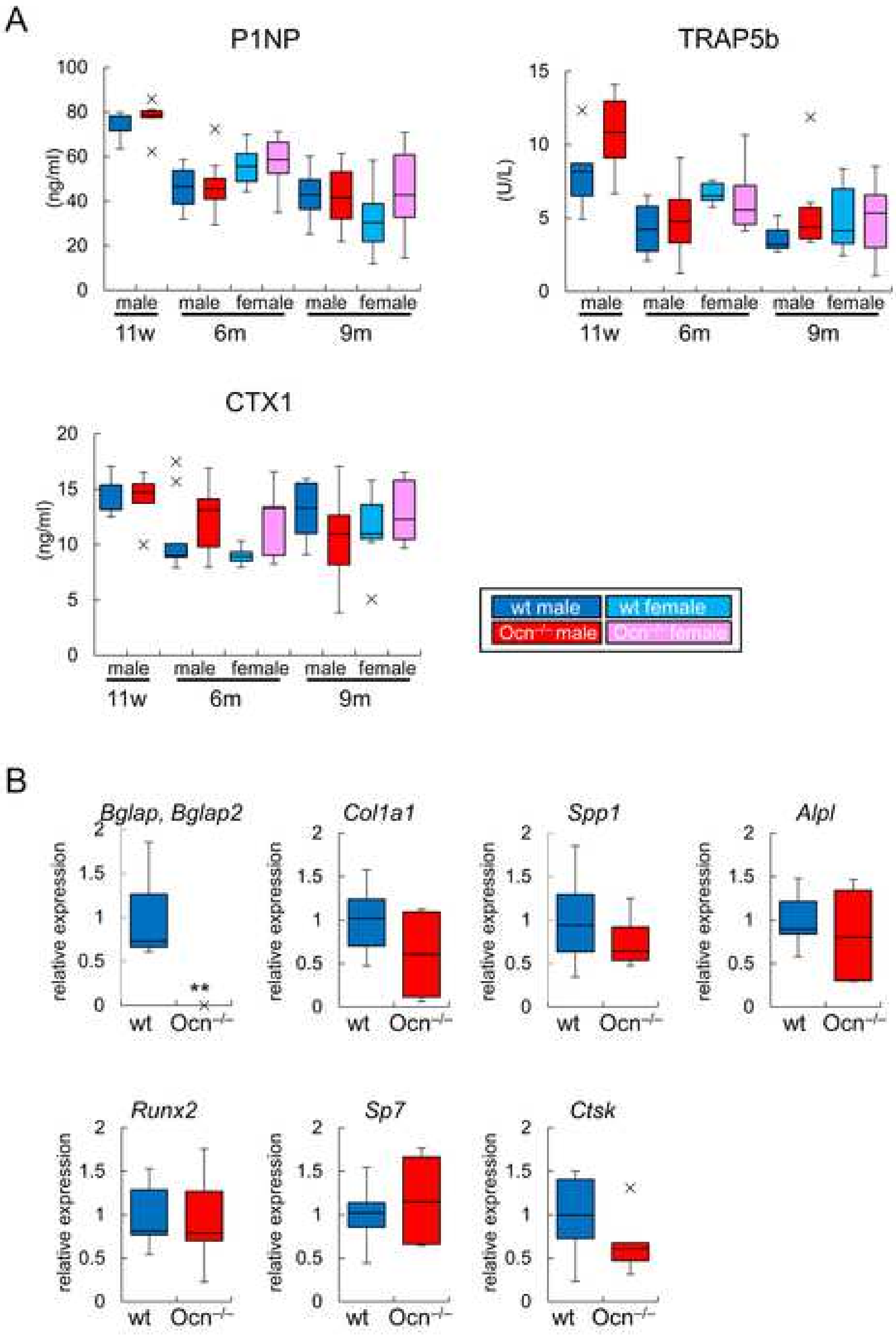

Fig 4

Click here to access/download;Figure;Fig 4.tif

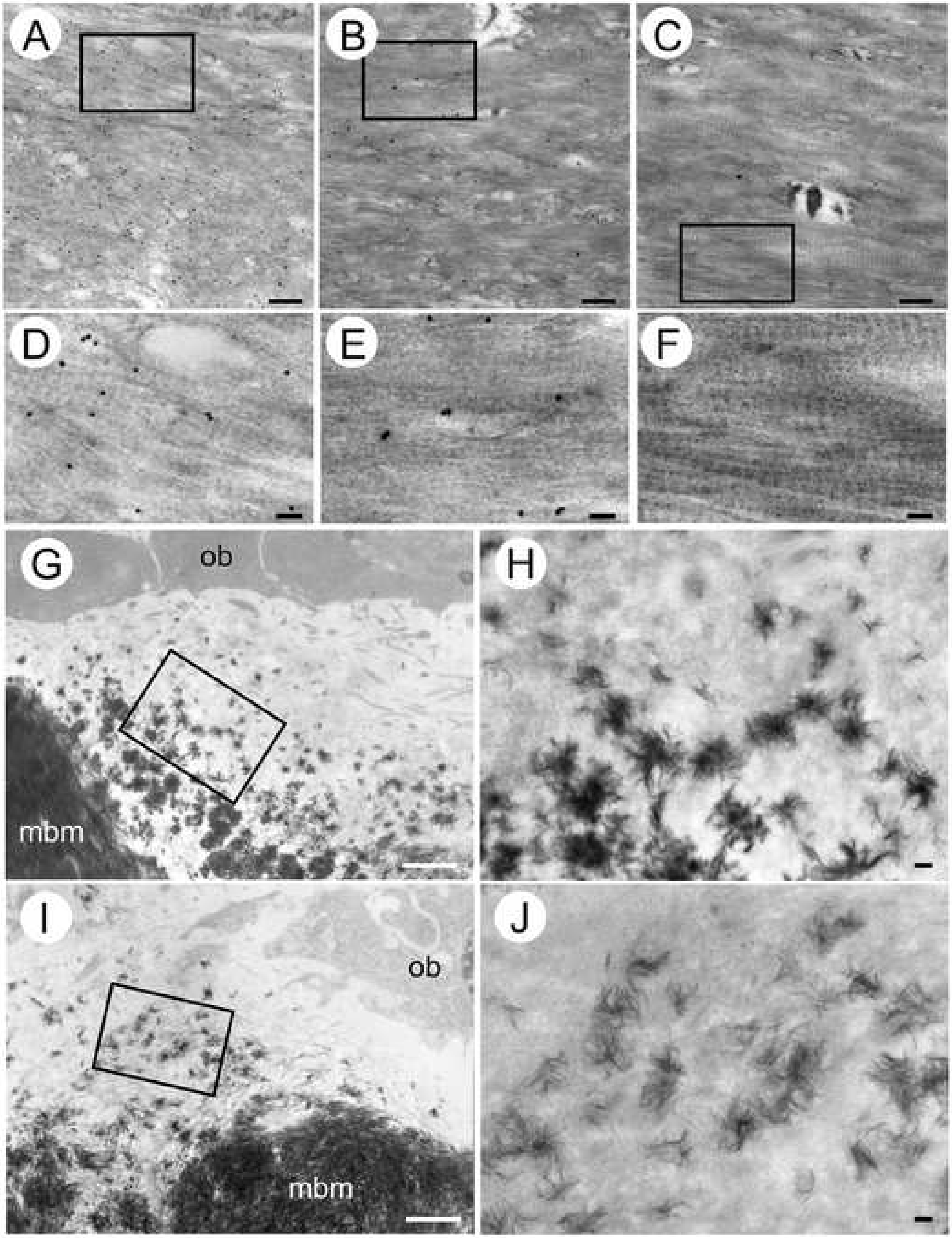

Fig 5

Click here to access/download;Figure;Fig 5.tif

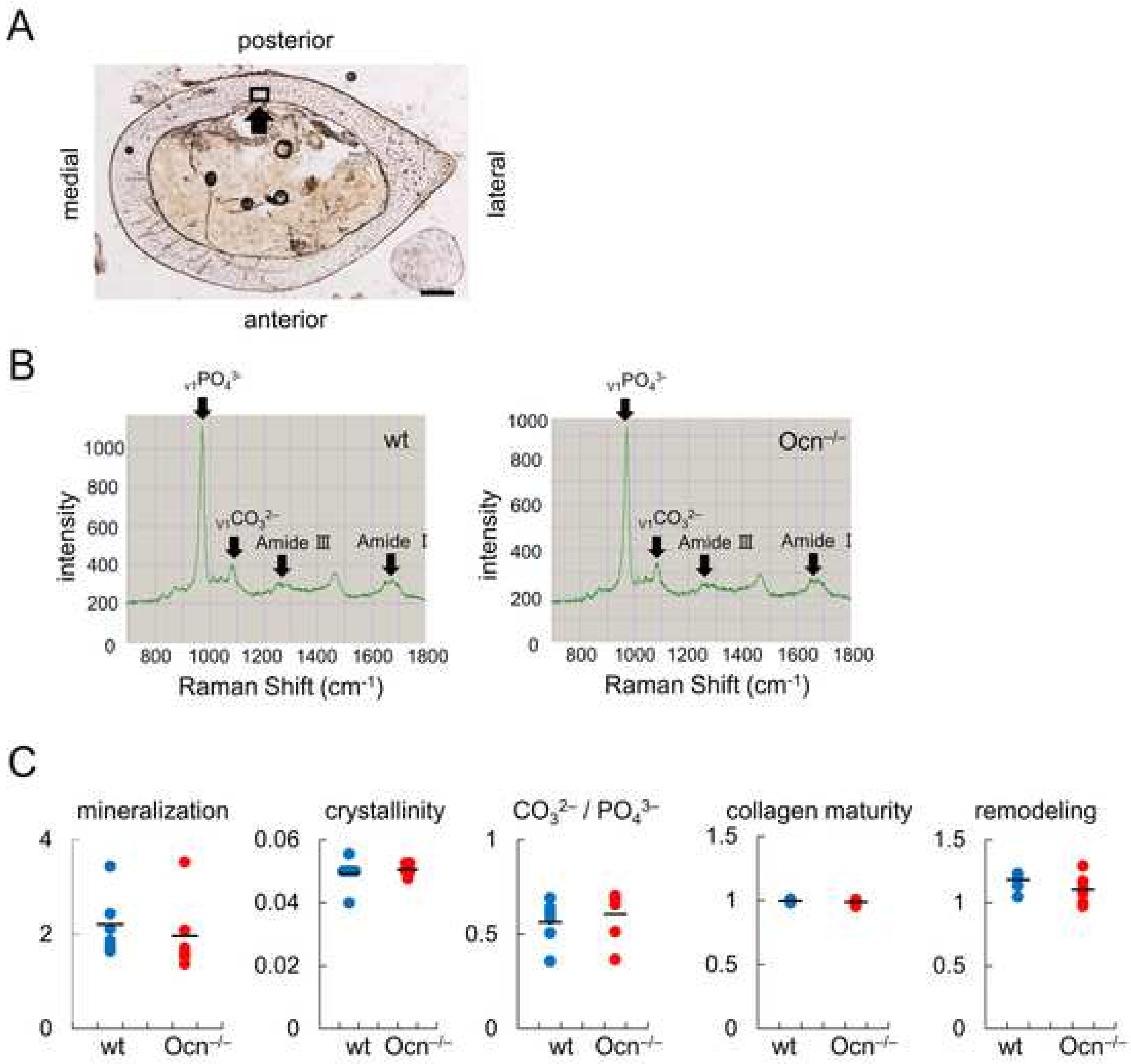

Fig 6

Click here to access/download;Figure;Fig 6.tif

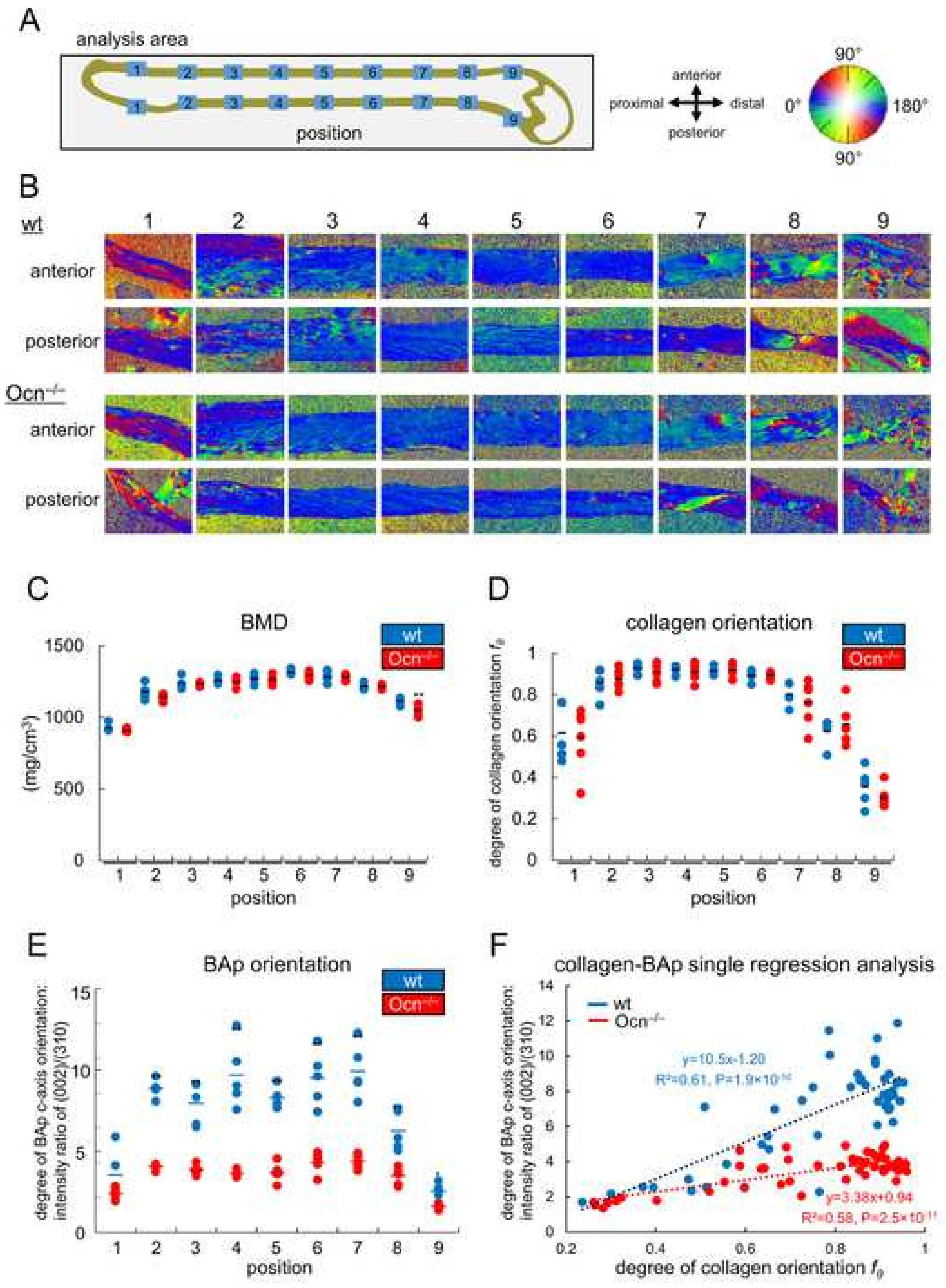

Fig 7 Click here to access/download;Figure;Fig 7.tif

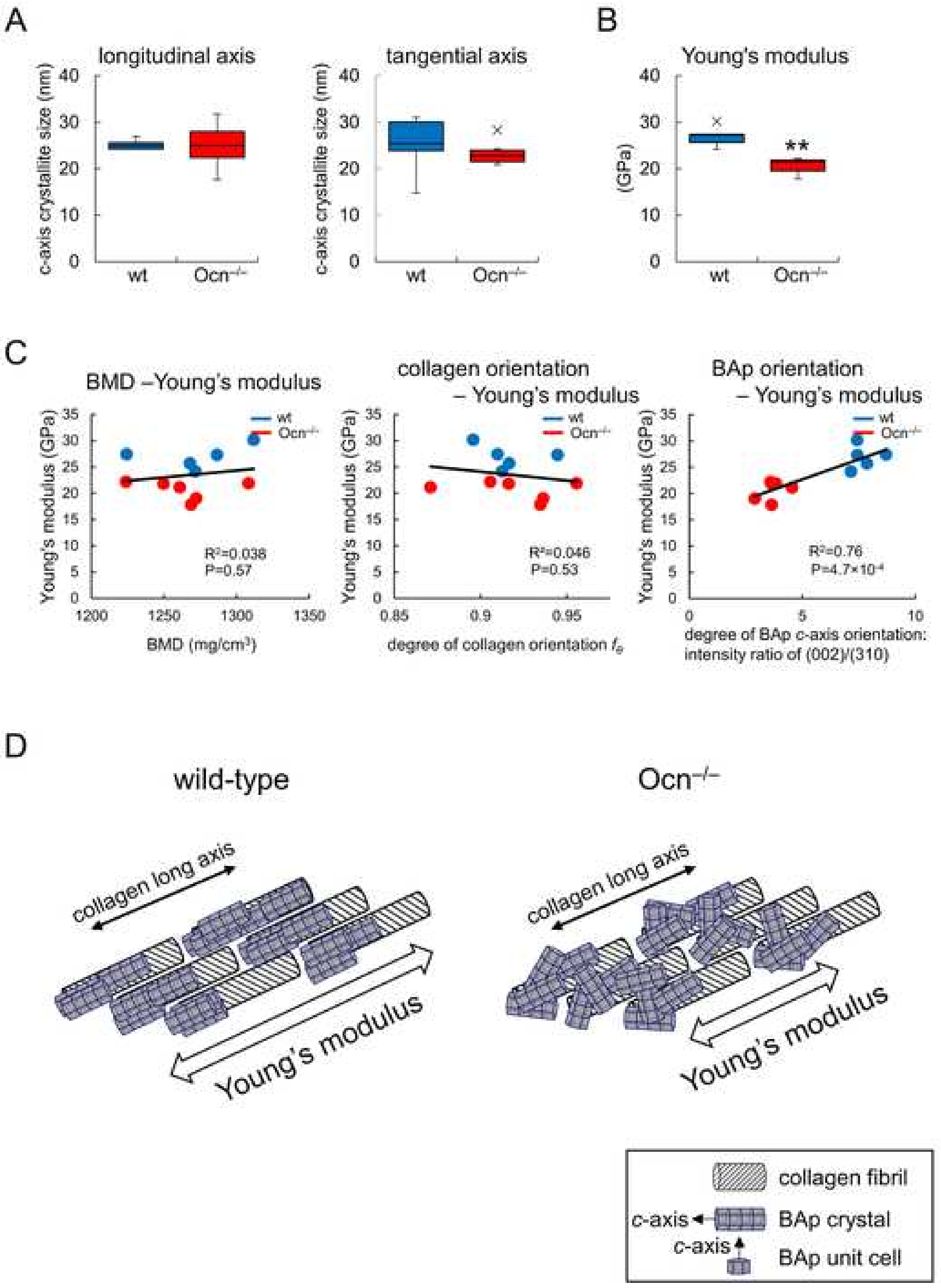

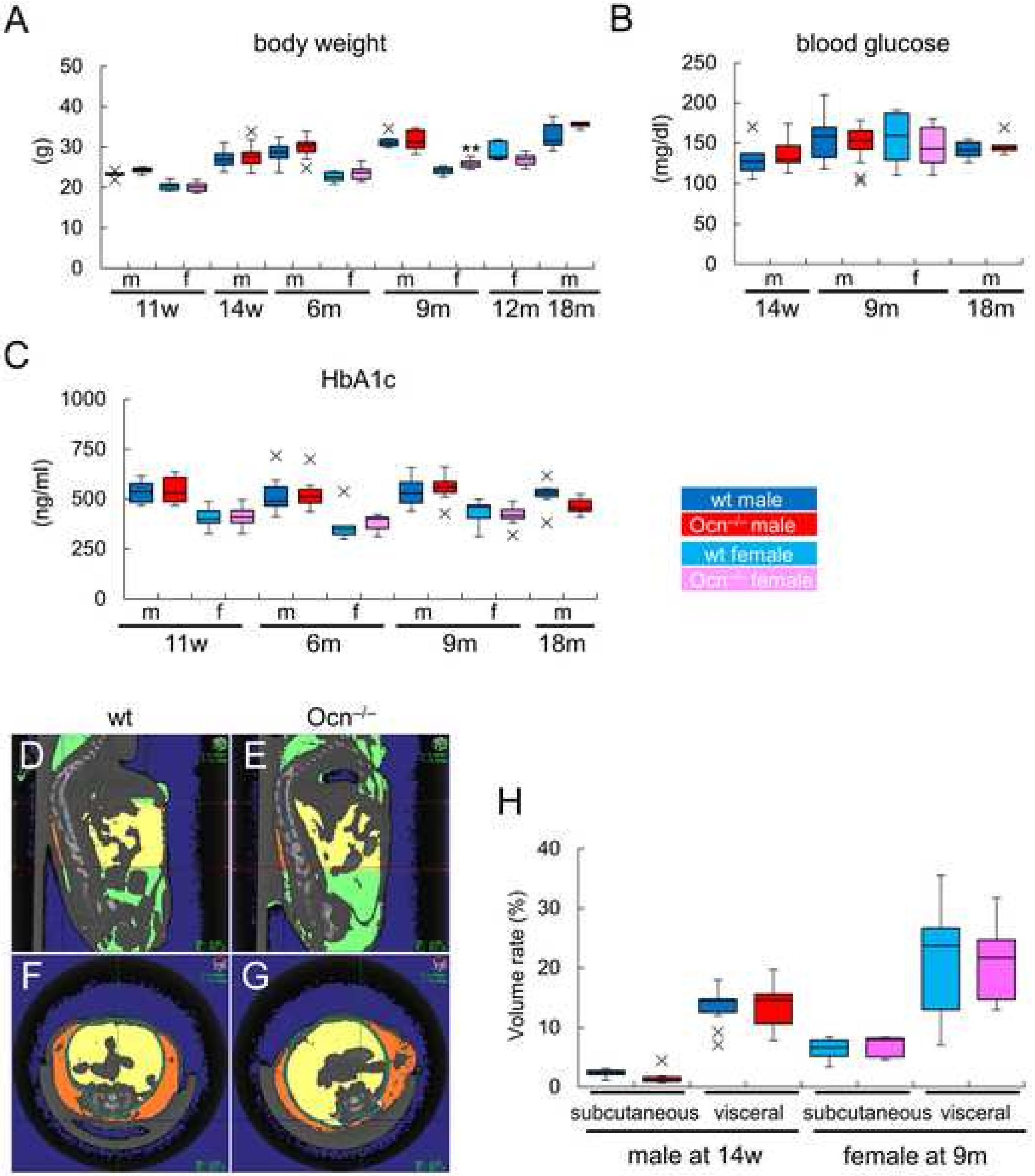
A
body weight
(g)
50
40
30
20
10
0
m
f
m
m
f
m
f
f
m
11w
14w
6m
9m
12m
18m
B
blood glucose
(mg/dl)
250
200
150
100
50
0
m
m
f
m
14w
9m
18m
C
HbA1c
(ng/ml)
1000
750
500
250
0
m
f
m
f
m
f
m
11w
6m
9m
18m
wt male
Ocn-/- male
wt female
Ocn-/- female
wt
Ocn-/-
D
E
F
G
H
Volume rate (%)
40
30
20
10
0
subcutaneous
visceral
subcutaneous
visceral
male at 14w
female at 9m

Fig 9

Click here to access/download;Figure;Fig 9.tif

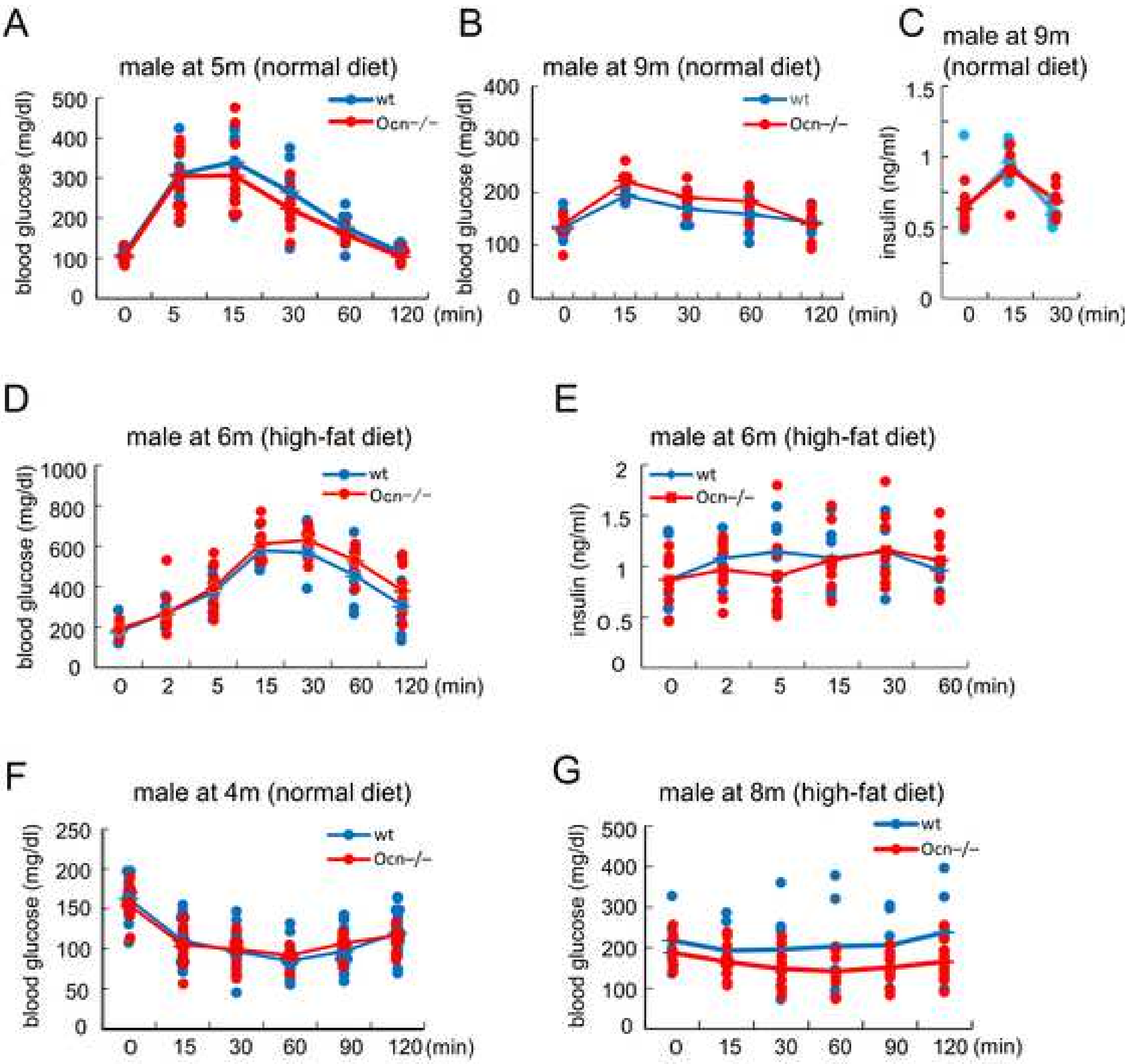

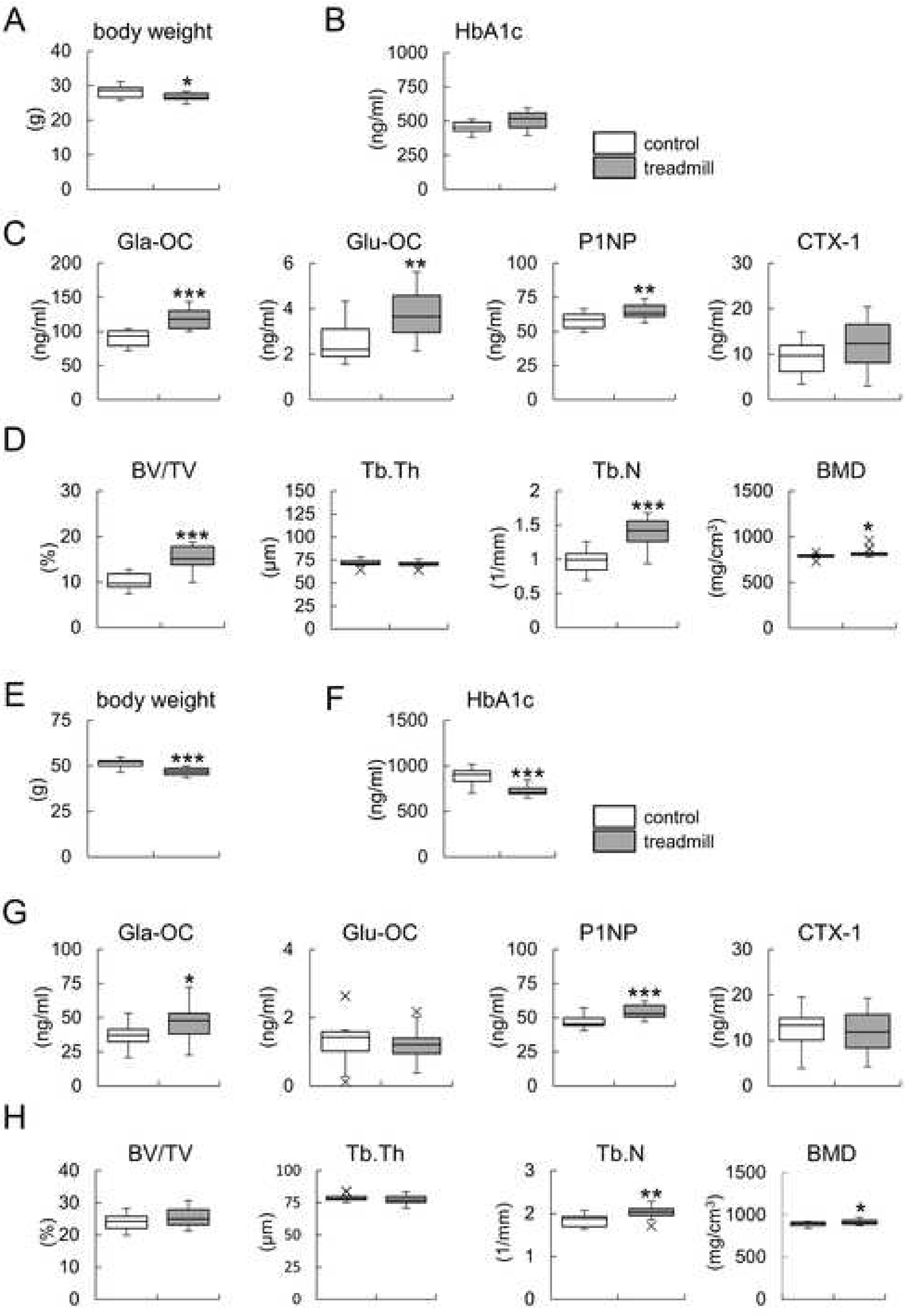
A
body weight
(g)
B
HbA1c
(ng/ml)
control
treadmill
C
Gla-OC
(ng/ml)
Glu-OC
(ng/ml)
P1NP
(ng/ml)
CTX-1
(ng/ml)
D
BV/TV
(%)
Tb.Th
(µm)
Tb.N
(1/mm)
BMD
(mg/cm3)
E
body weight
(g)
F
HbA1c
(ng/ml)
control
treadmill
G
Gla-OC
(ng/ml)
Glu-OC
(ng/ml)
P1NP
(ng/ml)
CTX-1
(ng/ml)
H
BV/TV
(%)
Tb.Th
(µm)
Tb.N
(1/mm)
BMD
(mg/cm3)

Fig 11

Click here to access/download;Figure;Fig 11.tif

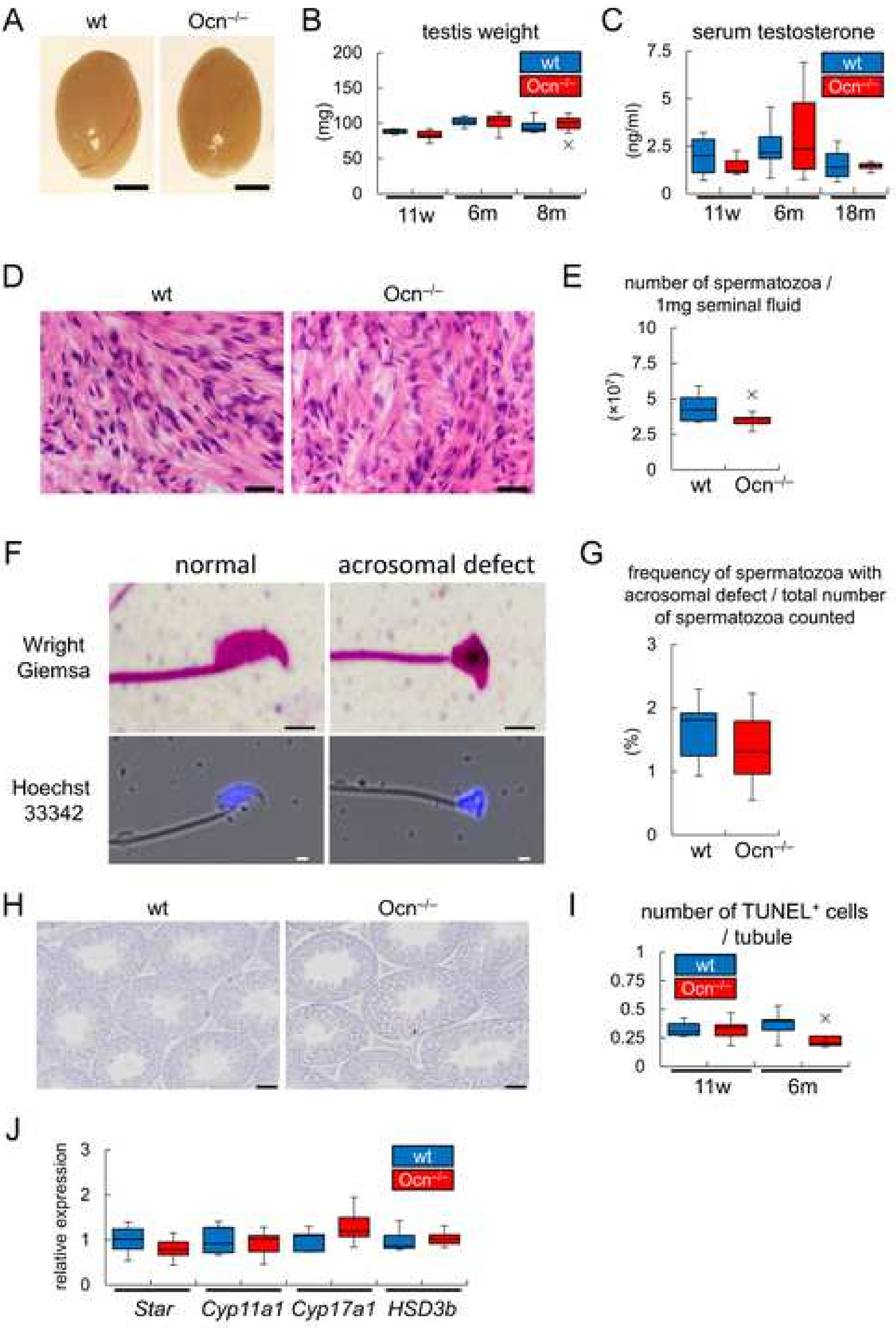

Fig 12

Click here to access/download;Figure;Fig 12.tif

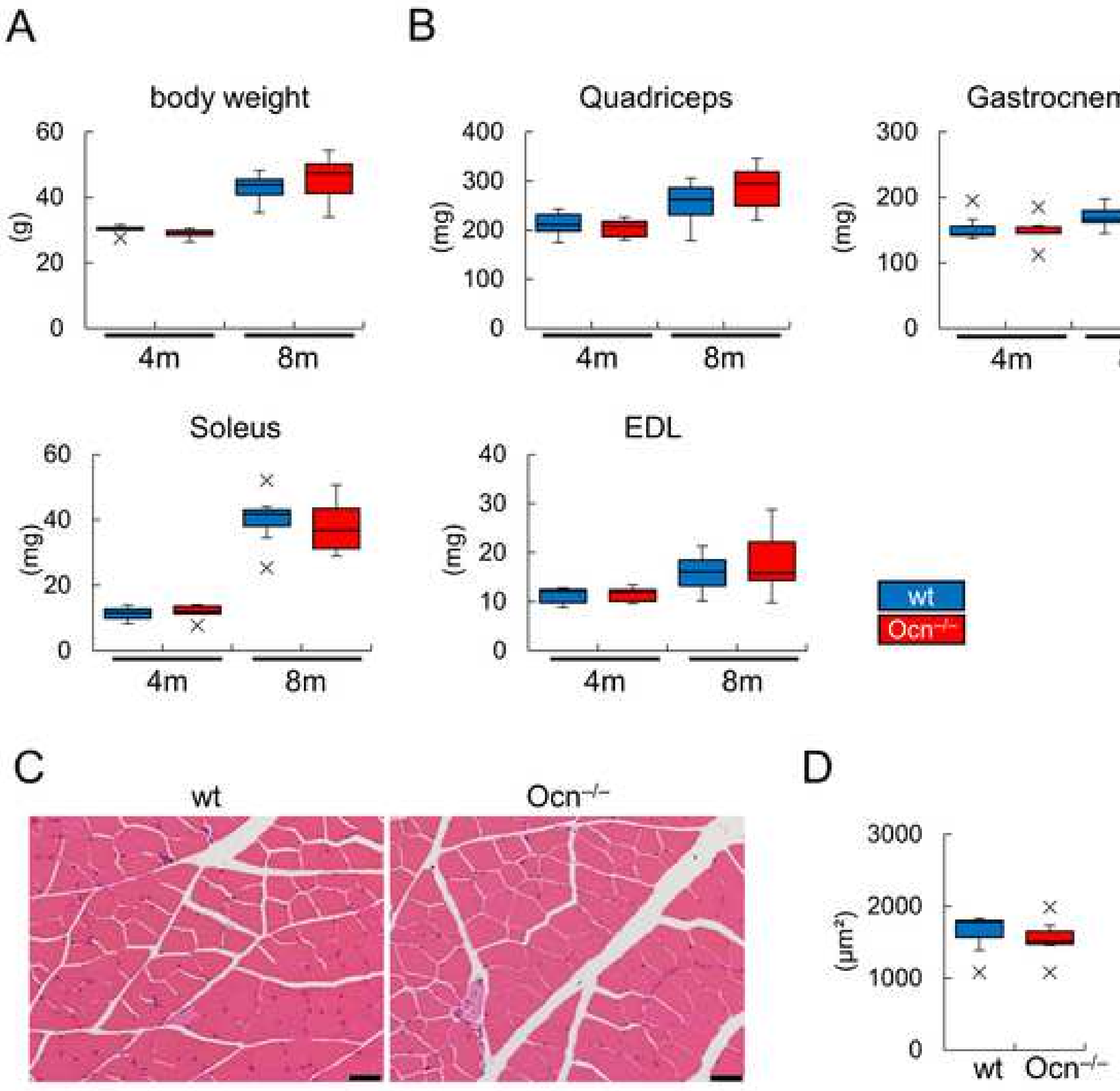

S1 Fig

Click here to access/download
Supporting Information
S1 Fig.tif

S2 Fig

Click here to access/download
Supporting Information
S2 Fig.tif

S3 Fig

Click here to access/download
Supporting Information
S3 Fig.tif

S4 Fig

Click here to access/download
Supporting Information
S4 Fig.tif

S5 Fig

Click here to access/download
Supporting Information
S5 Fig.tif

S6 Fig

Click here to access/download
Supporting Information
S6 Fig.tif

S7 Fig

Click here to access/download
Supporting Information
S7 Fig.tif

S8 Fig

Click here to access/download
Supporting Information
S8 Fig.tif

S9 Fig

Click here to access/download
Supporting Information
S9 Fig.tif
