# Supplementary material for: Osteocalcin is necessary for the alignment of apatite crystallites, but not glucose metabolism, testosterone synthesis, or muscle mass
Source: PLoS Genet. 2020 May 28;16(5):e1008586. doi: 10.1371/journal.pgen.1008586 (PMC7255595; doi:10.1371/journal.pgen.1008586)
Supplement: Attachment — Submitted filename: osteocalcin response to the reviewers 10_13_19.docx [file pgen.1008586.s013.docx]

Response to the reviewers

Reviewer #1: During the last twenty three years, the bone and mineral metabolism research community (and the NIH institutes and numerous other national and international funding agencies that support it) have devoted a great deal of intellectual energy and resources on some very provocative, albeit controversial, ideas about osteocalcin (OCN) – a 46 amino-acid protein that is made in bone and binds avidly to the hydroxyapatite mineral. OCN is produced and secreted almost exclusively by osteoblasts, terminally differentiated cells responsible for the synthesis and mineralization of the bone matrix during the development of the skeleton and its periodic regeneration throughout life. Osteoblasts are short-lived cells originating from mesenchymal progenitors that are replaced depending on the demand for bone formation in a particular location and time. OCN secreted by osteoblasts contains three γ-carboxyglutamic acid residues that are removed by the acidic pH created during the resorption of bone by osteoclasts.

According to ideas originated and propagated by Gerard Karsenty and co-workers, osteoblasts comprise an endocrine organ and decarboxylated OCN is a hormone. The circulating levels of this “new hormone” are, therefore, dependent on the rate of bone turnover, a.k.a. remodeling. Specifically, Karsenty and collaborators have claimed in a series of high profile publications over a period of several years that OCN acts on multiple organs and tissues including bone, pancreas, liver, adipose, muscles, testicles, and the brain to regulates functions ranging from bone mass accumulation, to body weight, adipocity, glucose metabolism, energy utilization, male fertility, mentation, and behavior. Notably, all these claims have been based on the results of studies of a single mouse model with genetic deletion of OCN, generated in the Karsenty laboratory over 23 years ago, but never made available to other investigators seeking to reproduce the results.

However, unlike hormones produced and released by dedicated cells in response to external stimuli, the number of osteoblasts and thereby the circulating levels of osteocalcin inexorably change throughout life as a result of physiologic or pathologic changes of bone itself that can be acute or chronic, systemic or localized, and reversible or irreversible. Examples are skeletal development, growth, adaptation of the skeleton to mechanical forces, fracture healing, changing calcium needs, stress, menstrual cycle, pregnancy, lactation, menopause, aging, hyper- or hypo-parathyroidism, hyperthyroidism, hypercortisolemia, Paget’s disease, bone tumors, etc. Furthermore, medications – approved after extensive trials with thousands of subjects and used by millions for the treatment of osteoporosis – decrease or increase serum osteocalcin levels without any effect on glucose homeostasis, testosterone production, muscles, or behavior. The glaring shortcomings and incongruence of the Karsenty ideas with physiology, pathophysiology, clinical medicine, and pharmacology notwithstanding, the concerns with the hypothesis that bone and osteocalcin regulate many other tissues have progressively intensified over the years by the fact that other groups have failed to reproduce the observations of Karsenty and colleagues in different mice or in a rat model with OCN deletion. Still major journal review articles and prestigious teaching textbooks have continued until now to publish the Karsenty claims as proven biologic facts.

In the article by Moriishi and co-workers, the authors have generated their own OCN deficient mouse, using gene targeting in embryonic stem cells and microinjection into C57BL/6 blastocysts. Using this model they investigated the role of OCN on bone formation and mineralization, as well as glucose metabolism, testosterone production and muscle mass. They report that, in contrast to the results reported by Karsenty and colleagues, OCN plays no role in bone formation (or resorption) and the accumulation on bone mass in the estrogen sufficient or the estrogen deficient state. Instead, OCN is indispensable for the alignment of biological apatite crystallites parallel to collagen fibers. Loss of OCN function had no effect on collagen orientation, which remained normal. Bone strength, however, was decreased in the OCN deficient mice indicating that alignment of crystallites with collagen fibers is one of the elusive determinants of bone quality that together with bone mass determines the ability of bone to resist fractures. Additionally, Moriishi and colleagues show that OCN plays no role in the exercise-induced bone formation, glucose metabolism, the improvement of glucose metabolism caused by exercise, testosterone synthesis and spermatogenesis, or muscle mass.

This is an exceptionally thorough and careful investigation of the effects of OCN on bone as well as the earlier suggestions the OCN acts as hormone to regulate glucose metabolism, fertility, and muscle mass. The methods of analysis are flawless, the results (with the exception of the OVX experiment) are unambiguous, and the conclusions are fully justified and convincing. The writing of the manuscript, however, is suboptimal. There are unnecessary repetitions, stylistic issues and problems with the grammar that need to be addressed so as to improve the readability of this, otherwise, very impressive work.

Specific comments
1. Legend of Figure 1 H and I: In this and the legend of several other figures the X symbol above or below the box plots, presumably outliers, has not been defined.

We indicated that the X symbols in box plots show outliers in the figure legends, and we described the definition of outliers in the last part of Method section on pages 22-23.

2. Supplemental figure 2D: Cortical thickness and remodeling markers were not affected by OVX in the wild type control mice of this experiment (OVX at 5 weeks and sacrifice at 11 weeks). These results are in contrast to the well documented effect of OVX in adult mice that have already reached peak bone mass (4-5 months of age). The authors need to address this discrepancy and acknowledge the likelihood that during the experimental estrogen deficiency in their mice the femurs continued to grow, thereby confounding a meaningful interpretation.

C57BL/6 mouse line is relatively insensitive to OVX, and cortical thickness does not reduce at 6 weeks after OVX in mice ovariectomized at 12 weeks of age and sacrificed at 18 weeks of age (ref. 1). We mentioned it with the reference on page 7. We previously showed that the urinary deoxypyridinoline apparently increase at 1 week after OVX in mice ovariectomized at 4 months of age (ref. 2), and we observed that the levels of TRAP5b were similar between sham and OVX mice at 6 weeks after OVX in C57BL/6 mice ovariectomized at 13 weeks of age in another project (unpublished data). Two Japanese papers also reported that urinary NTX transiently increases at 1-3 weeks after OVX in rats ovariectomized at 12 or 19 weeks of age and returns to a normal level at 18 or 25 weeks of age, respectively. Therefore, we think that serum resorption markers increase at 1-3 weeks after OVX and returns to the normal levels at 6 weeks after OVX in both adult and young mice. In the supplementary figure 2E, we wanted to show that TRAP5b returns to a normal level in both wild type and Ocn^–/–^ mice at 6 weeks after OVX. Since we did not examine TRAP5b at 1-3 weeks after OVX in this experiment, however, we deleted the supplementary figure 2E.

Ref. 1: Klinck, J. & Boyd, S. K. The magnitude and rate of bone loss in ovariectomized mice differs among inbred strains as determined by longitudinal in vivo micro-computed tomography. *Calcified tissue international* **83**, 70-79, 2008.

Ref 2: Maruyama Z, Yoshida CA, Furuichi T, Amizuka N, Ito M, Fukuyama R, Miyazaki T, Kitaura H, Nakamura K, Fujita T, Kanatani N, Moriishi T, Yamana K, Liu W, Kawaguchi H, Nakamura K, Komori T: Runx2 determines bone maturity and turnover rate in postnatal bone development and is involved in bone loss in estrogen deficiency. *Dev Dyn*. 236(7):1876-1890, 2007.

Grammatical and stylistic suggestions:
1. The title should be shortened as follows: Osteocalcin is necessary for the alignment of apatite crystallites, but not glucose metabolism, testosterone synthesis, or muscle mass.

We agree to shorten the title according to your suggestion “Osteocalcin is necessary for the alignment of apatite crystallites, but not glucose metabolism, testosterone synthesis, or muscle mass”.

2. Osteocalcin should be abbreviated throughout the text as OCN. The use of articles, like a, as in “a Real-time PCR analysis” or “ A bone histomorphometric analysis” is unnecessary.

We abbreviated osteocalcin as Ocn in the whole text. The articles were deleted.

3. Abstract: I took the liberty to suggest the following small edits for the sake of clarity and brevity: Osteocalcin (OCN) which is specifically produced by osteoblasts and is the most abundant non-collagenous protein in bone. It has been shown suggested previously that OCN shown to inhibits bone formation. It also and functions as a hormone that to regulates insulin secretion in the pancreas, testosterone synthesis in the testes, and muscle mass. We have generated a strain of OCN deficient mice using gene targeting in embryonic stem cells and microinjection into C57BL/6 blastocysts. However, an analysis of Ocn–/– mice revealed Loss of OCN function had no effect on the regulation of bone quantity, glucose metabolism, testosterone synthesis, or muscle mass in these mice. OCN deletion, however, disrupted the alignment of biological apatite crystallites parallel to collagen fibers and caused loss of strength, indicating that alignment of crystallites with collagen fibers is one of the determinants of bone quality. that together with bone mass determines the ability of bone to resist fractures. Although the orientation degree of collagen fibrils and size of biological apatite (BAp) crystallites in the c-axis were normal in Ocn–/– bone, the crystallographic orientation of the BAp c-axis, which is normally parallel to collagen fibrils, was severely disrupted, resulting in reduced bone strength. These results demonstrate that osteocalcin is required for maintaining bone quality and strength by adjusting the alignment of BAp crystallites parallel to collagen fibrils.

Thank you for the suggestion.

We changed “The fracture risk of bone” to “The strength of bone”, and added a sentence “We generated Ocn-deficient (Ocn^–/–^) mice by deleting *Bglap* and *Bglap2*”.

4. Author Summary: Here again, I’ve taken the liberty to suggest the following small edits. The strength of bone is dependent on both bone quantity and quality. Osteocalcin (OCN) is the most abundant non-collagenous protein in bone. It has been suggested before that OCN inhibits bone formation, and serum osteocalcin has been shown to and works as a hormone that to regulates glucose metabolism, testosterone synthesis, and muscle mass. We have generated osteocalcin-deficient mice, and found that osteocalcin is not involved in the regulation of bone quantity. The major factors that influence bone quality are the alignment of collagen fibers and mineralization. Collagen fibers align parallelly to the longitudinal axis of bone and mineralization occurs by the growth of apatite crystallites parallel to the collagen. We showed that In our osteocalcin-deficient mice collagen fibers aligned normally but apatite crystallites aligned randomly against collagen, resulting in reduced bone strength. Further, we found that all of the data in found that glucose metabolism, testosterone synthesis, and muscle mass are normal in osteocalcin-deficient these mice. We demonstrated conclude from this work that OCN aligns apatite crystallites parallelly to collagen fibers and thereby it maintains bone quality and strength. but that sSerum osteocalcin, however, does not work as a hormone and does not affect regulates glucose metabolism, testosterone synthesis, and muscle mass.

Thank you for the suggestion.

5. Introduction, pages 3-7: This entire section is long, verbose, and unnecessarily detailed. It should be shortened and simplified, for example by using review articles to summarize the earlier findings/claims about OCN.

The introduction was shortened and simplified.

6. Introduction, 1st paragraph: Fracture risk depends on many factors besides the quantity and quality of bone, for example falls. Gla should be defined the first time it is mentioned on line 7, but in any case the use of the terms Gla and Glu is confusing and unhelpful. Carboxylated and de-carboxylated or un-corboxylated OCN are much simpler and easier terms for the reader to follow. Carboxylated OCN has (instead of exhibits) high affinity for (instead of to) Ca.

“Fracture risk” was changed to “The strength of bone” on page 3. We changed Gla osteocalcin to carboxylated Ocn, and changed Glu osteocalcin to uncarboxylated Ocn. We also changed “Gla osteocalcin exhibits high affinity to Ca” to “carboxylated Ocn has high affinity for Ca” (page 3).

7. Introduction, last paragraph: this is an unnecessary repetition of the abstract and summary.

We deleted most of the last paragraph to avoid a repetition.

8. Page 17, last two lines to end of this paragraph on page 18: the way this paragraph is written it implies that the earlier claims are factual. This is confusing. Moreover, several of the thoughts discussed here are repetition of earlier sections.

The following sentences were deleted:

Glu osteocalcin functions as a hormone, which is delivered from bone and regulates glucose metabolism, testosterone synthesis, and muscle mass ^30,47,59,75^. However, these were normal in our Ocn^–/–^ mice, irrespective of the deletion of both *Bglap* and *Bglap2* as the previous Ocn^–/–^ mice. There is a similar and unexplainable discrepancy in the phenotypes of the deletion of *Gprc6a*, which has been shown to encode an osteocalcin receptor that is required for the functions of osteocalcin as a hormone ^47,54,56^. *Gprc6a*^–/–^ mice with the deletion of exon 2 showed impaired glucose metabolism and testosterone synthesis and reduced BMD, whereas *Gprc6a*^–/–^ mice with the deletion of exon 6 or the full locus were normal in glucose metabolism, fertility, and BMD ^49-53^.

Reviewer #2: The authors constructed mice knocked out for the two mouse osteocalcin genes and the DNA between the osteocalcin genes in a manner that appears similar to the strategy initially used by Ducy et al. they show that bone mass, bone histomorphometry, serum markers of bone formation and resorption are similar between WT and KO mice. Using EM analysis of antibody treated sections they show that osteocalcin is located in both the intrafibrillar and interfibrillar regions of collagen in WT mice and not detected in the KO. Raman microspectroscopy also revealed no differences in the collagen and mineral chemical indices. However, the c-axis orientation degree of bone apatite was disrupted in the KO mice compared to WT; this correlated with a lower Yong’s modulus in single and multiple regression analysis, after nanoindentation testing. Three point bending analysis was normal in the KO mice. Glucose and insulin tolerance tests were normal in the KO mice, as was their weight and blood glucose. In normal mice exercise improves glucose metabolism and bone formation. Male KO mice had normal sperm and testosterone levels and were fertile. Indices of muscle mass were normal in the KO. Thus, the authors fail to confirm several of the previous findings from the Karsenty group’s studies of similar mice.

1. The wording of the conclusion at the end of page 18 is too sweeping. The authors show that removal of the osteocalcin gene shows that osteocalcin is not needed for normal bone formation, glucose metabolism, or male testosterone production. This is an important and novel series of observations. But, they did not test and cannot comment on the ability of osteocalcin to regulate bone formation, glucose metabolism, testosterone synthesis or the maintenance of muscle mass. It is always possible that other redundant hormones and paracrine factors can compensate for the absence of osteocalcin in the KO mice. So, the authors need to be more constrained in their summary claim.

Thank you for the suggestion.

We changed the conclusion to describe our findings more precisely as follows:

In conclusion, Ocn is not necessary for the regulation of bone formation or resorption, but is essential for the alignment of the BAp *c*-axis parallel to collagen fibrils and is required to maintain bone strength. However, Ocn does not work as a hormone that regulates glucose metabolism, testosterone synthesis, and muscle mass.

2. Figure 9G. It is not clear what statistical analysis led to the conclusion that the blue dots, which appear to be higher than the red dots, are not statistically different.

Statistical analysis was performed by one-way ANOVA and the Tukey-Kramer post hoc test, and there were no statistical differences in Fig. 9G.

3. Figure 10. There’s no mention I can find of what the asterixes in this figure signify.

We indicated the significance “*: P<0.05, **: P<0.01, ***: P<0.001” in the legend.

4. It might be useful for the authors to comment on why the results from their studies differ from those of the Karsenty group. For example, are the knockouts exactly the same? I can’t tell from a simple comparison of the papers. If some sequence adjacent to the coding regions for osteocalcin have effects on another gene, for example, then perhaps that could explain differences. Or maybe different versions of C57B mice could make a difference.

We described the difference of the two Ocn^–/–^ mouse lines on page 16 as follows:

The phenotypes of increased bone mass, impaired glucose metabolism, and reduced testosterone synthesis and muscle mass in the previous Ocn^–/–^ mice were not observed in our Ocn^–/–^ mice, although both *Bglap* and *Bglap2* were deleted and *Bglap3*, which is expressed in non-osteoid tissues with unknown function, was intact in both Ocn^–/–^ mouse lines. The only difference between the two mouse lines is the genetic background. Our Ocn^–/–^ mice were backcrossed to C57BL/6N more than 8 times before analysis, while the previous Ocn^–/–^ mice were analyzed in the mixed background of C57BL/6J and 129/SV.

Reviewer #3: This is a Herculean manuscript that describes the KO phenotype of a Blglap/Bglap2 KO mouse. The Ocn-/- mouse phenotype is of great interest due to a series of high profile publications on a previous Ocn-/- mouse line, which report a number of key roles for Ocn in regulating fertility and metabolism.

The authors perform a number of studies in great detail, including both male and female mice of different ages in studies of bone structure, glucose metabolism, and fertility. The authors comprehensively demonstrate a lack of phenotype for their Ocn-/- mouse in the above endpoints – bar an effect on alignment of apatite crystals and sequelae.

Overall, I comment the authors and have few, if any, comments on the studies performed. The results seem very clear. Find below some considerations and minor questions:

1. In general the statistical analyses are not well described; please clarify the nature of the ANOVA performed (in some cases it could be one-, two-, or three-way ANOVA), and give the overall P value for the genotype effect (which, looking at the graphs, would be absent for most of the figures). Also, I presume that the “x” symbols on the graphs are either outliers, or limits of the data range beyond which would be outliers? Please clarify – and if outliers, describe the method used to exclude.

Statistical analyses of more than three groups were performed by one-way ANOVA and the Tukey-Kramer post hoc test. The P values of less than 0.05 were considered as statistical significance. The P value of less than 0.05 is shown as *, that of less than 0.01 is shown as **, and that of less than 0.001 is shown as ***. No asterisk means that there is no statistical significance. We hesitated to show non-significant P values (more than 0.05) in all graphs, because our figures contain many graphs, each graph contains many comparisons, and the addition of non-significant P values makes the graphs too busy. We used the box plots to visually show the distribution of numerical data and skewness by displaying the data quartiles and averages. X symbols on the graphs mean outliers. The outliers were defined as data points located outside 1.5 times the interquartile range (IQR, shown as a box) above the upper quartile or those located outside 1.5 times the IQR below the lower quartile. We indicated that the X symbols in box plots show outliers in the figure legends, and we described the definition of outliers in the last part of Method section on pages 22-23.

2. Fig 4 – please clarify how these images were selected for the figure (as relatively few Ocn immunoreactive dots are visible in field).

Completely mineralized regions with some distance from osteoid were selected. Ocn immunoreactivity has been shown to be severely reduced without addition of 2mM Ca^2+^ to the Ocn antibody solution in immunogold electron microscopy (ref). We performed it without the addition of 2mM Ca^2+^. It may be a reason why the number of Ocn immunoreactive dots was relatively low in our analysis.

ref: Chen, L., Jacquet, R., Lowder, E. & Landis, W. J. Refinement of collagen-mineral interaction: a possible role for osteocalcin in apatite crystal nucleation, growth and development. *Bone* **71**, 7-16, (2015).

3. Please comment on the potential role (or lack thereof) of Bglap3 – and any differences, or not, with respect to previous Ocn-/- mice lines?

We commented it on page 16 as follows:

The phenotypes of increased bone mass, impaired glucose metabolism, and reduced testosterone synthesis and muscle mass in the previous Ocn^–/–^ mice were not observed in our Ocn^–/–^ mice, although both *Bglap* and *Bglap2* were deleted and *Bglap3*, which is expressed in non-osteoid tissues with unknown function, was intact in both Ocn^–/–^ mouse lines. The only difference between the two mouse lines is the genetic background. Our Ocn^–/–^ mice were backcrossed to C57BL/6N more than 8 times before analysis, while the previous Ocn^–/–^ mice were analyzed in the mixed background of C57BL/6J and 129/SV.

4. Fig 7 – correlation of YM and Bap c-axis orientation – whilst there is a significant correltion, the points are rather clustered at each end of the regression – so I would interpret with caution.

All wild-type mice showed high degree of BAp c-axis orientation (parallel to longitudinal direction) and high Young’s modulus, and all Ocn-/- mice showed low degree of BAp c-axis orientation (random orientation) and low Young’s modulus. Therefore, there was no mouse to show the intermediate phenotype.

5. Given the controversy in the field, it might be useful if the authors make a written statement that they will share their new mouse line with other researchers?

We described it on page 16 as follows:

Since the difference of the genetic background is unlikely to explain all controversial phenotypes, our Ocn^–/–^ mouse line will be shared with other researchers to resolve the controversies.

**Have all data underlying the figures and results presented in the manuscript been provided?**
Large-scale datasets should be made available via a public repository as described in the *PLOS Genetics* [data availability policy](http://journals.plos.org/plosgenetics/s/data-availability), and numerical data that underlies graphs or summary statistics should be provided in spreadsheet form as supporting information.

Reviewer #1: Yes

Reviewer #2: Yes

Reviewer #3: Yes

PLOS authors have the option to publish the peer review history of their article ([what does this mean?](https://journals.plos.org/plosgenetics/s/editorial-and-peer-review-process#loc-peer-review-history)). If published, this will include your full peer review and any attached files.


If you choose “no”, your identity will remain anonymous but your review may still be made public.


**Do you want your identity to be public for this peer review?** For information about this choice, including consent withdrawal, please see our [Privacy Policy](https://www.plos.org/privacy-policy).

Reviewer #1: Yes: Stavros C. Manolagas, MD, PhD

Reviewer #2: No

Reviewer #3: No
