# Supplementary material for: Osteocalcin is necessary for the alignment of apatite crystallites, but not glucose metabolism, testosterone synthesis, or muscle mass
Source: PLoS Genet. 2020 May 28;16(5):e1008586. doi: 10.1371/journal.pgen.1008586 (PMC7255595; doi:10.1371/journal.pgen.1008586)
Supplement: Attachment — Submitted filename: Respond to the Reviewers 12-3-19.docx [file pgen.1008586.s015.docx]

Respond to the reviewers

Reviewer #1: The revisions of the manuscript have improved its clarity, but few areas need some additional editing, especially in the authors’ summary and abstract. I have taken again the liberty to suggest some editorial and grammatical changes. To facilitate the authors, the manuscript PDF has been edited and attached to the review.

Thank you very much for the revision of our manuscript. We modified our manuscript according to the suggestions. We really appreciate that our manuscript has extremely improved by your suggestions.

Reviewer #2: The authors have addressed my concerns.

Reviewer #3: The authors have addressed the main points from the initial review, however, a couple of clarifications would be useful:

Re the ANOVA used, could the authors please clarify why only one-way ANOVA analysis has been used? To take an example, in Figs 11B and 11C, there are two variables (age and genotype), which implies a two-way ANOVA should be preferred? In respect to the request for P values, the request was NOT for the individual P values for the post-hoc analyses (I agree, this would make the graphs very crowded). The suggestion was that the overall P value for the ANOVA (e.g. effect of genotype) be included in the text or figure legend. Not compulsory, but it provides a level of granularity that compliments the graphical data.

We are sorry that we misunderstood your indication. We thought that the changes among ages, between sexes, and in time course are not important for our analysis. Therefore, we compared the values between wild-type and Ocn-/- mice in each age, time, and sex. Thus, we showed only the statistical results using Student’s *t*-test for the comparison between the two genotypes. We should have noticed our mistake in the statistical method description in your first review. Thank you for pointing out our mistake. We deleted the description “those of more than three groups were performed by one-way ANOVA and the Tukey-Kramer post hoc test.” Indeed, statistical analyses of more than three groups should be performed by two-way ANOVA, as you pointed out. It was also our mistake. We described the statistical method as follows:

Statistical analyses were performed using the Student’s *t*-test for the comparison between wild-type and Ocn^–/–^ mice.

Secondly, re the selection of images (Fig 4). The authors state that "Completely mineralized regions with some distance from osteoid were selected". Just for transparency, it would be useful if the authors can make. statement to the effect that the areas were selected based on an objective criterion, and are not subject to any bias. (FWIW, I am not suggesting that they are - I would just like a statement that confirms their objective selection).

The region about 8 μm from osteoid is shown in A, and those about 2 μm from osteoid are shown in B and C. We described it in the legend of Figure 4.
